# Gene-gene interactions between a *LMNA* variant and common polymorphisms drive early-onset atrial fibrillation

**DOI:** 10.1038/s41467-026-73113-0

**Published:** 2026-05-19

**Authors:** Asia Owais, Hanna Chen, Hammad Farooq, Prisca K. Thami, Kathryn A. McGurk, George J. Powell, Jaime DeSantiago, Talla Abbas, Arvind Sridhar, Arif Pavel, Gregory Webster, Bradley Merrill, James S. Ware, Fu Siong Ng, Dawood Darbar

**Affiliations:** 1 Department of Medicine, Division of Cardiology, University of Illinois Chicago, Chicago, IL USA; 2 Center for Bioinformatics and Quantitative Biology, University of Illinois Chicago, Chicago, IL USA; 3 Richard and Loan Hill Department of Biomedical Engineering, University of Illinois Chicago, Chicago, IL USA; 4 National Heart and Lung Institute, Imperial College London, London, UK; 5 MRC Laboratory of Medical Sciences, Imperial College London, London, UK; 6 Program in Medical and Population Genetics, The Broad Institute of MIT and Harvard, Cambridge, MA USA; 7 Department of Biochemistry & Molecular Genetics, University of Illinois Chicago, Chicago, IL USA; 8 Section of Electrophysiology, Ann & Robert H Lurie Children’s Hospital of Chicago, Chicago, IL USA; 9 Royal Brompton & Harefield Hospitals, Guy’s and St. Thomas’ NHS Foundation Trust, London, UK; 10 Department of Medicine, Penn State College of Medicine, Hershey, PA USA

**Keywords:** Atrial fibrillation, Cardiovascular genetics, Cardiac regeneration

## Abstract

Atrial fibrillation (AF), the most common sustained arrhythmia, has a complex genetic basis; however, the molecular mechanisms linking rare and common variants remain poorly understood. Polygenic risk score (PRS) analysis in the UK Biobank and All of Us cohorts reveals that carriers of protein-altering *LMNA* variants (PAVs) have a significantly higher risk of incident AF than predicted by PRS alone, supporting an additive effect of common polymorphisms and *LMNA* variants. Induced pluripotent stem cell derived atrial cardiomyocytes (iPSC-aCMs) from individuals carrying the pathogenic missense variant p.S143P in *LMNA* exhibit widespread disruption of chromatin architecture and perturbation of atrial gene regulatory networks, particularly at loci harboring AF-associated variants and transcription factors essential for atrial rhythm control and contractility. Clustered Regularly Interspaced Short Palindromic Repeats (CRISPR)-based epigenetic editing validates the function of several AF-associated regulatory elements and their downstream targets. Notably, reduced accessibility at an intronic *SCN10A* enhancer harboring the AF-associated SNP rs6801957 is associated with reduced sodium current in p.S143P iPSC-aCMs. These findings are reproduced in iPSC-aCMs derived from an additional individual carrying a distinct pathogenic *LMNA* variant, supporting a broader mechanism in which rare *LMNA* variants and common polymorphisms converge on shared regulatory networks to influence AF susceptibility and highlighting the value of integrating both in arrhythmia risk assessment.

## Abstract (web summary)

Atrial fibrillation has both rare and common genetic contributors, but how they interact has been unclear. Here, the authors show that pathogenic LMNA variants amplify polygenic risk and disrupt atrial regulatory networks, revealing convergence of rare and common variation on shared chromatin mechanisms driving arrhythmia susceptibility.

## Introduction

Atrial fibrillation (**AF**) is the most common cardiac arrhythmia worldwide and has a strong genetic basis with an estimated common variants heritability of ~22%^1–3^. Both rare and common genetic variants contribute to AF susceptibility^4^. Genome-wide association studies (**GWAS**) have identified more than a 100 common variants associated with AF, whereas whole-genome and whole-exome sequencing studies have uncovered individually rare, high-impact variants^2,5,6^. The development of polygenic risk scores (**PRS**) has improved AF risk prediction, with emerging evidence suggesting that common variants can modulate the penetrance of rare monogenic variants^6–8^. However, the molecular mechanisms underlying these gene-gene interactions and their role in creating an arrhythmogenic substrate remain poorly understood. Defining these epistatic interactions may enable more precise risk stratification and guide personalized therapeutic strategies.

Variants in *LMNA*, which encode the nuclear envelope protein Lamin A/C, are implicated in a spectrum of disorders, including isolated cardiac disease^9^. Clinically, *LMNA* variants are associated with conduction system disease, AF, ventricular arrhythmias, and dilated cardiomyopathy (**DCM**)^9^. AF is highly prevalent and an early manifestation in *LMNA*-associated cardiomyopathy^5,10,11^. Lamin A/C, as a type V intermediate filament, plays critical roles in cytoskeletal and nuclear structural integrity, chromatin organization, and gene regulation. *LMNA* variants can disrupt gene expression through epigenetic mechanisms, including altered chromatin accessibility and genome organization^6,12–14^.

We hypothesized that alterations in chromatin architecture due to a rare *LMNA* variant disrupt atrial gene regulatory networks and chromatin accessibility at AF-risk loci, unmasking the effects of common polymorphisms in cis-regulatory regions and thereby influencing AF susceptibility in carriers of *LMNA* protein-altering variants (**PAVs;** i.e., missense and loss of function). To test this, we conducted chromatin accessibility and gene expression profiling of induced pluripotent stem cell-derived atrial cardiomyocytes (**iPSC-aCMs**) generated from a proband carrying the pathogenic missense variant LMNA: p.S143P. This analysis revealed downregulation of transcription factors (**TFs**) and target genes implicated in AF and identified significantly altered chromatin accessibility at eight regions harboring AF-associated variants, including a single-nucleotide polymorphism (**SNP**) within a known *SCN5A* enhancer^15^.

About 90% of the AF-associated SNPs are in non-coding, potentially regulatory regions of the genome^16^. The causal variants and target genes for the vast majority of these loci remain to be validated. To investigate the functional relevance of these chromatin changes at AF-associated variant regions, we employed clustered regularly interspaced short palindromic repeats (**CRISPR**)-based epigenetic assays to activate(**a**) or inhibit(**i**) variant regulatory regions harboring the variants and tracked changes in the expression of potential target genes. While CRISPRa of the known *SCN5A* enhancer rescued *SCN5A* expression and sodium current, we also experimentally validated the regulatory roles and target genes of additional AF-associated variants that have not been previously characterized. These findings support a model in which *LMNA*-variant-induced alterations in chromatin accessibility amplify the effects of common polymorphisms at AF risk loci, thereby increasing susceptibility to arrhythmia. This study advances our understanding of the genetic architecture of AF by revealing AF risk genes and uncovering how common and rare variants interact to alter atrial gene regulatory networks. Collectively, our findings establish a mechanistic link between *LMNA*-mediated chromatin dysregulation and the modulatory effects of common AF risk variants, providing a mechanistic framework for understanding how genetic variation across the allelic spectrum drives AF. These insights underscore the importance of comprehensive genetic assessment that integrates both rare and common variants, not only to improve risk prediction but also to enable earlier interventions to prevent atrial myopathy and its complications, including stroke.

## Results

### Risk of Incident AF in carriers of *LMNA* protein-altering variants according to PRS

**Fig. 1:**
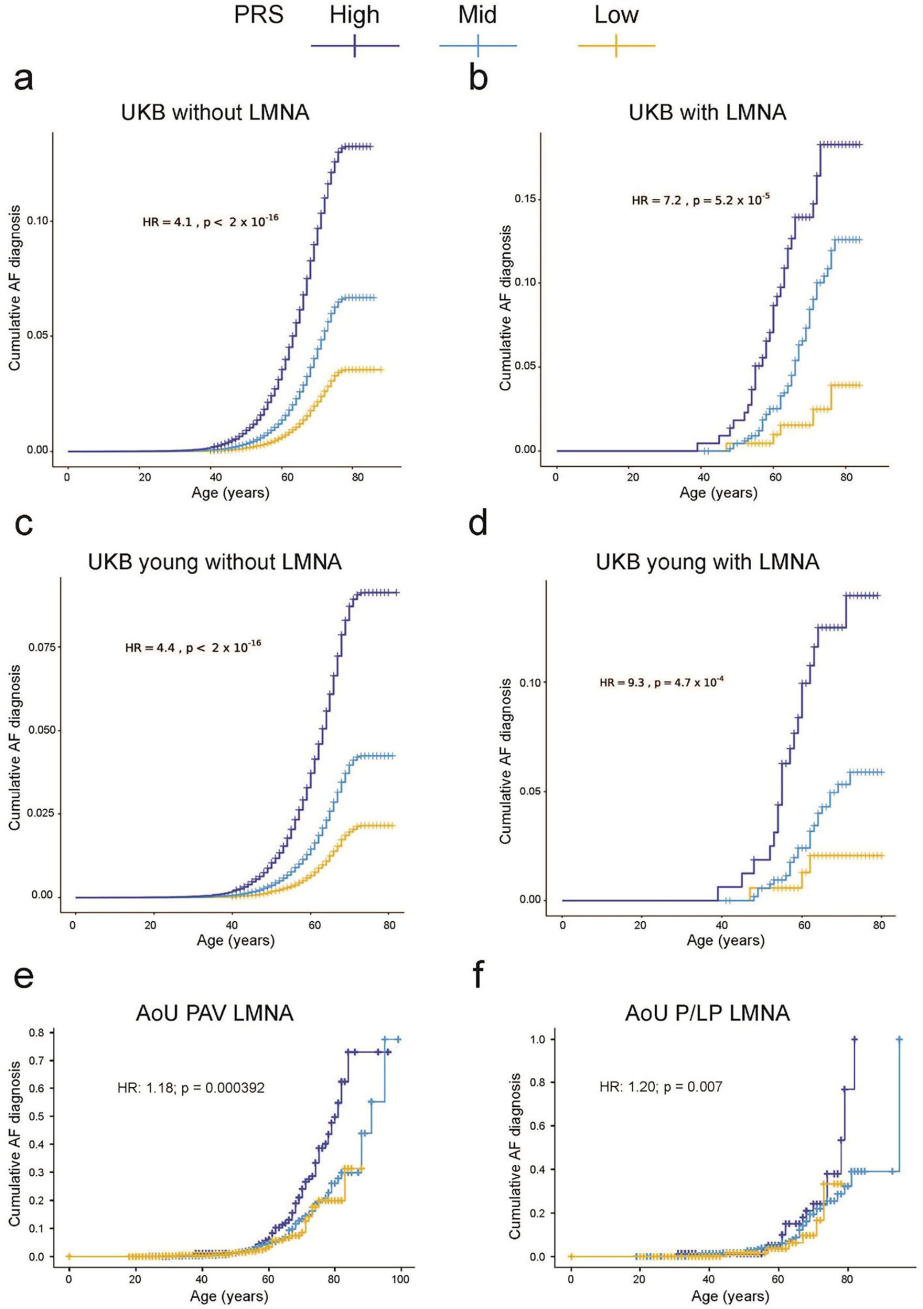
Risk of AF in the UK Biobank (UKB) and All of Us (AoU) cohorts by polygenic risk score (PRS) category. **a** Risk of AF in individuals who do not harbor PAVs in *LMNA* in the UK Biobank by PRS category (*n* = 419,087). A higher PRS is associated with a higher risk of AF in general. **b** Risk of AF in carriers of protein-altering variants (PAVs) in *LMNA* in the UK Biobank by PRS category (*n* = 1109). The risk of AF is doubled in *LMNA* PAV carriers. **c** Risk of AF in young ( < 65 years) participants in the UKB who do not harbor PAVs in *LMNA* by PRS category. **d** The risk of AF in young ( < 65 years) participants in the UKB with PAVs in *LMNA* by PRS category is doubled compared to non-*LMNA* PAV carriers. **e** Risk of AF in carriers of PAV in *LMNA* by PRS category in the AoU cohort (*n* = 2511). A higher PRS is significantly associated with an elevated risk of AF in cases (those with AF) compared to controls (those without AF). **f** Risk of AF in carriers of Pathogenic/Likely Pathogenetic (P/LP) *LMNA* variants in the AoU cohort by PRS category (*n* = 510). A significantly higher risk is observed in cases. The PRS was classified into three categories of genetic risk: low-risk (bottom quintile), medium-risk (middle quintiles), and high-risk (top quintile). (Cox regression was used to calculate the hazard ratio (HR) for AF across PRS categories, adjusting for the first 10 genetic principal components, sex, age, and hypertension. P values: 1a: <2 × 10^-16^,1b: <5.2 × 10^-5^,1c: <2 × 10^-16^ 1 d: 4.7 × 10^-14^,1e: 0.000392, 1 f: 0.007).

We tested whether AF PRS modifies or independently contributes to AF risk among *LMNA* PAV carriers. To assess the contribution of common variants to the variable penetrance of early-onset AF in Lamin A/C cardiomyopathy, we applied a PRS for AF to carriers of PAVs, including missense, frameshift, stop-gained, and in-frame insertion variants in *LMNA*, using data from the UK Biobank and *All of Us* (**AoU**) cohorts. In the UK Biobank, we identified 1109 carriers of 296 distinct rare *LMNA* PAVs, aged 40 to 70 years (median 58 [Q1-Q3: 50–64]), of whom 613 (55%) were female. AF prevalence was higher among *LMNA* PAV carriers (*n *= 89, 8.03%) compared to non-*LMNA* carriers (*n* = 20,917, 4.99%) (*p* = 5.08 × 10⁻⁶). In non-*LMNA* carriers, a high PRS (top quintile) was significantly associated with an increased risk of AF compared to the lowest PRS (bottom quintile) (hazard ratio [**HR**] 4.11, *p* < 2 × 10⁻¹⁶) (Fig. 1a, and Supplementary Table 1). However, the presence of rare *LMNA* PAVs conferred a two-fold increased risk of AF (HR 7.18, *p* < 5.15 × 10⁻⁵), after adjusting for the first 10 genetic principal components, sex, and hypertension (Fig. 1b, Supplementary Table 2).

We evaluated the association between *LMNA* variants and AF using logistic regression, adjusting for the first 10 genetic principal components, sex, age at recruitment, and hypertension in individuals aged 65 or younger. *LMNA* variant carriers had significantly higher odds of AF (odds ratio [**OR**] 1.57, 95% confidence interval [**CI**] 1.12–2.11, *p* < 4.33 × 10⁻³) compared to non-*LMNA* carriers. The odds increased substantially to 8.17 (CI 2.29–29.2, *p* < 1.23 × 10⁻³) in *LMNA* carriers with a high AF PRS compared to those with a low PRS. In a time-to-event analysis, the cumulative risk of AF was significantly higher in younger *LMNA* carriers than in non-carriers (HR 1.56, *p* = 3.01 × 10⁻³) (Supplementary Table 3). High PRS further amplified AF risk, in both *LMNA* carriers (HR 9.31, *p* < 4.73 × 10⁻⁴) and non-*LMNA* carriers (HR 4.4, *p* < 2 × 10⁻¹⁶) in the UK Biobank (Fig. 1c, d, and Supplementary Table 4, 5). The results show that *LMNA* PAVs and PRS contributed independently to AF risk. The combined effect was consistent with an additive model, supported by parallel risk trajectories across PRS strata and no evidence of statistical interaction between the two factors (interaction term *p* = 0.86).

We further analyzed 37 carriers of *LMNA* variants classified as pathogenic/likely pathogenic (**P/LP**) for DCM in ClinVar, of whom six had a diagnosis of AF (16%). Four of these six individuals also had a high-risk AF PRS (Supplementary Table 6). Notably, only one carrier with a P/LP variant had a diagnosis of DCM, and this individual also had a high-risk AF PRS. However, due to the limited sample size of curated P/LP *LMNA* variants in the UK Biobank, we could not calculate precise risk estimates for this group. In the AoU database, we identified 2,511 carriers of *LMNA* PAVs, aged 19 to 102 years (median age: 57). AF prevalence was higher among *LMNA* PAV carriers (7.9%) than among non-*LMNA* carriers (6.18%). Additionally, *LMNA* PAV carriers had significantly increased odds of AF (OR 1.31, 95% CI 1.21–1.51, *p* < 5.38 × 10⁻⁴) relative to non-carriers. Stratification by AF PRS revealed that *LMNA* carriers in the high PRS group had significantly greater odds of AF than those in the low PRS group (OR 3.39, 95% CI 2.10–5.48, *p* < 1.07 × 10^⁻⁷^). Time-to-event analysis using Cox Proportional Hazards modeling, adjusted for the first 10 genetic principal components, sex, and race, further confirmed that a high PRS was associated with an elevated risk of AF (HR 1.18, *p* = 0.000392) (Fig. 1e).

We analyzed a subset of 510 *LMNA* PAV carriers with P/LP variants annotated in ClinVar, of whom 54 (11%) had AF. Within this P/LP carrier group, individuals with a high PRS had a trend towards higher odds of AF than those with a low PRS (OR 2.7, 95% CI 1.02–7.42, *p* = 0.06). Consistently, time-to-event analysis confirmed that a high PRS was associated with an elevated risk of AF in this subset as well (HR 1.20, *p* = 0.007) (Fig. 1f).

### Cardiac phenotype of *LMNA* p.S143P Kindred

**Fig. 2:**
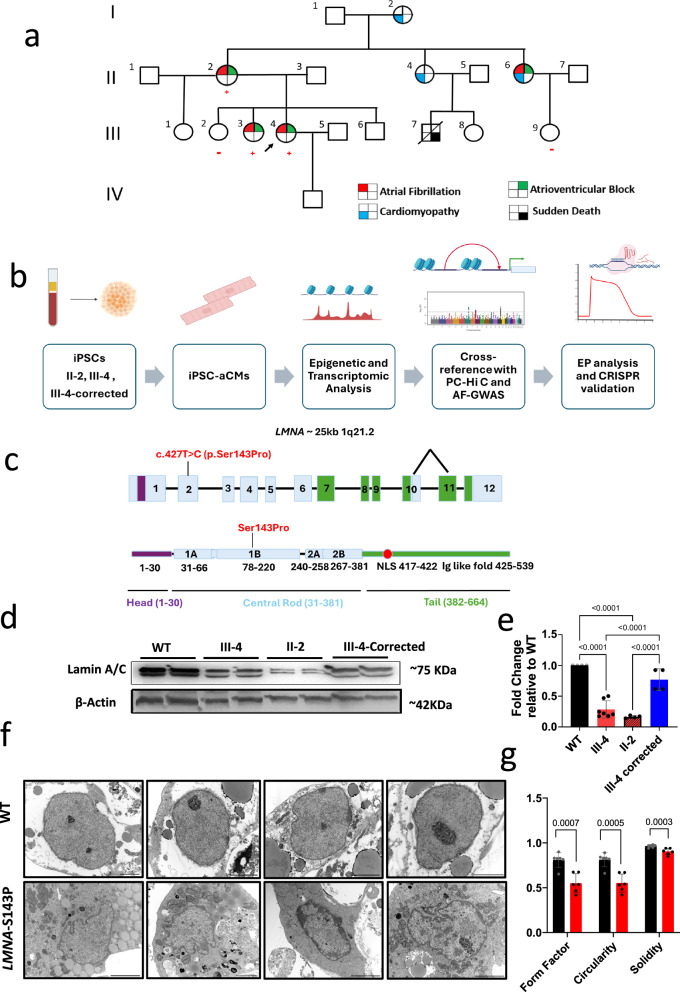
*The LMNA* p.S143P variant reduces expression of Lamin A/C and alters nuclear morphology. **a** Schematic representation of the family pedigree, highlighting affected members with a history of atrial fibrillation (AF), atrioventricular (AV) block, cardiomyopathy, and sudden death. The arrow indicates the proband. Circles denote females; squares denote males. The plus (+) and minus (-) signs indicate the presence and absence of the *LMNA* p.S143P variant, respectively. **b** Schematic illustrating the nucleotide and amino acid change (NM_170707.4(LMNA): c.427 T > C [p.Ser143Pro]) in exon 2 and the central rod domain. The arrowhead marks an alternative splice site location for generating Lamin A and C transcripts. **c** Overview of the study design. **d**,** e** Protein expression analysis of Lamin A/C in wild-type (WT), *LMNA* p.S143P, and S143P-corrected iPSC-aCMs (*n* = 4, for III-4, *n* = 6 samples from 3 independent differentiations). Data are presented as mean values +/SD. Ordinary one-way ANOVA with multiple comparisons Significance at *p* < 0.05. Adjusted *p* values: WT vs. III-4 = 0.0035, WT vs. II-2 = 0.0040, III-4 vs. III-4 Corrected= 0.0015, II-2 vs. III-4 Corrected 0.0017) **f**, **g**. Nuclear deformities observed in *LMNA* p.S143P iPSC-aCMs by electron microscopy. Scale bars from original micrographs at 1 μm. Direct Magnification 12000x, Original micrographs provided in source data). (*n* = 6 iPSC-aCMs. Data are presented as mean values +/- SD. Multiple t-tests WT vs *LMNA* p.S143P. *P* values: Form factor=0.0007, Circularity= 0.0005, Solidity= 0.003. 2b *Created in BioRender. Owais, A.* (https://BioRender.com/rqgp5qh).

To identify the molecular mechanisms by which epistatic rare-common variant interactions create a substrate for AF, we performed comprehensive electrophysiological (**EP**) and epigenetic profiling of mature iPSC-aCMs generated from a proband carrying a heterozygous pathogenic missense variant, *LMNA* p.Ser143Pro (Fig. 2a, and Supplementary Fig. 1 and 2). The proband (III-4) is a 44-year-old female of European and Chinese ancestry who developed early-onset AF at 35 years. The patient presented with palpitations and was found to be in AF and initially underwent direct current cardioversion to maintain sinus rhythm, but the persistence of symptomatic AF necessitated pulmonary vein ablation a year later. Notably, diffuse low-amplitude atrial electrograms were recorded during the ablation. The patient, however, continued to experience symptomatic persistent AF with bradycardia and underwent implantation of a biventricular pacemaker at age 41 (Table 1). A transthoracic echocardiogram at the time of the ablation showed evidence of mild atrial myopathy but preserved left ventricular function with an ejection fraction of 56%. Cardiac magnetic resonance imaging also showed an enlarged left atrium, but no evidence of DCM. A detailed family history revealed that multiple family members were affected by cardiovascular disease (Fig. 2a). Most family members began experiencing symptoms in their mid-30s. The maternal grandmother (I-2) of Caucasian ancestry and several of her siblings died due to congestive heart failure (**CHF**) at age 48. Two family members (II-2 and II-6) developed early-onset AF and conduction disease and required pacemakers, while one family member (II-4) underwent heart transplantation due to CHF. The proband’s maternal cousin (III-7) died of sudden death at 37 years. Several family members have also undergone pacemaker implantation in their late 30 s and early 40 s. Genetic testing of the proband confirmed a heterozygous missense pathogenic variant in *LMNA*, located in the second exon. A base substitution from Thymine to Cytosine results in an amino acid change from Serine to Proline (NM_170707.4(LMNA):c.427 T > C [p.Ser143Pro) (Fig. 2b). The variant is classified as “pathogenic” by the American College of Medical Genetics and Genomics guidelines^17,18^. It is also the most frequently reported variant in *LMNA* in the Finnish population^19^. Genetic testing of the affected mother (II-2) and sibling (III-3) confirmed that they also harbored the *LMNA* p.S143P mutation, while cascade screening of the proband’s unaffected sibling (III-2) showed that she was not a carrier.

**Table 1:** Cardiac Phenotype of *LMNA* p.S143P Kindred

	AF	Conduction Disease	DCM	SCD	PPM/ICD	Additional Phenotyping
I-2	-	-	Yes	-	-	Died in the 40 s 5 out of 7 siblings died in their 40 s due to CHF
II-2 +	Yes	Yes	-	-	PPM	
II-4	-		Yes	-	PPM	Heart transplant
II-5	Yes	Yes	Yes	-	CRT-D	
III-2 -	-	-	-	-	-	
III-3 +	Yes	Yes	-	-		
III-4 +	Yes	Yes	-	-	CRT-P	AF, Bradycardia, 1°AVB
III-7	-	-	-	Yes	-	
III-8	-	-	-	-	-	SVT, NSVT
III-9	-	-	-	-	-	POTS
III-5	-	-	-	Yes	-	Died at 37
III-6	-	-	-	-	-	SVT, NSVT
III-9	-	-	-	-	-	POTS

*DCM* Dilated Cardiomyopathy, *SCD* Sudden Cardiac Death, *CRT-D* Cardiac Resynchronization Therapy – Defibrillator; *CRT-P* Cardiac Resynchronization Therapy- Pacemaker, *ICD* Implantable Cardiac Defibrillator, *1°AVB* First degree atrioventricular block, *SVT* Supraventricular Tachycardia, *NSVT* Nonsustained Ventricular Tachycardia, *POTS* Postural Orthostatic Tachycardia Syndrome.

### The *LMNA* p.S143P variant causes haploinsufficiency of Lamin A/C and nuclear deformities

To identify the molecular mechanisms by which epistatic rare-common variant interactions create a substrate for AF, we generated iPSCs from the proband (**III-4**), another affected individual (**II-2**), and CRISPR-corrected the iPSCs from the proband to generate an isogenic control (**III-4 corrected**), as well as an unaffected healthy control wild-type (**WT**). The iPSCs were differentiated into atrial cardiomyocytes and matured for four weeks before analysis (Supplementary Fig. 1 and 2**)**. Protein expression analysis revealed reduced Lamin A/C levels in affected individuals compared to controls, suggesting haploinsufficiency caused by the mutant allele (Fig. 2d, e). Transmission electron microscopy revealed the presence of nuclear deformities in the mutant cardiomyocytes compared to controls (Fig. 2f, g).

### The *LMNA* p.S143P variant causes extensive transcriptional changes and perturbs the atrial gene regulatory network

Lamins anchor chromatin to the nuclear periphery in transcriptionally inactive lamina-associated domains **(LADs**), while also interacting with transcriptionally active chromatin within the nucleoplasm^20^. Variants in *LMNA* can disrupt chromatin organization and influence gene expression through epigenetic mechanisms^21^. To investigate how altered chromatin contacts and accessibility affect gene expression, we performed integrated RNA sequencing (**RNA-seq**) and Assay for Transposase-Accessible Chromatin sequencing (**ATAC-seq**) on *LMNA* p.S143P iPSC-aCMs, their isogenic corrected controls (**S143P-corrected**), and an unrelated healthy control line (WT).

**Fig. 3:**
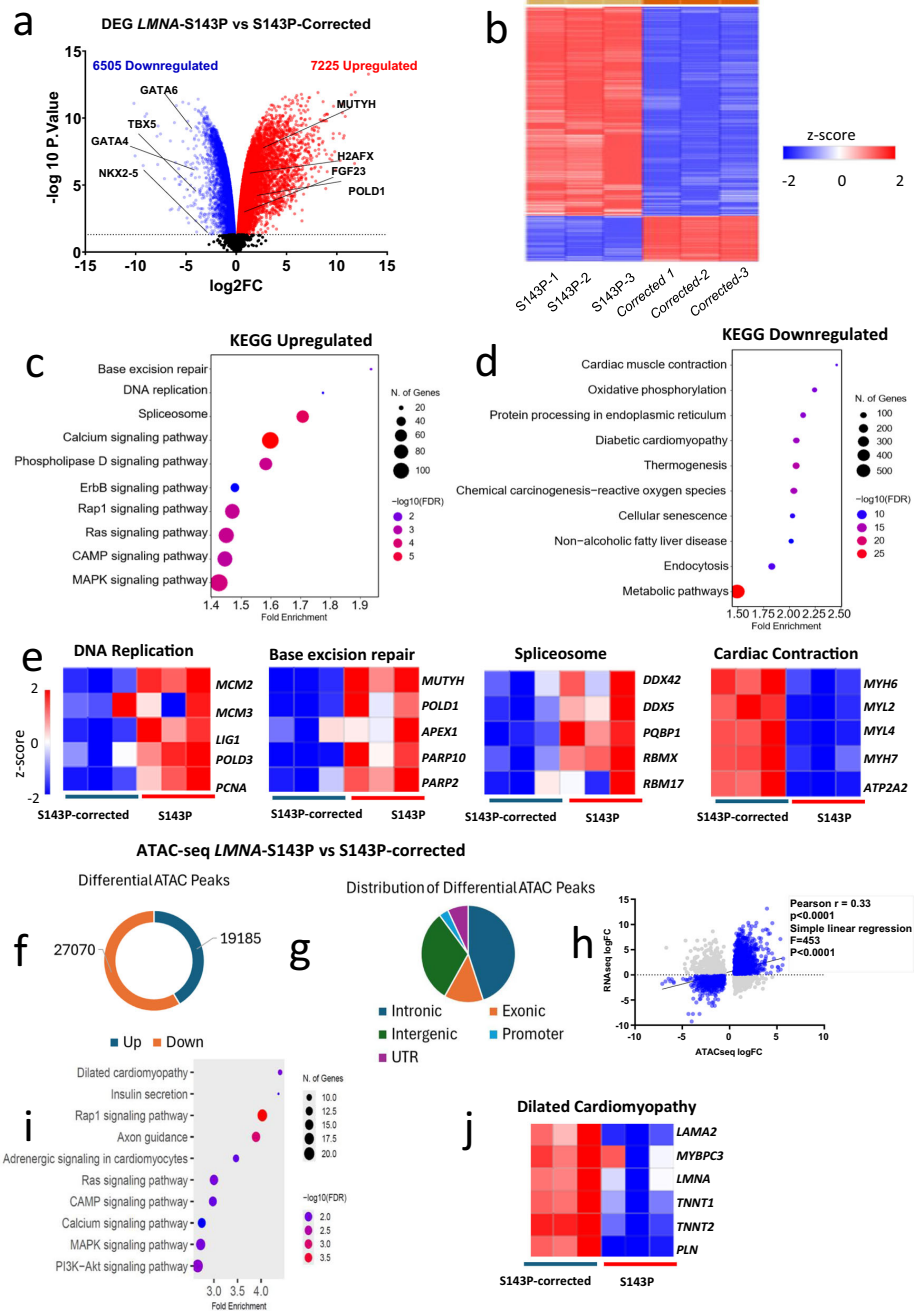
Transcriptomic and chromatin accessibility changes in *LMNA* p.S143P iPSC-aCMs. **a** Volcano plot showing differentially expressed genes (DEG) between *LMNA* p.S143P and CRISPR-corrected isogenic iPSC-aCMs. Significantly upregulated and downregulated genes are highlighted. Significant features at Q < 0.05, log2FC > 0. Data points represent log2FC values for individual genes **b** Clustering heatmap of RNA-seq DEGs in *LMNA* p.S143P iPSC-aCMs. **c**,** d** KEGG pathway enrichment analysis of differentially expressed genes. Upregulated genes are enriched in pathways related to chromatin organization and remodeling, while downregulated genes are associated with cardiac muscle contraction, metabolic dysregulation, and cellular senescence. **e** DEGs in the top enriched pathways. **f** Differential ATAC-seq peaks in *LMNA* p.S143P iPSC-aCMs compared to CRISPR-corrected isogenic controls, indicating widespread changes in chromatin accessibility. **g** *LMNA* p.S143P preferentially alters chromatin accessibility in non-coding regions, including potential regulatory elements such as enhancers and promoters. **h** Correlation of promoter accessibility and DEG in *LMNA*-S13P iPSC-aCMs, Statistical analysis done using Pearson’s correlation (two-tailed *p* < 0.0001) and linear regression, (*p* < 0.0001). **i** KEGG pathway enrichment analysis of genes with altered promoter accessibility identifies dilated cardiomyopathy as the top associated pathway, linking chromatin remodeling to the pathogenesis of cardiac disease. **j** Gene expression alterations in the dilated cardiomyopathy pathway.

Differential gene expression (**DEG**) analysis revealed extensive transcriptional changes, with ~7000 upregulated and ~6000 downregulated genes compared to the isogenic controls (Fig. 3a). Gene enrichment analysis identified that the most significantly upregulated pathways were related to DNA replication and repair alluding to the impact on chromatin architecture due to impaired mechanical stress resilience of the Lamin A/C deficient nuclear lamina (Fig. 2d, g) while the most significantly downregulated pathways were related to cardiac contractility and oxidative phosphorylation, consistent with atrial myopathy and challenged energetics underlying atrial myopathy and AF (Fig. 3c–e).

**Fig. 4:**
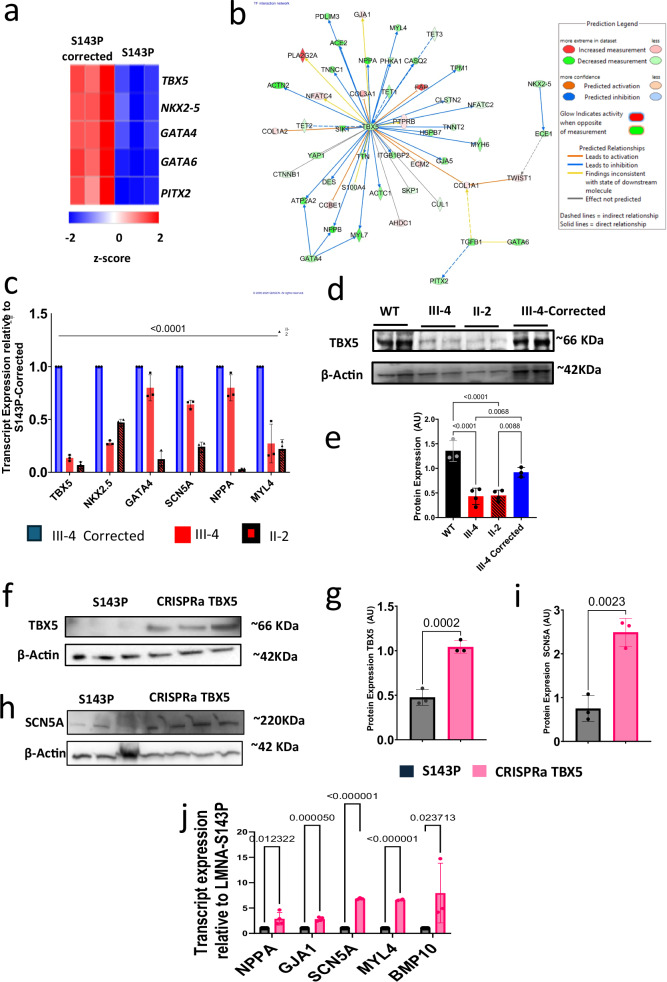
Disruption of atrial transcription factor (TF) network in *LMNA* p.S143P iPSC-aCMs. **a** Expression of TFs regulating atrial cardiomyocyte lineage and implicated in AF. **b** TF interaction network from IPA showing downregulation of transcription factors important for atrial contractility and rhythm control. **c** Expression of *TBX5* and other TFs in the network by qPCR (*n* = 3 biological replicates per group. Multiple unpaired t-tests. *P* values: *TBX5* = *0.000001, NKX2.5* = *0.000009, GATA4* = *0.000038, SCN5A* = *0.000008, NPPA* < *0.000001, MYL4* = *0.000112*) **d**, **e** Protein expression of *TBX5* quantified in d (WT, II-2, III-4, *n* = 4, III-4 corrected, *n *= 3 biological replicates group. Ordinary one-way ANOVA with multiple comparisons, significance at *p* < 0.05. Adjusted *p* values: WT vs. III-4 < 0.0001, WT vs. II-2 < 0.0001, III-4 vs. III-4 Corrected= 0.0068, II-2 vs. III-4 Corrected 0.0068). **f**,** g** Protein expression of TBX5 is increased after CRISPRa (*n* = 3 biological replicates per group)**. h**, **i** Protein expression of *SCN5A* is increased after CRISPRa of *TBX5* (*n *= 3 biological replicates per group) (**j**). CRISPRa rescues atrial-specific transcriptional targets of *TBX5* (WT: *n* = 4, *TBX5* CRISPRa, *NPPA*, *GJA1* n = 4, *BMP10*, *n* = 3, *MYL4*, *n* = 2 biological replicates). Data are presented as mean values +/- SD. Statistical comparison done using multiple unpaired t tests, *q* values: *NPPA* = 0.012, *GJA1* = 0.00005, *SCN5A* < 0.000001, *MYL4* < 0.000001, *BMP10* = 0.023. 4 g: Unpaired t-test, significance at *p* < *0.05*. IPA Ingenuity Pathway Analysis. AU Arbitrary Units.

ATAC-seq-based chromatin accessibility profiling identified ~46,000 differential chromatin accessibility peaks between the two groups, with ~27,000 upregulated and 19,000 downregulated peaks in *LMNA* p.S143P iPSC-aCMs compared to isogenic controls (Fig. 3f). Notably, most of these differential peaks were in non-coding regions (Fig. 3g), suggesting that the widespread changes driven by the *LMNA* p.S143P variant may result from its impact on cis-regulatory regions. To assess the impact of the *LMNA* variant on chromatin accessibility at promoters, we correlated promoter accessibility with gene expression in *LMNA* p.S143P iPSC-aCMs. We observed a moderate but significant positive correlation between promoter accessibility and gene expression (Pearson r = 0.33; *p* < 0.0001; Fig. 3h), indicating that the *LMNA* variant-induced alterations in chromatin architecture may influence the transcription of a subset of genes. Pathway enrichment analysis of the subset of DEGs that showed concordant changes in promoter accessibility and gene expression revealed significant enrichment of pathways associated with impaired contraction and arrhythmogenesis, including dilated cardiomyopathy, adrenergic signaling in cardiomyocytes, and the cAMP signaling pathway (Fig. 3h, i). Transcriptomic and chromatin accessibility profiling of *LMNA* p.S143P iPSC-aCMs relative to the WT population control recapitulated findings from the isogenic control, showing a similar transcriptomic signature of atrial myopathy and widespread alterations in chromatin accessibility across non-coding regions (Supplementary Fig. 3). Collectively, these results suggest that the gene expression changes in *LMNA* p.S143P iPSC-aCMs are partly driven by altered chromatin contacts within regulatory regions of genes implicated in atrial arrhythmogenesis, potentially increasing susceptibility to AF. Many AF-associated loci identified by GWAS have been linked to genes encoding TFs that regulate cardiac development, or to non-coding regulatory regions harboring TF-binding motifs that regulate the expression of genes implicated in AF pathogenesis^16,22^. TF analysis showed that several TFs that collaboratively regulate expression of genes important for atrial contractility and rhythm control, and have been implicated in conduction disorders and familial AF, such as *TBX5*, *PITX2*, *NKX2-5*, *GATA4*, and *GATA6*, were downregulated, suggesting a perturbation of the atrial gene regulatory network (Fig. 4a, and Supplementary Fig. 10)^2,5,23–25^. We focused on TBX5 because it is expressed five times more in the atria than in the ventricles and is known to regulate an atrial enhancer network^26^. Using Ingenuity Pathway Analysis (**IPA**), we performed network analysis, which showed downregulation of *TBX5* and many of its atrial-specific transcriptional targets, such as *NPPA, GJA5, BMP10, MYL4*, and *SCN5A* (Fig. 4b–e)^26,27^. To validate whether the *TBX5* regulatory network was altered in the *LMNA* p.S143P iPSC-aCMs, we CRISPR-activated the *TBX5* promoter, which increased *TBX5* expression and that of its transcriptional targets (Fig. 4f–i). These results support the disruption of atrial gene regulatory networks in *LMNA* p.S143P iPSC-aCMs and suggest that therapeutic activation of *TBX5* may be a promising strategy to restore these TF networks regulating atrial homeostasis.

### The *LMNA*:p.S143P variant in iPSC-aCMs alters chromatin accessibility at AF variant regions

To further elucidate the genetic mechanisms that predispose to early-onset AF, we hypothesized that altered accessibility at AF risk loci may modulate the AF phenotype in carriers of rare LMNA PAVs through epistatic interactions. To test this hypothesis, we cross-referenced differential ATAC peaks with AF-associated sentinel SNPs identified by three recent AF-GWAS^2,28,29^.

**Fig. 5:**
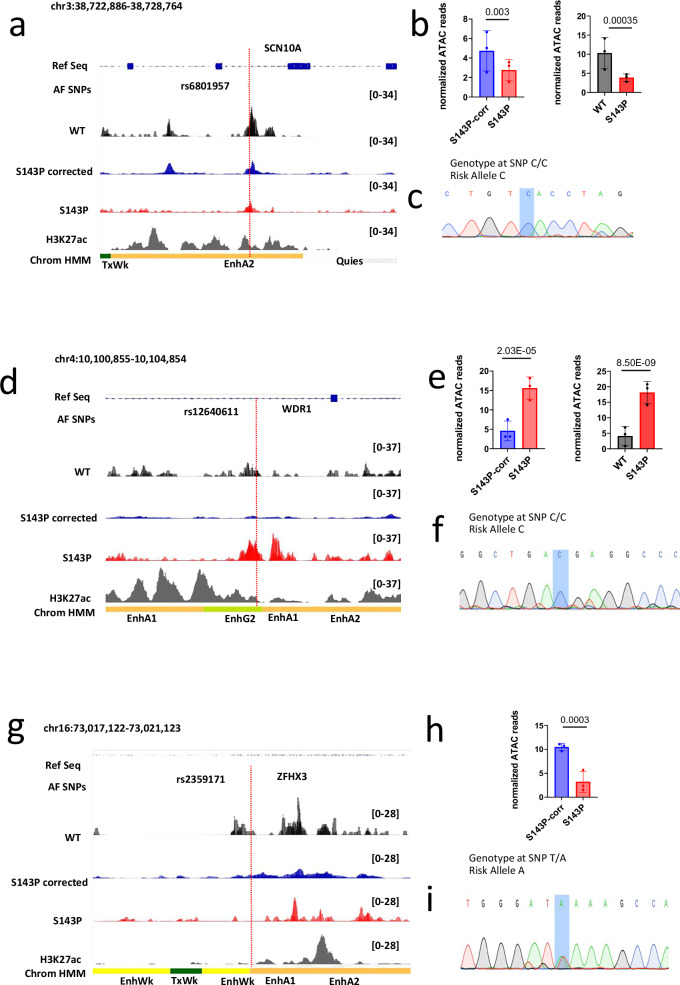
Altered chromatin accessibility at regulatory regions harboring AF-associated SNPs in *LMNA* p.S143P iPSC-aCMs. Integrated genome viewer ATAC-seq track plots, Normalized ATAC reads, and proband genotype at differentially accessible regions with AF-associated SNPs located in regulatory regions marked by H3K27ac and ChromHMM annotations. **a** rs6801957**. d** rs12640611 **g** rs2359171. H3K27ac ChIP-seq track from human atrial tissue (ENCSR074ECR). The dashed line shows the location of the SNP. Genomic location according to GR Ch38/hg38. **b**,** e**,** h** Quantification of normalized ATAC-seq reads, *n *= 3 biological replicates, FDR corrected *P* values displayed, significance threshold at 0.05. **c**, **f**,** i** Proband’s genotype at AF associated SNPs with altered accessibility. SNP single nucleotide polymorphism.

By intersecting AF risk SNPs with regions exhibiting significantly altered chromatin accessibility, we identified eight AF-associated variant regions, all located within non-coding, putative regulatory regions (Fig. 5, Supplementary Fig. 5, Supplementary Table 7 and 8). Notably, comparisons with both the isogenic and WT population controls revealed highly concordant changes in chromatic accessibility at the same variant regions, with two exceptions: rs55734480, which was absent in the isogenic comparison, and rs2359171, which was identified only in the isogenic comparison. The strong concordance between these analyses supported the conclusion that these variant regions likely possess regulatory potential. Genotyping showed that the proband carried the risk allele at six of the eight SNPs, suggesting that the presence of these alleles, together with altered chromatin accessibility, may have a synergistic effect on gene regulation (Fig. 5, Supplementary Fig. 5, Supplementary Table 9 and 10).

To further explore the regulatory function of the AF-associated loci, we intersected our data with H3K27ac ChIP-seq profiles from human atrial tissue (ENCSR074ECR) and integrated ChromHMM annotations.^30^. This analysis revealed that most SNPs reside within regions predicted to function as enhancers, except rs2540949, located in a Transcription Start Site (**TSS**) flanking region, and rs4896104, which lies in a quiescent region (Supplementary Fig. 5a, 5j). We reviewed the literature to gain a deeper understanding of the functional relevance of several loci. Both rs6801957 and rs7172038 have been validated as causal variants through statistical fine-mapping and functional assays^31^. rs6801957 resides within an intronic region of *SCN10A* that functions as an enhancer for *SCN5A* and a short *SCN10A* isoform, which is known to modulate the sodium current (***I***_**Na**_)^32^. rs7172038, located in an intergenic region, has been shown to act as an enhancer for *HCN4*, which encodes the ion channel responsible for the pacemaker “funny current”, *I*_f_^31^. rs2540949 lies in an intronic region of *CEP68* flanking the TSS. To consider any other SNPs in linkage disequilibrium (**LD**) with the sentinel SNPs within differentially accessible regions, we referenced the GWAS catalog. We found that rs23591971, located within an intronic region of the AF-associated TF, *ZFHX3*, is in strong LD with rs2106261 (r^2^ > 0.8), which was determined as causal by fine mapping in a previous study (SNP PIP 0.8, SNP PIP 0.9) (Fig. 5g, and Supplementary Fig. 7f, Supplementary Table 11)^31^.

To gain further insight into the target genes of these candidate regulatory regions, we referenced expression quantitative trait loci (**eQTLs**) for heart tissue on the GTEX portal (version 8.0) and promoter capture Hi-C (**PC-HiC**) data from iPSC-derived cardiomyocytes^33^. Our analysis showed that five variants co-localized with their nearest gene (Supplementary Table 11). Integration of our chromatin accessibility data with published promoter capture Hi-C datasets^33^ identified potential physical interactions between the region containing rs7172038 and the *NPTN* promoter, and between rs4896104 and the *SGK1* promoter (Supplementary Table 11). Deletion of the *NPTN* promoter-interacting region has been reported to alter atrial cardiomyocyte differentiation^34^, whereas functional validation of the *SGK1*-interacting region is still lacking. *SGK1* encodes a serine-threonine kinase that modulates *SCN5A* activity and has been implicated as a therapeutic target for cardiac arrhythmias^35,36^. Collectively, these findings suggest that *LMNA* p.S143P-induced changes in chromatin accessibility may amplify transcriptional dysregulation mediated by common SNPs in AF-susceptibility genes, thereby linking effects of rare and common variants within shared gene regulatory networks.

### Functional validation of candidate variant regulatory regions and target genes at differentially accessible regions in *LMNA* p.S143P iPSC-aCMs

Most of the AF-associated loci identified by GWAS are in non-coding, putative regulatory regions^16^. Due to LD, the sentinel SNP may not always be causal, and the target genes may not be limited to the nearest gene, owing to long-range enhancer-promoter interactions. Therefore, there is a knowledge gap regarding AF-associated causal SNPs and how they modulate the expression of AF-susceptibility genes. Among the differentially accessible regions in *LMNA* p.S143P iPSC-aCMs overlapping with AF SNPs, previous efforts have experimentally validated that rs6801957 and rs7172038 are within enhancers that regulate the expression of *SCN5A*, *SCN10A*, and *HCN4*, respectively^15,31^.

**Fig. 6:**
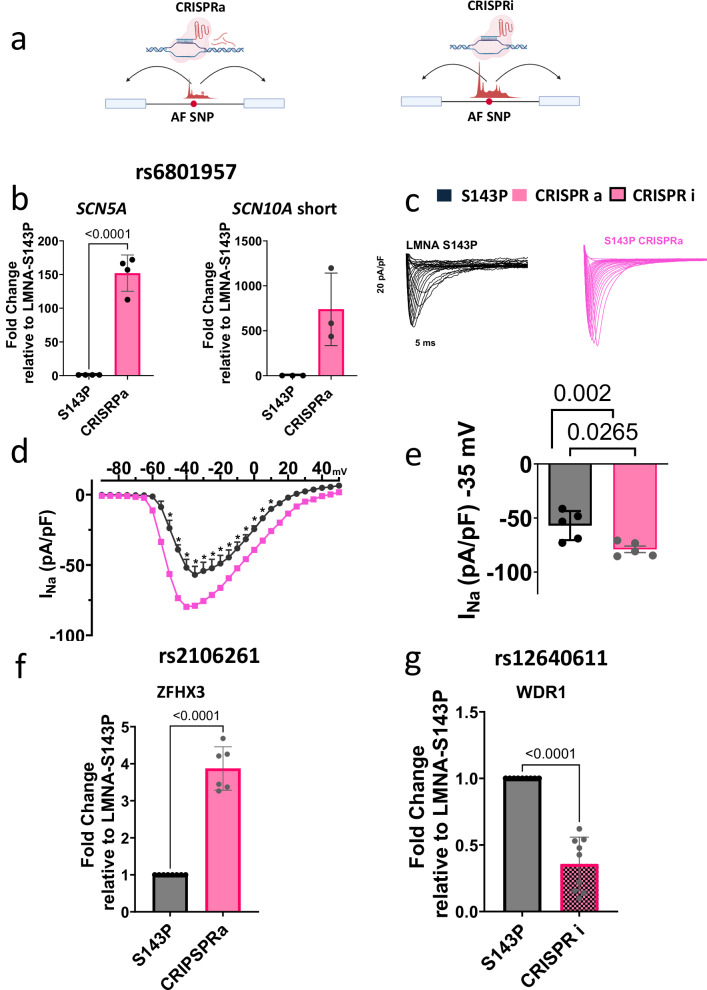
Functional Interrogation of variant regulatory regions in differentially accessible regions by CRISPR epigenetic assays. **a** Schematic showing the CRISPR activation/inhibition design. **b** Reduced accessibility at the *SCN10A* intronic enhancer inhibits *SCN5A* transcription. CRISPR activation(a) restores expression of *SCN5A* and *SCN10A* short; bar graph quantifies changes in expression by qPCR. **b** *n* = 4 biological replicates for each group, representative of three independent differentiations. Unpaired t-test with Welch’s correction. *P* values: *SCN5A* < 0.0001, *SCN10A* short <0.0128). **c** Representative sodium current (*I*Na) traces show that the current is restored after CRISPRa. Current-voltage (I/V) curve showing reduced *I*Na in *LMNA p.S143P* iPSC-aCMs, rescued by transfection of CRISPRa complexes. **d**, **e** Current-voltage (I/V) curve quantified in a bar graph. (**c**–**e** *n* = 4iPSC-aCMs per group) **f** CRISPRa of the ZFHX3 variant region increases *ZFHX3* expression. **g** CRISPRi of the enhancer in *WDR1* reduces its expression. (**f**, **g** *n *= 6 replicates per group. Statistical comparisons done using unpaired t-test with Welch’s correction. *P* values: *ZFHX3* < 0.0001, *WDR1* < 0.0001). Data are displayed as mean +/- SD. a Created in BioRender. Owais, A. (https://BioRender.com/xntq64i).

To functionally characterize the differentially accessible regions overlapping with AF-associated variants in *LMNA* p.S143P iPSC-aCMs, we modulated accessibility at these candidate regulatory regions by CRISPR activation/interference (**a/i**), depending on whether accessibility was decreased or increased. Then we measured the expression of their predicted target genes (Fig. 6).

To validate our approach, we first targeted the well-established enhancer-harboring rs6801957, which regulates the expression of *SCN5A* and a short isoform of *SCN10A*. CRISPR activation of this enhancer increased the expression of *SCN5A, SCN10A* short, and the sodium current (Fig. 6a–e). We then interrogated the regulatory roles of additional AF-associated variant regions that showed altered accessibility and in which the proband carried the risk allele. CRISPRa of a predicted intronic enhancer in *ZFHX3*, harboring the sentinel SNP rs23591971 and the linked causal SNP rs2106261, increased the expression of *ZFHX3*, consistent with its established role as a key cardiac transcription factor strongly implicated in AF^37^ (Fig. 6f). CRISPRi of a predicted intronic enhancer within *WDR1* markedly reduced *WDR1* expression, confirming that this variant resides in a functional enhancer regulating *WDR1* expression (Fig. 6g). WDR1 protein promotes actin filament disassembly, potentially amplifying the cytoskeletal abnormalities associated with the *LMNA* variant. Significantly, CRISPRi of this region did not alter expression of the nearby GWAS-implicated gene *SLC2A9* (Supplementary Fig. 6)^38^.

CRISPRa of candidate regulatory regions harboring the SNPs, rs2540948, rs4896104, and rs1769758 did not increase the expression of the implicated nearby genes *CEP68*, *SGK1*, *ALDH8A1*, and *ZIMZ1*, respectively, suggesting that either these regions may have alternate regulatory functions than enhancer, such as gene silencers or there may be other causal variants in LD with these SNPs (Supplementary Fig. 6).

Overall, our approach not only validated a previously known enhancer in *SCN10A* but also functionally characterized two regions regulating the expression of *WDR1* and *ZFHX3*. Collectively, these results suggest that chromatin alteration due to the *LMNA* variant at these common variants may have amplified their effects on sodium channel dysfunction and cytoskeletal remodeling, demonstrating how the combined contribution of rare and common variants can increase susceptibility to AF.

### Reduced sodium current underlies EP alterations in *LMNA* p.S143P iPSC-aCMs

**Fig. 7:**
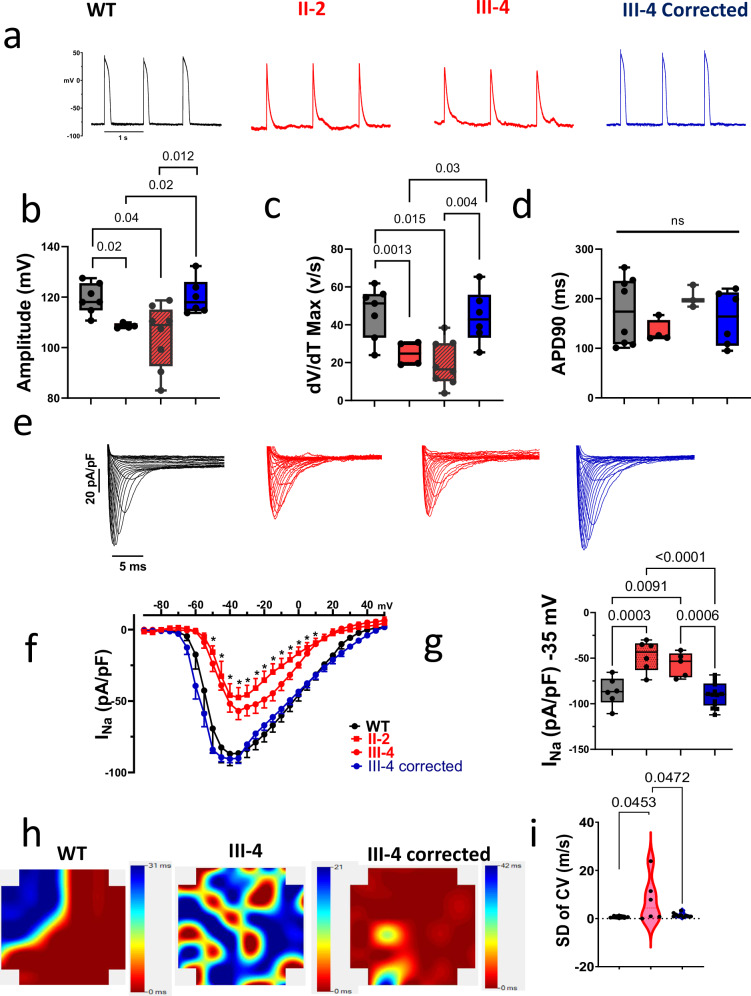
Reduced sodium current underlies electrophysiological alterations in *LMNA* p.S143P iPSC-aCMs. **a** Representative evoked AP traces. **b**–**d** Reduced AP amplitude and (**c**) upstroke velocity (dv/dt max), an indicator of atrial conduction in *LMNA* p.S143P iPSC-aCMs with (**d**) no change in APD90. (**b**–**c** WT, *n* = 7. II-2, *n* = 4, III-4,*n* = 8, III-4 corrected *n* = 6 iPSC-aCMs. **d** WT, *n* = 8. II-2, *n* = 4, III-4,*n* = 3, III-4 corrected *n* = 6 iPSC-aCMs The box plot extends from the 25th to the 75th percentile. The line in the middle represents the median. The whiskers go down to the min and max points)**. e** Representative sodium current (*I*_Na_) traces. **f**, **g** Current-voltage (I/V) curve showing reduced *I*_*Na*_ in *LMNA* p.S143P iPSC-aCMs are quantified in a bar graph. (**f**, **g**:WT, *n* = 6. II-2, *n* = 6, III-4,*n* = 5, III-4 corrected *n* = 15 iPSC-aCMs. Ordinary one-way ANOVA with multiple comparisons, significance at *p *< 0.05. Adjusted *p* values: WT vs. III-4 < 0.0003, WT vs. II-2 = 0.0091, III-4 vs. III-4 Corrected= 0.0006, II-2 vs. III-4 Corrected <0.0001). **h**, **i** Activation maps of propagation of electrical activity measured by an MEA show marked conduction heterogeneity in *LMNA* p.S143P iPSC-aCMs. **h**, **i** Increased standard deviation (SD) of conduction velocity (CV) measurements in *LMNA* p.S143P iPSC-aCMs. aCMs (*n* = 6 stimulated CV measurements for each group). (Statisitical comparisons for b-d,g and i done using ordinary one-way ANOVA with multiple comparisons,. Significance at *p* < 0.05). **MEA**-multi electrode array.

To assess the EP profile of matured *LMNA* p.S143P iPSC-aCMs^39–41^, we measured evoked action potentials (**AP**) in isolated mature iPSC-aCMs. *LMNA* p.S143P iPSC-aCMs exhibited altered AP profiles, including reduced AP amplitude and upstroke velocity (dV/dTmax), a key indicator of atrial conduction, while AP duration at 90% repolarization (**APD90**) remained unchanged (Fig. 7a–d). AP amplitude and upstroke velocity are primarily determined by the peak *I*_Na_, conducted via the main cardiac sodium channel Nav1.5, encoded by *SCN5A*. Consistently, voltage-clamp recordings revealed a significant reduction in peak *I*_Na_ density in *LMNA* p.S143P iPSC-aCMs compared to WT and *LMNA* p.S143P-CRISPR-corrected cells (Fig. 7e, f). Stimulated multielectrode array recordings further demonstrated marked conduction heterogeneity across *LMNA* p.S143P iPSC-aCM monolayers, a pattern absent in WT and corrected iPSCs (Fig. 7g). We also observed decreased expression of *SCN5A* in *LMNA* p.S143P iPSC-aCMs (Fig. 4c, and Supplementary Fig. 8). Importantly, potassium (*I*_K_) and calcium (*I*_Ca_) currents, which regulate the repolarization phase of the AP, remained unchanged, reinforcing that the observed EP changes in *LMNA* p.S143P iPSC-aCMs are explicitly driven by alterations in *I*_Na_ (Supplementary Fig. 9). In summary, reduced conduction velocity due to *SCN5A* downregulation in *LMNA* p.S143P iPSC-aCMs promotes re-entry, establishing an arrhythmogenic substrate for early-onset AF.

### Altered calcium handling and contractility in* LMNA* p.S143P iPSC-aCMs

Calcium transient measurements revealed that *LMNA* p.S143P iPSC-aCMs exhibited reduced transient amplitude and altered decay times compared to WT and isogenic controls. While the decay time was significantly shorter in (III-4), it seemed to be much more prolonged in iPSC-aCMs derived from II-2 (Supplementary Fig. 10a–c). Contractility assessments further demonstrated a decrease in contractile amplitude in *LMNA* p.S143P iPSC-aCMs compared to controls (Supplementary Fig. 10d–f). These disruptions in calcium handling and contractility are consistent with the electromechanical dysfunction underlying atrial myopathy and AF^42^.

### Downregulation of *SCN5A* and *TBX5* in* LMNA*-G449V iPSC-aCMs

**Fig. 8:**
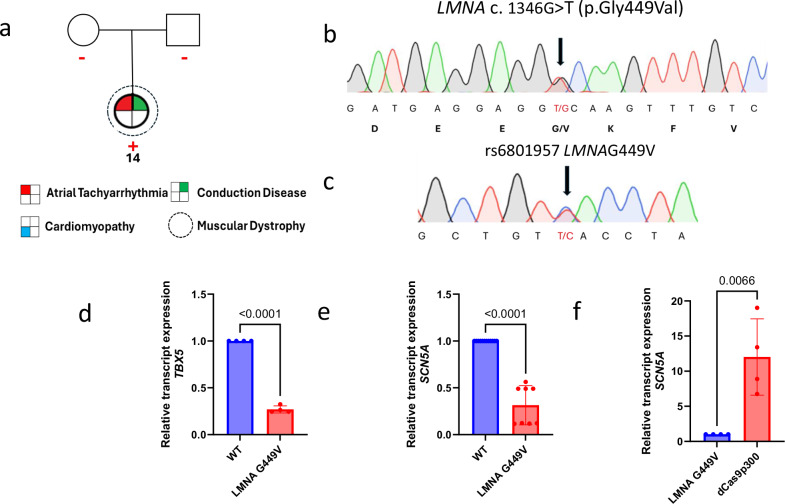
Sodium channel dysfunction is recapitulated in the *LMNA-*G449V variant. **a** Pedigree of proband harboring the *LMNA-*G449V variant. **b** Sanger sequencing confirms a heterozygous G > T variant. **c** Presence of one risk allele at the rs6801957 SNP. **d** Reduction in transcript levels of *TBX5* and **e** *SCN5A*. **f** CRISPRa o**f** the *SCN10A* enhancer increases *SCN5A* expression. **d**, **f** *n* = 4, e, *n *= 6 biological replicates for each group; statistical analysis done using unpaired t-test with Welch’s correction. Significance at *p* < 0.05. *P* values: d. *TBX5* < 0.0001, **e** *SCN5A* < 0.0001 **f** *SCN5A* = 0.0066 Data are presented as mean values +/- SD).

To determine whether additional *LMNA* variants share similar molecular mechanisms underlying AF susceptibility, we generated iPSC-aCMs from an individual harboring a de novo, heterozygous, pathogenic variant, *LMNA* c.1346 G > T p.Gly449Val, associated with cardiac disease and muscular dystrophy (Fig. 8a–c). Her cardiac phenotype included recurrent atrial tachycardia, premature atrial contractions, sinus pauses, and intermittent absence of atrial electrical activity on the EKG. Echocardiography demonstrated mild bi-atrial dilation by echo and mild mitral annular dilation with preserved left ventricular ejection fraction (61-67%) in the absence of guideline-directed medical therapy. Consistent with findings in *LMNA* p.S143P iPSC-aCMs, we observed downregulation of *TBX5* and *SCN5A* (Fig. 8d–f). Genotyping confirmed heterozygosity for the AF-associated SNP rs6801957. CRISPRa of the corresponding *SCN10A* intronic enhancer increased *SCN5A* expression, supporting a conserved regulatory interaction at this locus.

## Discussion

Large-scale exome-wide genetic studies have linked rare *LMNA* variants to early-onset AF^5^. However, the phenotypic expression and severity of AF among carriers remain highly variable. While pathogenic *LMNA* variants associated with DCM exhibit high penetrance, the final clinical phenotype is influenced by gene-gene interactions, contributing to the heritability of complex diseases like AF. Although a PRS quantifies the cumulative impact of common variants, it does not fully capture epistatic interactions, which may be non-additive and context-dependent^43–45^.

We applied an AF PRS in two large population cohorts, the UK Biobank and AoU. Carriers of rare *LMNA* variants with a high PRS had a two-fold increased risk of early-onset AF compared to those with a lower PRS, particularly in individuals under 65 years old. While prior studies suggest that common variants influence the penetrance of rare monogenic variants, our findings provide direct molecular evidence supporting this interaction^6,8^. Our findings suggest that *LMNA* PAVs and polygenic risk contribute independently to AF risk in an additive manner. Even in the absence of synergistic interaction, their combined effect can increase absolute risk, supporting a more comprehensive approach to precision medicine and targeted screening strategies. Clinically, incorporating both PRS and rare-variant status could improve AF risk stratification by identifying individuals at high risk, such as those with a pathogenic *LMNA* variant and a high PRS, who might benefit from earlier monitoring or preventive strategies.

To elucidate the molecular mechanisms by which rare and common variants act in concert to increase AF risk, we integrated gene expression, chromatin accessibility, and PC-Hi-C interaction data and identified dysregulated regulatory networks at key AF susceptibility loci. These findings reveal the molecular mechanism by which rare and common variant epistatic interactions alter gene regulatory networks that contribute to arrhythmogenesis. Specifically, we observed chromatin accessibility changes at AF-associated loci, including a variant-sensitive *SCN10A* enhancer. These alterations reduced *SCN5A* expression, diminished *I*_Na_, and conduction abnormalities, creating a substrate for re-entrant AF.

Most AF risk loci identified through GWAS reside in non-coding regions that regulate gene expression. We observed altered chromatin accessibility at several AF-associated loci and their target genes. While a SNP within the *SCN10A* intronic region had the strongest effect, we also identified associations with *NPTN*, a structural gene involved in atrial differentiation and heart rate regulation; and *ZFHX3, a* TF that plays a key role in atrial homeostais^34,36,46^ and *WDR1*, which is involved in actin myofibril disassembly^47^. Although *SCN5A* downregulation emerged as the primary driver of arrhythmic risk, our findings suggest that AF results from broader disruptions involving ion and non-ion channel proteins, including regulatory enzymes and structural components.

To establish a causal link between chromatin remodeling and AF risk and to interrogate the function of variant regulatory regions, we employed CRISPR epigenetic assays to restore chromatin accessibility. Our approach validated a previously known *SCN10A* intronic enhancer in a region strongly associated with AF and electrocardiographic traits^2,15,48^, demonstrating that this common polymorphism contributes to sodium channel dysfunction underlying early-onset AF in *LMNA* variant carriers. Importantly, we found a concordant regulatory effect of the SNP rs6801957 on SCN5A expression in three individuals: the proband, her mother, and an additional *LMNA* variant carrier. In addition to the S143P variant, we extended our analysis to iPSC-aCMs derived from a distinct variant, *LMNA* G449V, which also showed a downregulation of *TBX5* and *SCN5A*, demonstrating that transcriptional dysregulation and sodium channel dysfunction underlie atrial pathology induced by *LMNA* variants. Collectively, these observations support the possibility that dysregulation of atrial transcriptional networks and sodium channel expression is not confined to the S143P variant and may occur across multiple pathogenic *LMNA* genotypes. Interestingly, type I Brugada-like EKG patterns have been noted in *LMNA* variant carriers following cardiac arrest, a condition commonly associated with impaired sodium channel activity^49^. Our study provides a mechanistic explanation for this association and supports the inclusion of *LMNA* in genetic testing panels for Brugada syndrome.

While the *SCN10A* enhancer serves as a mechanistically validated example among a broader set of loci, this study also provides insights into the regulatory function of additional variant regions and their target genes, including an enhancer for *ZFHX3* and *WDR1*. These findings advance our understanding of the genetic architecture of AF and demonstrate the interplay of ion-channel and structural remodeling underlying the pathogenesis of AF in Lamin A/C Cardiomyopathy.

In *LMNA* p.S143P iPSC-aCMs, we observed coordinated molecular and functional changes that together create an arrhythmogenic substrate supporting both atrial reentry and ectopic activity. Downregulation of sarcomeric and desmosomal genes associated with early-onset AF (*MYL4, TTN, MYBPC3, DSP*, and *PKP2*) is predicted not only to weaken atrial structural integrity but also to increase conduction heterogeneity, favoring reentry^50^. At the same time, enrichment of dilated cardiomyopathy and β-adrenergic signaling pathways is consistent with maladaptive remodeling and calcium-dependent triggered activity. Suppression of cardiac hypertrophy and differentiation programs suggests impaired atrial maturation and reduced conduction reserve, and activation of DNA damage repair pathways reflects chronic cellular stress that may further promote fibrosis and conduction slowing. Reduced chromatin accessibility at enhancers modulating key atrial TFs (*TBX5, GATA4, GATA6*) and *NPPA* likely propagates widespread transcriptional dysregulation of downstream effector genes, including *SCN5A*, thereby impairing *I*_Na_ and slowing atrial conduction. Additional changes, such as upregulation of *CEP68* and downregulation of *NPTN* and *SGK1*, may further disrupt cytoskeletal organization, ion channel regulation, and calcium handling, consistent with the observed reductions in calcium transient amplitude and duration and increased irregularity. While multiple pathways contribute to arrhythmogenesis, impaired atrial conduction driven in part by chromatin-mediated disruption of TF networks, particularly TBX5 and its downstream sodium channel targets, emerges as a dominant mechanism, linking *LMNA*-mediated chromatin remodeling to atrial myopathy and AF.

The findings of this study have several important implications. Our results highlight the significance of chromatin remodeling at multiple AF-associated loci, thereby disrupting the atrial gene regulatory networks underlying AF. This underscores the complex etiology of AF, the need to better understand its molecular substrate, and the need to expand therapeutic strategies beyond traditional ion channel-targeting drugs. Additionally, our successful CRISPR-based epigenetic assays demonstrate the potential to develop high-throughput assays that screen large numbers of variants, thereby keeping genetic discoveries and their functional interpretation aligned. Ultimately, our findings underscore the need for enhanced genetic risk prediction models for AF and other complex diseases. Such models should integrate the cumulative effects of common variants with small effect sizes alongside highly penetrant rare variants while accounting for epistatic interactions.

## Methods

### Recruitment of participants

We used the University of Illinois Chicago’s Institutional Review Board–approved protocol to enroll participants after receiving informed written consent.

### Generation of human iPSCs and differentiation to atrial cardiomyocyte iPSC-aCMs

Human iPSCs were derived from reprogrammed peripheral blood mononuclear cells (PBMCs) from the proband and unaffected controls, as previously described^51^, Briefly, PBMCs were isolated from whole blood by Ficoll separation method and cultured in PBMC medium for 48hrs. After 48 hrs,PBMCs were transduced with reprogramming sendai viruses and maintained for three weeks. Around day 21 iPSC colonies were identified, isolated and subcultured. iPSCs were differentiated using the STEMdiff atrial cardiomyocyte differentiation kit. The atrial cardiomyocyte population was purified through glucose starvation and lactate replacement, resulting in contracting monolayers of iPSC-aCMs. Human iPSC-aCMs were then matured using the maturation protocol following dissociation and replating on fibronectin-coated plates and maintained in Cardiomyocyte Maintenance Media supplemented with T3, insulin-like growth factor-1, and dexamethasone as previously described^39,41^. Our protocol typically yields ~80 to 90% pure iPSC-aCMs and fibroblasts based on immunostaining analysis^39,41,51^.

### Generation of isogenic control from LMNA-S143P by CRISPR correction

The isogenic control (LMNA-S143P Corrected) was generated using the Alt-R CRISPR-Cas9, homology-directed repair (HDR) system.^39^. Single guide (**sg**)RNAs and HDR oligos were nucleofected in *LMNA*-S143P hiPSCs. The sgRNAs were designed using the IDT CRISPR design tool based on an algorithm predicting optimal off-target and on-target scores. sgRNA 5’GGAGGCTCTGCTGAACCCCA-3’ was used for genetic correction of Pro143 to Ser, C to T in exon 2. Single-cell clones were isolated, expanded, and verified by next-generation sequencing, as well as Sanger sequencing.

### Electrophysiology

Whole-cell -patch-clamp recordings on single-cell iPSC-aCMs to measure INa, ICa, L, and IK were performed according to previously published protocols using the Axopatch 200B amplifier controlled by pClamp10 software through an Axon Digidata 1440A^39,41,51^. Sodium current measurements were done in a voltage clamp setup; pipettes were filled in mM: 135 CsF, 5 NaCl,5 MgATP, 10 Hepes, 10 EGTA, pH 7.2 with CsOH. Cells were perfused with a solution in mM: 85 CsCl, 10 glucose, 1 MgCl2, 1 CaCl2, 20 Hepes, 50 NaCl, pH 7.4 with CsOH.; 10 uM Nifedipine was added to block calcium channel. Sodium current was elicited by a series of 600 ms test potentials varying from -90 to -50 mV in 5 mV increments from a holding potential of -120 mV.

L-type calcium current was obtained in the whole cell voltage clamp configuration. External recording solution contained (in mM) 136 TEA-Cl, 2 CaCl2, 1.8 MgCl2, 10 HEPES, 5 4-aminopyridine, and 10 glucose (pH 7.4 with TEA-OH). The pipette solution contained (in mM): 125 CsCl, 20- TEA-Cl, 10 EGTA, 10 HEPES, 5 phosphocreatine, 5 Mg2ATP (pH 7.2 with CsOH). IPSC-aCM were held at -80 mV, and 10 mV depolarizing steps from -50 mV to + 50 mV for 300 ms were applied. Recording solutions allowed for the specific measurement of the calcium current in the absence of the contaminating Na and K currents.

Potassium current was measured in a whole cell rupture voltage clamp with patch clamp pipettes filled with internal solution in mM: 100 K-aspartate, 20 KCl, 5 Mg ATP, 2 MgCl2, 10 HEPES, 5 EGTA pH 7.2 with KOH; External solution was normal tyrode solution in mM: 140 NaCl, 4 KCl, 1.2 MgCl2, 1.8 CaCl2, 10 glucose, 10 HEPES pH 7.4 with NaOH; 10 uM Nifedipine was added to block calcium channels. IK outward current was measured from a holding potential of -60 mV to test potentials of –30 mV to +50 mV with 20 mV increments for 3 seconds duration.

### Calcium transient recordings and contractility measurements

The Ionoptix system was used for contractility and calcium transient measurements. Contracting monolayers of iPSC-aCMs were incubated in 1uM Fura 2 AM calcium binding dye dissolved in Tyrode’s solution. After 20 min of incubation, the dye solution was removed and replaced with Tyrode’s solution. Calcium transients were analyzed using the ION-OPTIX analysis software. The software performs background detection and generates traces after subtraction of the background. Cytomotion, a label-free detection system, was used for contractility analysis, which provides high-speed data acquisition at 1000 Hz. Inotropic differences were measured by differences in contractile amplitude. Contractility and calcium transient analysis by the ION wizard software is done according to an algorithm optimized for iPSC-derived cardiomyocytes.

### Conduction velocity recordings

iPSC-aCMs from the three groups were plated on dishes with multi-electrode arrays. Biphasic stimuli were applied to iPSC-aCMs at an amplitude of 1000 mV for a duration of 800us for 15 cycles. CV across the arrays was measured using the MEA 2100 at each stimulus. Conduction velocities were generated from the software based on the time of stimulus application to measure electrical impulse across the array using Cardio 2D software.

### CRISPR activation/ inhibition

Tracr RNAs targeting the *SCN5A* promoter and *SCN10A* intronic enhancer were cloned into backbones with sgRNA scaffold (addgene#47108). iPSC-aCMs were co-transfected with individual plasmids with sgRNAs, dCas9- VP64-p65-Rta (addgene#63798) or dCas9 CBP/ p300 (addgene # 61357) and a plasmid with a GFP expression cassette (addgene#40973). dCas9- VP64-p65-Rta was used for activation of SCN5A promoter while dCas9 CBP/ p300 was used for SCN10A enhancer activation. All transfections were done using Lipofectamine 3000 according to the manufacturer’s protocol. 48 hrs post-transfection, GFP-positive iPSC-aCMs were selected for sodium current measurements. For CRISPR inhibition, a plasmid encoding dCas9KRAB-MECP2 (Addgene Plasmid #110821) was co-transfected with sgRNAs targeting the variant region. sgRNAs sequences are in Supplementary Data 1. Synthetic sgRNAs were transfected using the RNAi Max Transfection Kit at least 48 h after transfection of CRISPRa/I/plasmid. Transfection efficiency was measured by GFP fluorescence in live cell cultures and gene expression changes by qPCR.

### RNA sequencing, ATAC sequencing, and bioinformatic analysis

RNA extraction and library preparation on triplicate samples of ~1 million cells was done by the University of Chicago genome sequencing core. RNA was isolated using Trizol and QC-ed by bioAnalyzer (Agilent 2100); RNA-seq libraries were generated using an Illumina TruSEQ-like protocol (provided by Illumina). Libraries were QC-ed by BioAnalyzer and sequenced on the Illumina NovaSEQ-X using reagents and protocols provided by Illumina.

### RNA-seq data processing and differential expression analysis

Raw paired-end RNA-seq reads were aligned to the Homo sapiens GRCh38 reference genome using STAR (v2.7.9a)^52^ with default two-pass settings and the NCBI RefSeq annotation for GRCh38, sourced from the Illumina iGenomes archive. Gene-level read counts were obtained from STAR’s --quantMode GeneCounts output and merged into a unified count matrix. Differential expression analysis was performed in R using the DESeq2 package^53^. Counts were normalized with the median-of-ratios method, and dispersion estimates were fitted using DESeq2’s empirical Bayes procedure. Log₂ fold changes were shrunken using the ashr method^54^ to stabilize estimates for low-count genes. Genes with adjusted *P* < 0.05 (Benjamini–Hochberg) were considered significantly differentially expressed. Strand-specific bigWig coverage files were generated from aligned BAMs using deepTools (bamCoverage) for genome browser visualization^55^. Gene enrichment analysis was performed using ShinyGO 0.81 and Ingenuity Pathway Analysis (IPA). IPA was also used for upstream regulator and transcription factor analysis.

For ATAC-seq, Triplicate samples of ~500 K single dissociated live iPSC-aCMs were provided to the Genomic Sequencing Core at Northwestern University for Library preparation. Briefly, the Cell membrane was permeabilized with nuclear lysis buffer (10 mM tris, 10 mM NaCl, 3 mM MgCl2, and 0.5% IGEPAL-630). Then, cells were resuspended in 50uL of transposase reaction mixture (22.5uL nuclease-free water, 25uL of TD buffer, and 2.5uL of TDE1 enzyme, Illumina #20034197). Transposition was carried out at 37 °C for 22 min, followed by DNA purification with DNA Clean and Concentrator-5 (Zymo Research) according to the manufacturer’s recommendation. Following purification, library fragments were PCR amplified with Nextera XT v2 adapter primers. Multiplexed and pooled library was sequenced on the Novaseq X Plus with paired ends of 50 nucleotides per the manufacturer’s instructions. The library preparation and sequencing were done at Northwestern University NUSeq facility core. The raw data was analyzed by the University of Illinois research informatics core. Shiny GO V0.85 was used for gene enrichment analysis. Heatmaps were generated using Morpheus by Broad Institute.

### ATAC-seq analysis

#### Peak calling

Read alignments were first adjusted to account for TAC transposon binding: +4 bp for + strand alignments, -5 bp for - strand alignments. The open chromatin enrichment track was generated by first creating a bedGraph from the raw reads using bedtools genomcov^56^, then converted to bigWig using the UCSC tool bedGraphToBigWig^57^. Tracks were normalized by the sum of alignment lengths of over 1 billion. The start position track was generated by taking just the first base of the alignment for + strand alignments or the last base of the alignment for - strand alignments, then creating bedGraph and bigWig tracks as for the open chromatin; tracks were normalized to the alignment count over 1 million. Open chromatin peaks were called using MACS2^58^ with -nomodel set and no background provided; peaks with a score >5 were retained.

### Nucleosome positioning

Properly paired read pairs were first put into nucleosome-free or nucleosome-containing bins based on their fragment size. Fragments ≤100 bp were considered nucleosome-free (background) and converted to a single read covering the length of the fragment. Fragments 180–247 bp were considered single-nucleosome-containing and converted to a single read the length of the fragment; similarly, fragments 315–473 bp or 558–615 bp were considered two- or three-nucleosome containing and split into 2 or 3 reads covering half or 1/3 of the total fragment length. Single-, two-, and three-nucleosome reads were combined into the nucleosome signal read set. Nucleosome positioning analysis was run using danpos^59^ with command dpos and parameters -m 1 -a 10 -jd 20 -clonalcut 0, and contrasting nucleosome signal to nucleosome background for each sample. Wig tracks from DanPos were re-normalized to counts per billion bases using the sum of alignment lengths over 1 billion from the original BAM file and converted to bigWig using the UCSC tool wigToBigWig^57^.

### Differential analysis of detected peaks

Differential analysis of quantitated peaks compared to genotype was performed using the software package edgeR on raw peak counts^60^ Before analysis, the data were filtered to remove any peaks that had less than 20 total counts summed across all samples. Data were normalized as counts per million, and an additional normalization factor was computed using the TMM algorithm. Statistical tests were performed using the “exactTest” function in edgeR. Adjusted *p* values (*q* values) were calculated using the Benjamini-Hochberg false discovery rate (FDR) correction^58^. Significantly enriched peaks were determined based on an FDR threshold of 5% (0.05).

### Motif analysis of differentially enriched peaks

First, searches were performed for instances of known transcription factor motifs in all peak sequences from the JASPAR database^61^ using FIMO^62^. Then, using Fisher’s Exact Test, motif enrichment statistics were computed for each set of DE peaks by comparing the fraction of motif-containing peaks within or not within the DE peaks. Adjusted *p* values (*q* values) were calculated using the Benjamini-Hochberg false discovery rate (FDR) correction. (Benjamini and Hochberg, 1995)^63^ Significantly enriched motifs were determined based on an FDR threshold of 5% (0.05).

### RT-PCR

Total RNA was isolated from iPSC-aCMs using a Qiagen RNA extraction kit. Reverse transcription to synthesize cDNA was conducted using SuperScript III Reverse Transcriptase (Thermo Fisher Scientific). For the qPCR analysis, specific assays and primers were selected for target genes with glyceraldehyde 3-phosphate dehydrogenase (GAPDH) as the normalization reference gene. qPCR reactions were performed on an ABI QuantStudio 5 system (Applied Biosystems), using SYBR Green PCR Master Mix to accurately detect and quantify PCR amplification products. Relative expression levels of the target genes were calculated using the ΔΔCt method by quantifying gene expression changes in the experimental samples relative to the control. For the ΔCt calculation, the cycling time (Ct) value of the target gene was subtracted from the Ct value of GAPDH in the same sample using ΔCt = Ct_target gene_ − Ct_reference gene_. The ΔΔCt value was then calculated using ΔΔCt = ΔCt_experimental_ *–* ΔCT_control_. The gene’s relative expression was calculated using relative gene expression = 2^−ΔΔCt^. **Primer pair for** ***SCN5A*- Forward- TCTCTATGGCAATCCACCCCA, Reverse- GAGGACATACAAGGCGTTGGT**. The qPCR primers are provided in Table 2.

**Table 2:** Primers for qPCR

	Forward	reverse
*SCN5A*	TCTCTATGGCAATCCACCCCA	GAGGACATACAAGGCGTTGGT
*ZFHX3*	CAAGTTCACGACGGACAACCT	GCTTGCACTGGTATGAGTCCC
*CEP68*	GGGTAGACCTGGATAGCTTCTC	CAACGAGCATCCCTACTGCC
*SCN10A short*	GCAGCTTCTTCTGGGTCAATG	TACACGTTGTCCTCCCACTTG
*GATA4*	CGACACCCCAATCTCGATATG	GTTGCACAGATAGTGACCCGT
*GATA6*	CTCAGTTCCTACGCTTCGCAT	GTCGAGGTCAGTGAACAGCA
*NPPA*	CAACGCAGACCTGATGGATTT	AGCCCCCGCTTCTTCATTC
*TBX5*	CTGTGGCTAAAATTCCACGAAGT	GTGATCGTCGGCAGGTACAAT
*BMP10*	GCTATCCAGGCCCAAGTTGT	GAATGAAGATCTGTTTTCCCAGCC
*SGK1*	AGG ATG GGT CTG AAC GAC TTT	GCC CTT TCC GAT CAC TTT CAA G
*ALDH8A1*	GCC CTT TCC GAT CAC TTT CAA G	CCC TGT TGA TGG GTC GTA AGA A
*SLC2A9*	CCT CTA CGG CTA CAA CCT GTC	AGA GTG TCT GGG TCT ATT GGA C
*WDR1*	CAC AGC CGC TTT GTC AAC TG	ACT AAT TGC GTA AAT CCC ACC G
*ZMIZ1*	TGT TTG ACG GTG GTC AGT CG	CTT GTC TCG GTT TGC AGC AC

Primers for measuring gene expression by qPCR are listed in Table 2.

### Western blotting

Protein lysates from iPSC-aCMs were isolated using 1× RIPA buffer. Each sample containing 50 μg of protein was subjected to SDS-PAGE gel electrophoresis. The resolved gels were then electro-transferred onto 0.2 μm PVDF membranes. After a 2-hour blocking step with 5% BSA, membranes were probed with specific antibodies for target proteins. Blots were developed using either anti-rabbit HRP or anti-mouse HRP and scanned with C280 imaging systems (Azure Biosystems). ImageJ software was used to determine protein expression levels. Antibody used: SCN5A- 68273-1-Ig SCN5A Mouse McAb from protein tech, Proteintech TBX4/5 Cat No. 13178-1-AP, Proteintech SCN5ACat No. 23016-1-AP, Lamin A/C sc-376248, Proteintech Lamin A/C 10298-1-AP. Antibody validation provided by supplier. Supplier recommended antibody dilutions were used.

### Immunofluorescence

iPSC-aCMs were washed with PBS, fixed with 4% paraformaldehyde at 37 °C for 10 min, 0.1% Triton X-100 was used for permeabilization for 15 min, and blocked with 10% BSA for 1 h. Primary antibody was diluted at 1:200 in 10% BSA and incubated in 10% BSA at 4 °C overnight. The secondary antibody was incubated in 1:1000 dilution in 10% BSA for 1 h. Primary antibodies utilized were rabbit polyclonal anti-cTnT (Abcam; ab45932) and mouse anti-sarcomeric anti–α-actinin (Abcam; EA-53 ab9465). Secondary antibodies used were goat anti-rabbit Alexa Fluor 488 (Abcam; ab150077) and goat anti-mouse Alexa Fluor 594 (Abcam; 150116). Nuclei were stained using DAPI (Thermo Fisher Scientific). The cells were visualized using a Zeiss Laser Scanning confocal microscope (LSM 710) (META) objective and analyzed on ImageJ (NIH).

### Transmission electron microscopy

hiPSC-aCMs were fixed in Warmed (37 °C) 2.5% glutaraldehyde in 0.1 M Sorenson’s Buffer for 60 min at room temperature, followed by gently scraping cells into a microcentrifuge tube prefilled with 2.5% glutaraldehyde in 0.1 M Sorenson’s buffer. Samples were centrifuged at 2500 g for 10 min at room temperature, and the pellet was dislodged and flipped with a hypodermic needle to ensure the fixative solution penetrated the entire sample. The pellet was kept at room temperature to fix for an additional 60 min. The 2.5% glutaraldehyde in 0.1 M Sorenson’s buffer solution was removed and replaced with 1% glutaraldehyde +4% paraformaldehyde in 0.1 M Sorenson’s buffer and stored at 4 °C.

### Statistics & reproducibility

All experiments were done in a minimum of three groups and repeated across at least three independent differentiations. Data was presented as mean ± SD. For data with normal distribution, nonparametric unpaired and 2-tailed t-test was used to determine statistical significance between 2 groups and 1-way ANOVA with multiple comparisons or multiple t-tests were used. *P* < 0.05 was considered significant. No data was excluded from the analysis unless determined as outliers.

### UK Biobank and AoU PRS methods

The UK Biobank standard AF PRS (data field 26212) derived by Genomics PLC from external data sources was used^64^. The PRS for 420,196 participants was classified into 3 categories of genetic risk (high-risk [bottom quintile], high-risk [top quintile] and medium-risk [middle quintiles]. Logistic regression was used to assess the association of AF with PRS. Cox regression was used to calculate the hazard ratio (HR) for AF across PRS categories adjusting for the first 10 genetic PCs, sex, age and hypertension. Time to AF diagnosis was visualized with Kaplan Meier curves. Analyses were performed on the UK Biobank research analysis platform (application number 47602).

All of Us (AoU) Controlled Tier Dataset, version 7 was utilized in this study. AF cases were identified using OMOP Concept IDs: 313217, 321319, 3656119, 4064452, 4071896, 4108822, 4108832, 4117112, 4124693, 4141360, 4154290, 4163710, 4178584, 4199501, 4232495, 4232691, 4232697, 42872390, 44782442, 44783568, and 45768480. Whole-genome sequencing data from AoU were extracted and subjected to quality control using PLINK v1.9. This involved filtering out SNPs with a minor allele frequency (MAF) of <0.01 and excluding SNPs with a genotyping rate <95%.

SNP pruning was performed using PLINK with a 50 kb sliding window, shifted by 5 SNPs at each step, and an r² threshold of 0.2. The pruned SNPs were then used to compute genetic principal components (PCs) via PLINK. For Polygenic Risk Score (PRS) calculation, GWAS summary statistics for AF were obtained from DOI: 10.5281/zenodo.6631951, and genomic coordinates were lifted over from hg19 to hg38 using the pyliftover tool (https://github.com/konstantint/pyliftover)

The PRS was classified into three categories of genetic risk: low-risk (bottom quintile), medium-risk (middle quintiles), and high-risk (top quintile). Survival analysis was conducted using Cox Proportional Hazards modeling, with age at diagnosis serving as the time-to-event variable.

### Primers for sequencing AF variant regions

Primers for sequencing AF variant regions are provided in Table 3.

**Table 3:** Primers for Sequencing

	forward	reverse
rs7172038	TCAGAGCCAGGCCAGAGAGG	TTTCTACCACCGCACCCAGC
rs4896104	TGGCCTCCTGAGAATGTCCAGA	CCACACGTGTCAGACTGGGC
rs2359171	ACACACACCTCAGAGGCCCA	AACTGGCCACTCGGTCCTTG
rs1886512	AGCAGCCAGCTTGGCTTACC	GGGCAAGAACTCCTCCAGGG
rs12640611	GTCTTCCCCAACCCCAAGGC	TTGCCTAGCCATCTGCAGCC
rs2540949	CGCCCAGATGCGGAGAGAAA	TTGGACATTCGTCCCCAGGC
rs1769758	AGGAGGGGCAAGGGAGTGAG	GGGAGAAGCTGTCGGGGGTA
rs6801957	GGGAACTTGGCCACCTG	TTGAGTCTGTAGCTCTCCCATA
LMNA S143P	AAGAAGGAGGGTGACCTGATAG	GCCTAGGTAGAAGAGTGAGTGT

## Supplementary information

Supplementary Information

Description of Additional Supplementary Files

Supplementary Data 1

Transparent Peer Review file

## Source data

Source Data

Supplementary Source Data

## Data Availability

The RNA and ATAC sequencing data are publicly available on the Gene Expression Omnibus, under the accession numbers GSE294948 and GSE295056. Source data are available in the source data file. Source data are provided with this paper.
