# Supplementary material for: Gene-gene interactions between a LMNA variant and common polymorphisms drive early-onset atrial fibrillation
Source: Nat Commun. 2026 May 19;17:6594. doi: 10.1038/s41467-026-73113-0 (PMC13381584; doi:10.1038/s41467-026-73113-0)
Supplement: Supplementary file 1 — Supplementary Information [file 41467_2026_73113_MOESM1_ESM.pdf]

SUPPLEMENTARY INFORMATION

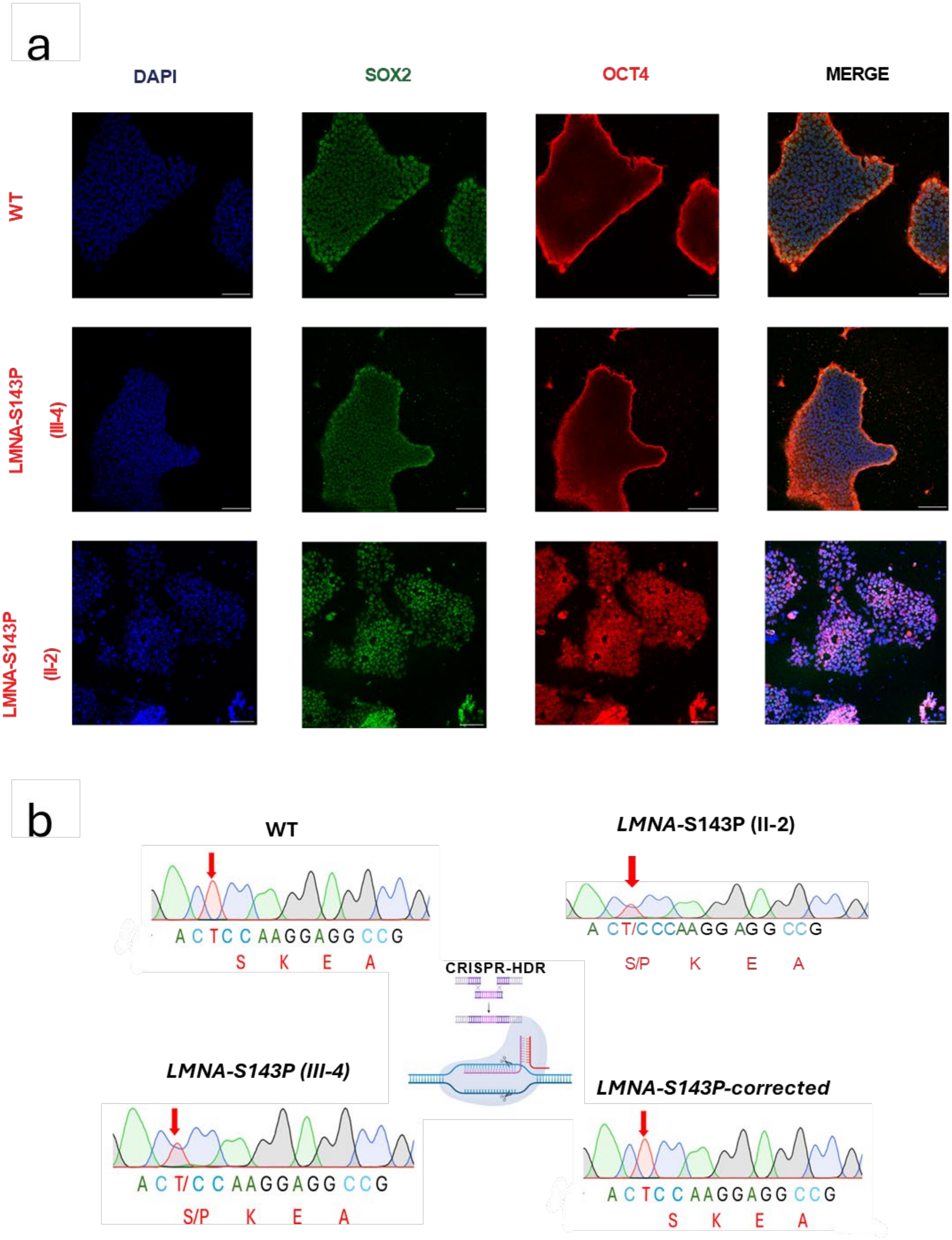

Supplementary Figure 1: **Generation and characterization of WT, *LMNA* p.S143P, and S143P-Corrected iPSCs**. **a.** Immunostaining of pluripotency markers OCT4 and SOX2 in iPSCs. **b.** Sanger sequencing reveals a heterozygous missense variant at position 427 T>C, resulting in an amino acid change from Serine to Proline. The variant is corrected back to WT using CRISPR and homology-directed repair. Scale bar at 110um. Created in BioRender. Owais, A. (2026) https://BioRender.com/xntq64i

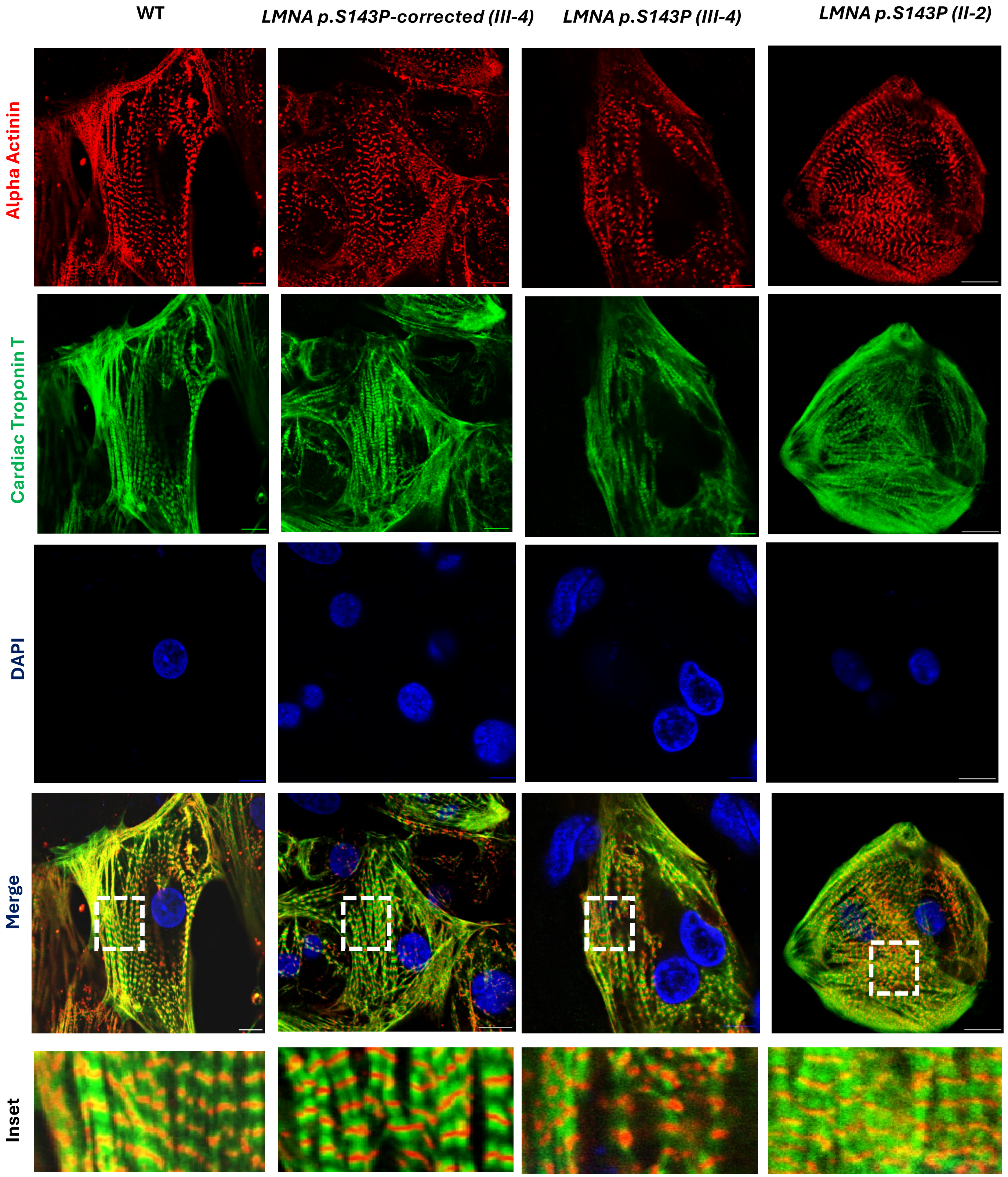
WT
LMNA p.S143P-corrected (III-4)
LMNA p.S143P (III-4)
LMNA p.S143P (II-2)
Alpha Actinin
Cardiac Troponin T
DAPI
Merge
Inset

Supplementary Figure 2: **Immunostaining iPSC-aCMs for pan cardiac markers, cTnT, and Alpha Actinin**. Scale bars 13um.

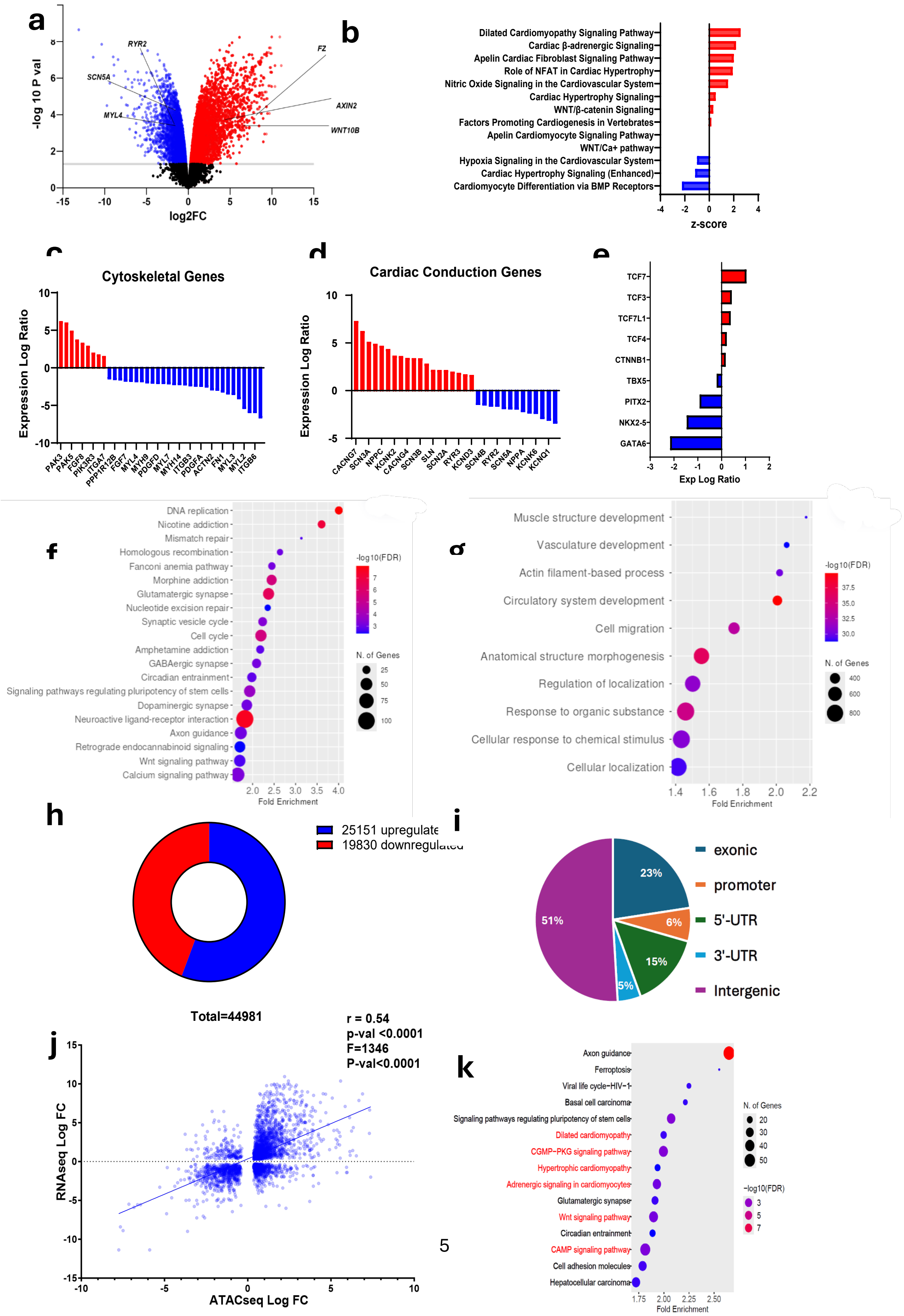
a
-log 10 P val
log2FC
RYR2
SCN5A
MYL4
FZ
AXIN2
WNT10B
b
Dilated Cardiomyopathy Signaling Pathway
Cardiac β-adrenergic Signaling
Apelin Cardiac Fibroblast Signaling Pathway
Role of NFAT in Cardiac Hypertrophy
Nitric Oxide Signaling in the Cardiovascular System
Cardiac Hypertrophy Signaling
WNT/β-catenin Signaling
Factors Promoting Cardiogenesis in Vertebrates
Apelin Cardiomyocyte Signaling Pathway
WNT/Ca+ pathway
Hypoxia Signaling in the Cardiovascular System
Cardiac Hypertrophy Signaling (Enhanced)
Cardiomyocyte Differentiation via BMP Receptors
z-score
Cytoskeletal Genes
Expression Log Ratio
PAK3
PAK5
FGF8
PIK3R3
ITGA7
PPP1R12B
FGF7
MYL4
MYH9
PDGFD
MYL7
MYH14
ITGB3
PDGFA
ACTN2
FN1
MYL3
MYL2
ITGB6
Cardiac Conduction Genes
Expression Log Ratio
CACNG7
SCN3A
NPPC
KCNK2
CACNG4
SCN3B
SLN
SCN2A
RYR3
KCND3
SCN4B
RYR2
SCN5A
NPPA
KCNK6
KCNQ1
TCF7
TCF3
TCF7L1
TCF4
CTNNB1
TBX5
PITX2
NKX2-5
GATA6
Exp Log Ratio
f
DNA replication
Nicotine addiction
Mismatch repair
Homologous recombination
Fanconi anemia pathway
Morphine addiction
Glutamatergic synapse
Nucleotide excision repair
Synaptic vesicle cycle
Cell cycle
Amphetamine addiction
GABAergic synapse
Circadian entrainment
Signaling pathways regulating pluripotency of stem cells
Dopaminergic synapse
Neuroactive ligand-receptor interaction
Axon guidance
Retrograde endocannabinoid signaling
Wnt signaling pathway
Calcium signaling pathway
Fold Enrichment
-log10(FDR)
N. of Genes
g
Muscle structure development
Vasculature development
Actin filament-based process
Circulatory system development
Cell migration
Anatomical structure morphogenesis
Regulation of localization
Response to organic substance
Cellular response to chemical stimulus
Cellular localization
Fold Enrichment
-log10(FDR)
N. of Genes
h
25151 upregulate
19830 downregulated
Total=44981
i
exonic
promoter
5'-UTR
3'-UTR
Intergenic
23%
6%
15%
5%
51%
j
r = 0.54
p-val <0.0001
F=1346
P-val<0.0001
RNAseq Log FC
ATACseq Log FC
k
Axon guidance
Ferroptosis
Viral life cycle−HIV−1
Basal cell carcinoma
Signaling pathways regulating pluripotency of stem cells
Dilated cardiomyopathy
CGMP−PKG signaling pathway
Hypertrophic cardiomyopathy
Adrenergic signaling in cardiomyocytes
Glutamatergic synapse
Wnt signaling pathway
Circadian entrainment
CAMP signaling pathway
Cell adhesion molecules
Hepatocellular carcinoma
N. of Genes
−log10(FDR)
Fold Enrichment

Supplementary Figure 3: Transcriptomic and chromatin accessibility analysis between *LMNA p.S143P* iPSC-aCMs and non-isogenic population controls mirror the comparison with S143P-corrected isogenic iPSC-aCMs. a. Volcano plot showing differentially expressed genes (DEGs) ($FDR < 0.05$, $log_2FC > 0$) **b.** Altered cardiovascular signaling pathways in *LMNA p.S143P* iPSC-aCMs as identified through Ingenuity Pathway Analysis (IPA). **c-d.** Differential Gene expression changes in genes associated with cardiac conduction and cytoskeletal function. **f-g.** Gene Enrichment analysis in *LMNA* p.S143P iPSC-aCMs. **f.** KEGG Pathway enrichment analysis of upregulated genes. **g.** GO enrichment analysis of downregulated genes. Distribution of differential ATAC-seq peaks, illustrating that the majority are in non-coding genomic regions. **h.** Differential ATAC peaks. **i.** Distribution of differential ATAC peaks. **j.** Positive correlation between gene expression and chromatin accessibility at promoters of DEGs. Correlation analysis done using pearsons correlation and linear regression. Statistical significance at $p<0.05$ **k.** KEGG pathway analysis of differential ATAC peaks at promoter regions. n=3 biological replicates for both RNA and ATAC-seq.

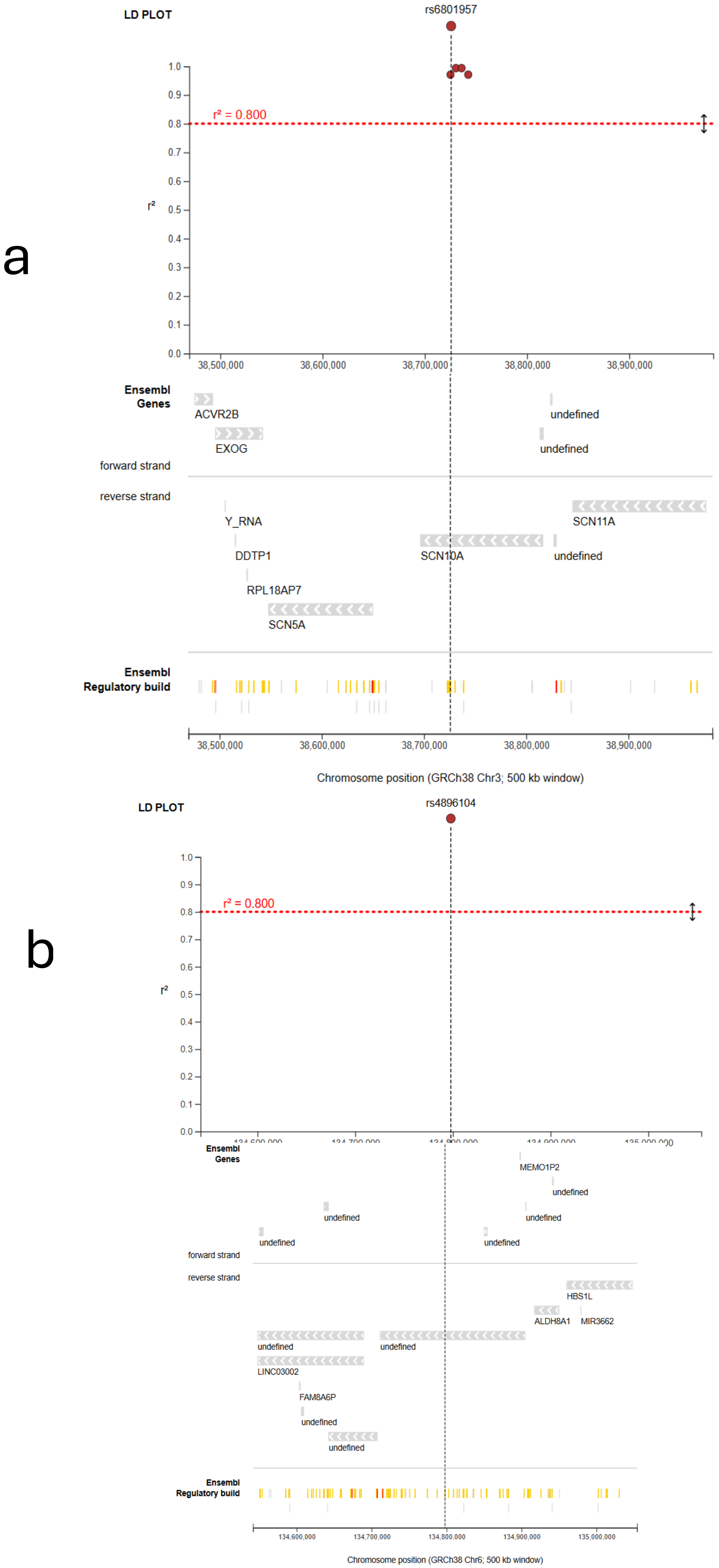
a
LD PLOT
rs6801957
r² = 0.800
r²
1.0
0.9
0.8
0.7
0.6
0.5
0.4
0.3
0.2
0.1
0.0
38,500,000
38,600,000
38,700,000
38,800,000
38,900,000
Ensembl Genes
ACVR2B
EXOG
undefined
undefined
forward strand
reverse strand
Y_RNA
DDTP1
RPL18AP7
SCN5A
SCN10A
SCN11A
undefined
Ensembl Regulatory build
38,500,000
38,600,000
38,700,000
38,800,000
38,900,000
Chromosome position (GRCh38 Chr3; 500 kb window)
b
LD PLOT
rs4896104
r² = 0.800
r²
1.0
0.9
0.8
0.7
0.6
0.5
0.4
0.3
0.2
0.1
0.0
Ensembl Genes
MEMO1P2
undefined
undefined
undefined
undefined
undefined
forward strand
reverse strand
HBS1L
ALDH8A1
MIR3662
undefined
undefined
LINC03002
FAM8A6P
undefined
undefined
Ensembl Regulatory build
134,600,000
134,700,000
134,800,000
134,900,000
135,000,000
Chromosome position (GRCh38 Chr6; 500 kb window)

c

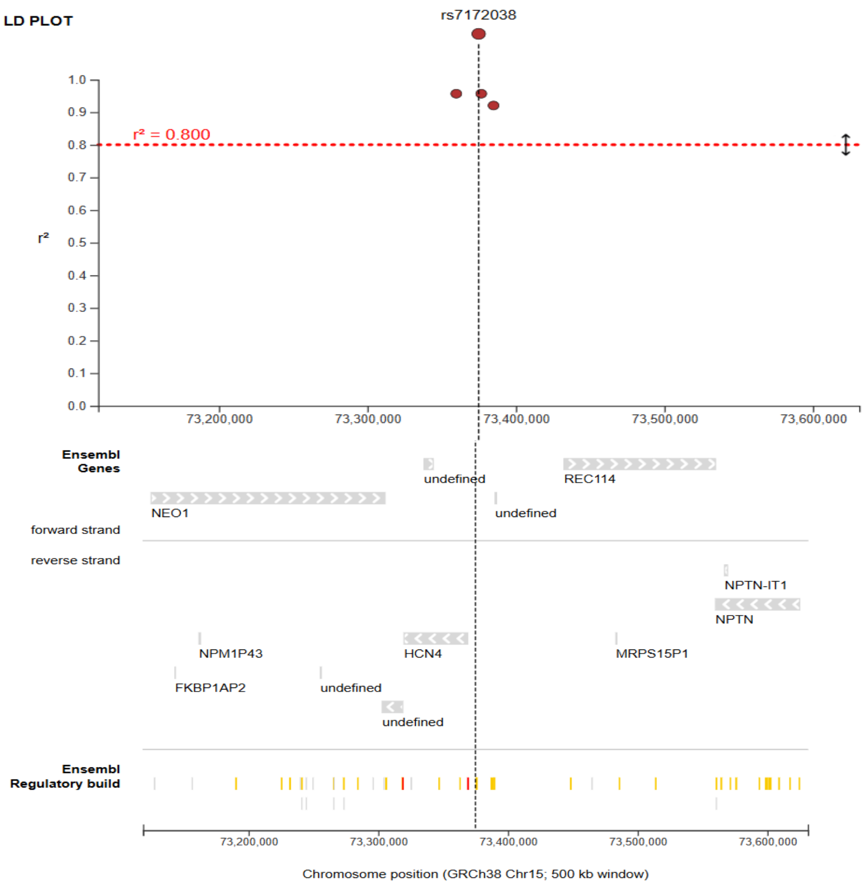

LD PLOT
rs7172038
r² = 0.800
r²
1.0
0.9
0.8
0.7
0.6
0.5
0.4
0.3
0.2
0.1
0.0
73,200,000
73,300,000
73,400,000
73,500,000
73,600,000
Ensembl Genes
undefined
REC114
NEO1
undefined
forward strand
reverse strand
NPTN-IT1
NPTN
NPM1P43
HCN4
MRPS15P1
FKBP1AP2
undefined
undefined
Ensembl Regulatory build
Chromosome position (GRCh38 Chr15; 500 kb window)

d

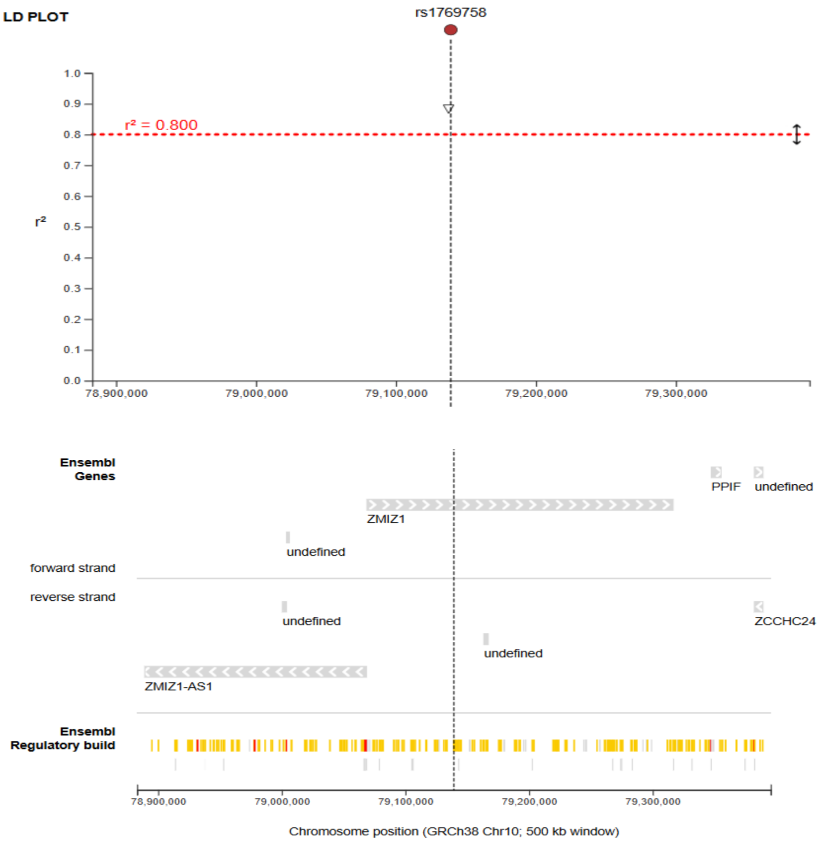

LD PLOT
rs1769758
r² = 0.800
r²
1.0
0.9
0.8
0.7
0.6
0.5
0.4
0.3
0.2
0.1
0.0
78,900,000
79,000,000
79,100,000
79,200,000
79,300,000
Ensembl Genes
PPIF
undefined
ZMIZ1
undefined
forward strand
reverse strand
undefined
ZCCHC24
undefined
ZMIZ1-AS1
Ensembl Regulatory build
Chromosome position (GRCh38 Chr10; 500 kb window)

e

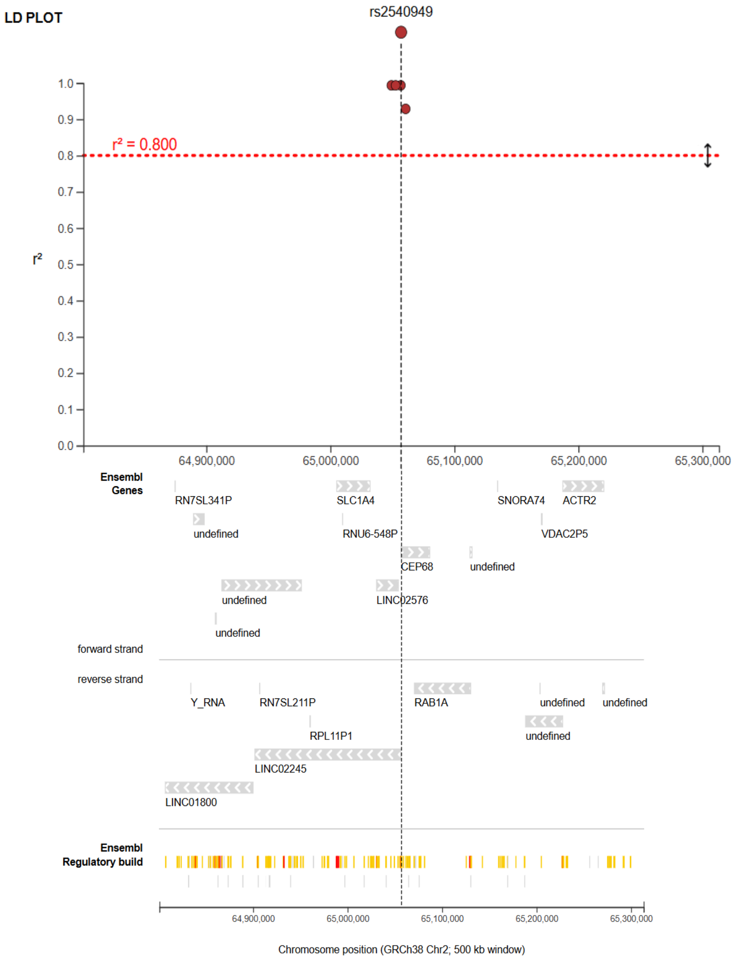
LD PLOT
rs2540949
r² = 0.800
r²
1.0
0.9
0.8
0.7
0.6
0.5
0.4
0.3
0.2
0.1
0.0
64,900,000
65,000,000
65,100,000
65,200,000
65,300,000
Ensembl Genes
RN7SL341P
SLC1A4
SNORA74
ACTR2
undefined
RNU6-548P
VDAC2P5
CEP68
undefined
undefined
LINC02576
undefined
forward strand
reverse strand
Y_RNA
RN7SL211P
RAB1A
undefined
undefined
RPL11P1
undefined
LINC02245
LINC01800
Ensembl Regulatory build
Chromosome position (GRCh38 Chr2; 500 kb window)

f

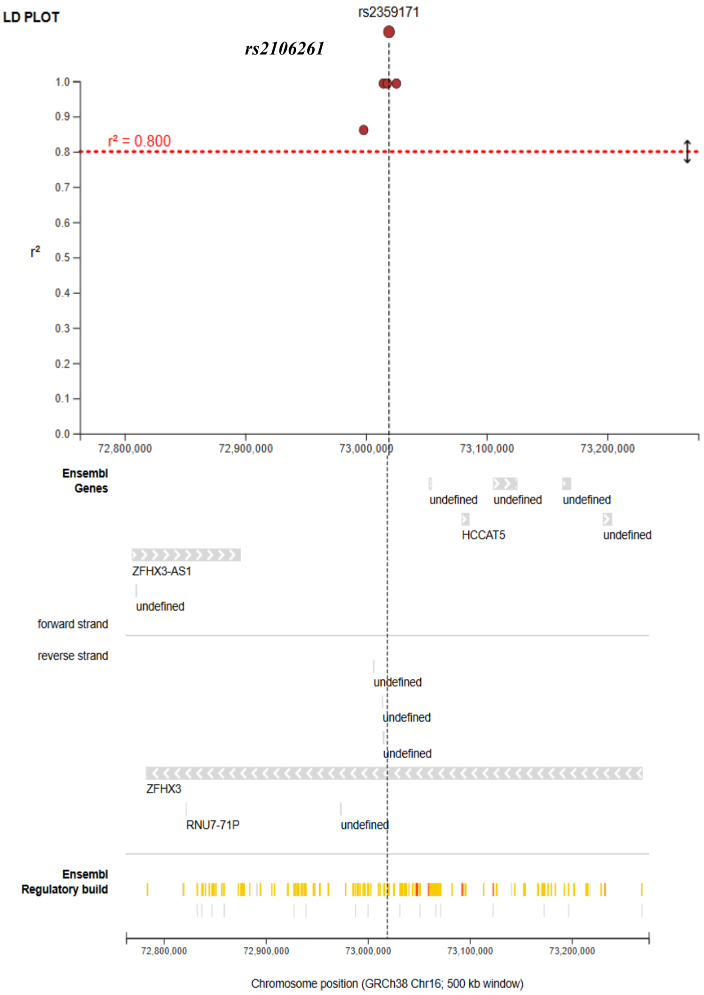
LD PLOT
rs2359171
rs2106261
r² = 0.800
r²
1.0
0.9
0.8
0.7
0.6
0.5
0.4
0.3
0.2
0.1
0.0
72,800,000
72,900,000
73,000,000
73,100,000
73,200,000
Ensembl Genes
undefined
undefined
undefined
HCCAT5
undefined
ZFHX3-AS1
undefined
forward strand
reverse strand
undefined
undefined
undefined
ZFHX3
RNU7-71P
undefined
Ensembl Regulatory build
Chromosome position (GRCh38 Chr16; 500 kb window)

g

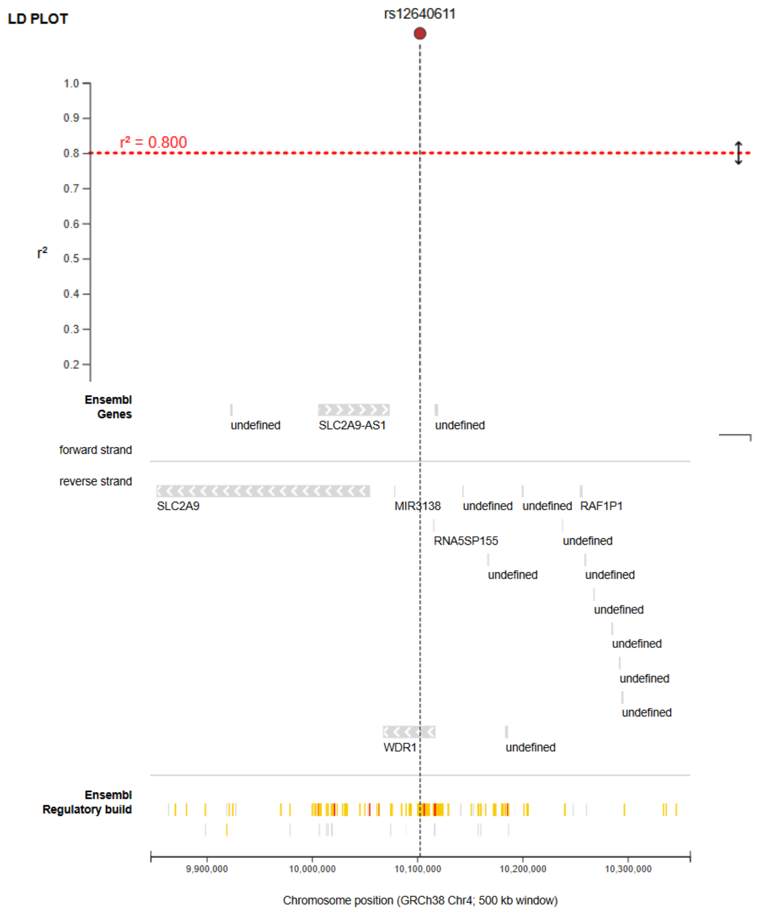
LD PLOT
rs12640611
r² = 0.800
r²
Ensembl Genes
forward strand
reverse strand
undefined
SLC2A9-AS1
SLC2A9
MIR3138
RAF1P1
RNA5SP155
WDR1
Ensembl Regulatory build
9,900,000
10,000,000
10,100,000
10,200,000
10,300,000
Chromosome position (GRCh38 Chr4; 500 kb window)

h

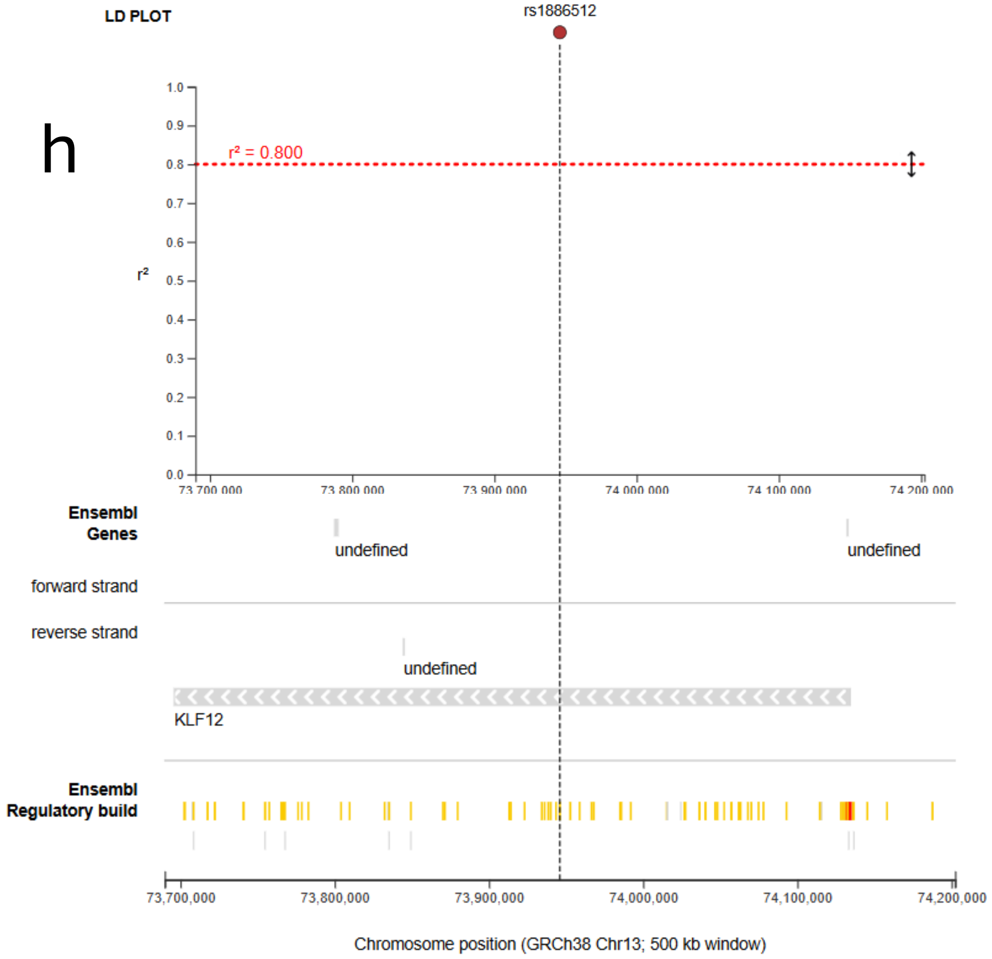
LD PLOT
rs1886512
r² = 0.800
r²
73 700 000
73 800 000
73 900 000
74 000 000
74 100 000
74 200 000
Ensembl Genes
forward strand
reverse strand
undefined
KLF12
Ensembl Regulatory build
73,700,000
73,800,000
73,900,000
74,000,000
74,100,000
74,200,000
Chromosome position (GRCh38 Chr13; 500 kb window)

Supplementary Figure 4: LD Plots for AF SNPs in regions of differential accessibility from the GWAS catalog.

GWAS: Catalog of Published Genome-Wide Association Studies (RRID:SCR_012745) under the license https://creativecommons.org/licenses/by/4.0/

Filtering criteria for SNPs in LD

- r^2=0.8
- associated with a cardiovascular trait
- Population: British in England and Scotland
- Within 50Kb

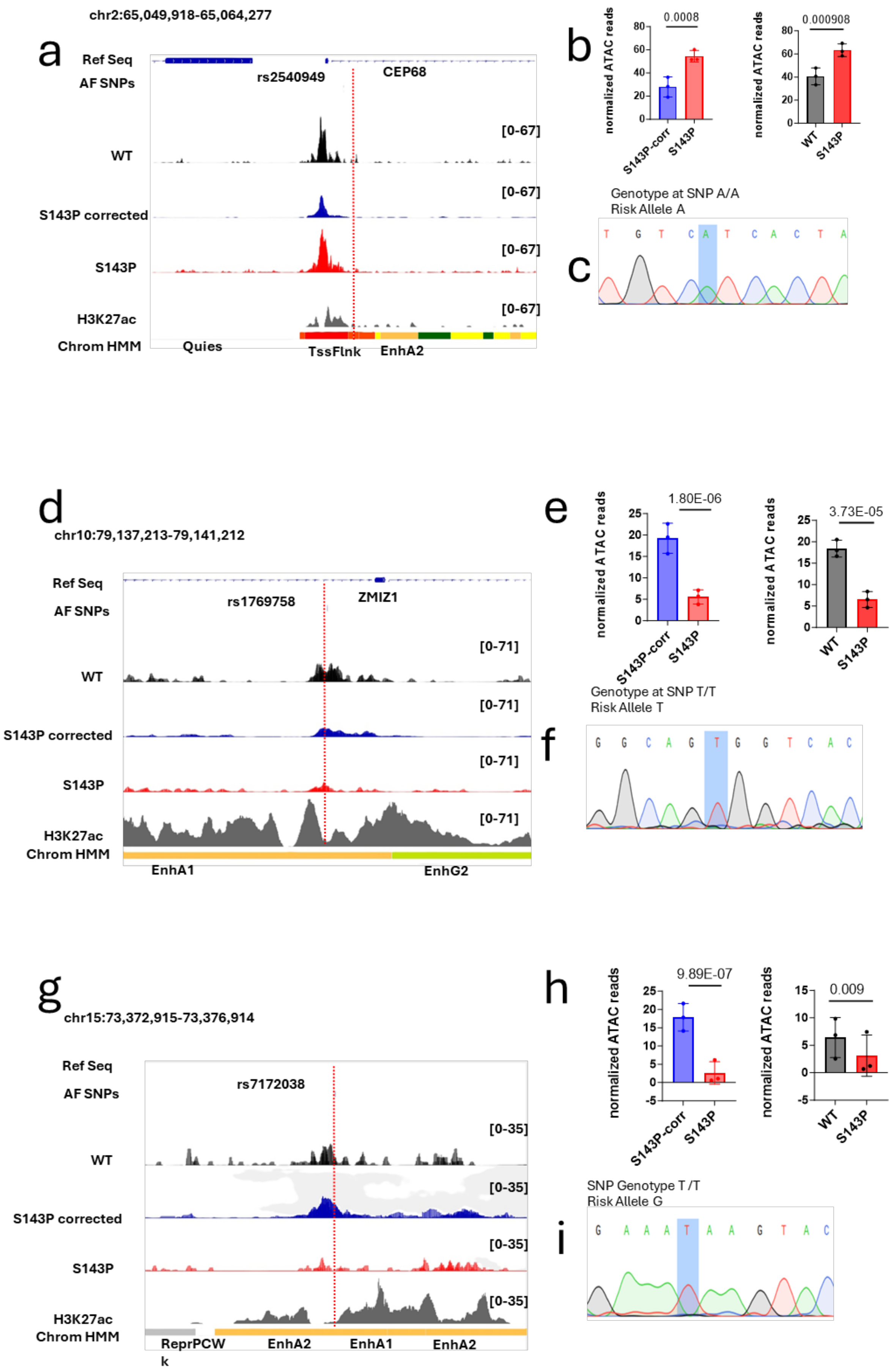

a
chr2:65,049,918-65,064,277
Ref Seq
AF SNPs
rs2540949
CEP68
WT
S143P corrected
S143P
H3K27ac
Chrom HMM
[0-67]
Quies
TssFlnk
EnhA2
b
normalized ATAC reads
0.0008
S143P-corr
S143P
0.000908
WT
S143P
c
Genotype at SNP A/A
Risk Allele A
d
chr10:79,137,213-79,141,212
rs1769758
ZMIZ1
[0-71]
EnhA1
EnhG2
e
1.80E-06
3.73E-05
f
Genotype at SNP T/T
Risk Allele T
g
chr15:73,372,915-73,376,914
rs7172038
[0-35]
ReprPCW
k
EnhA2
EnhA1
h
9.89E-07
0.009
i
SNP Genotype T /T
Risk Allele G

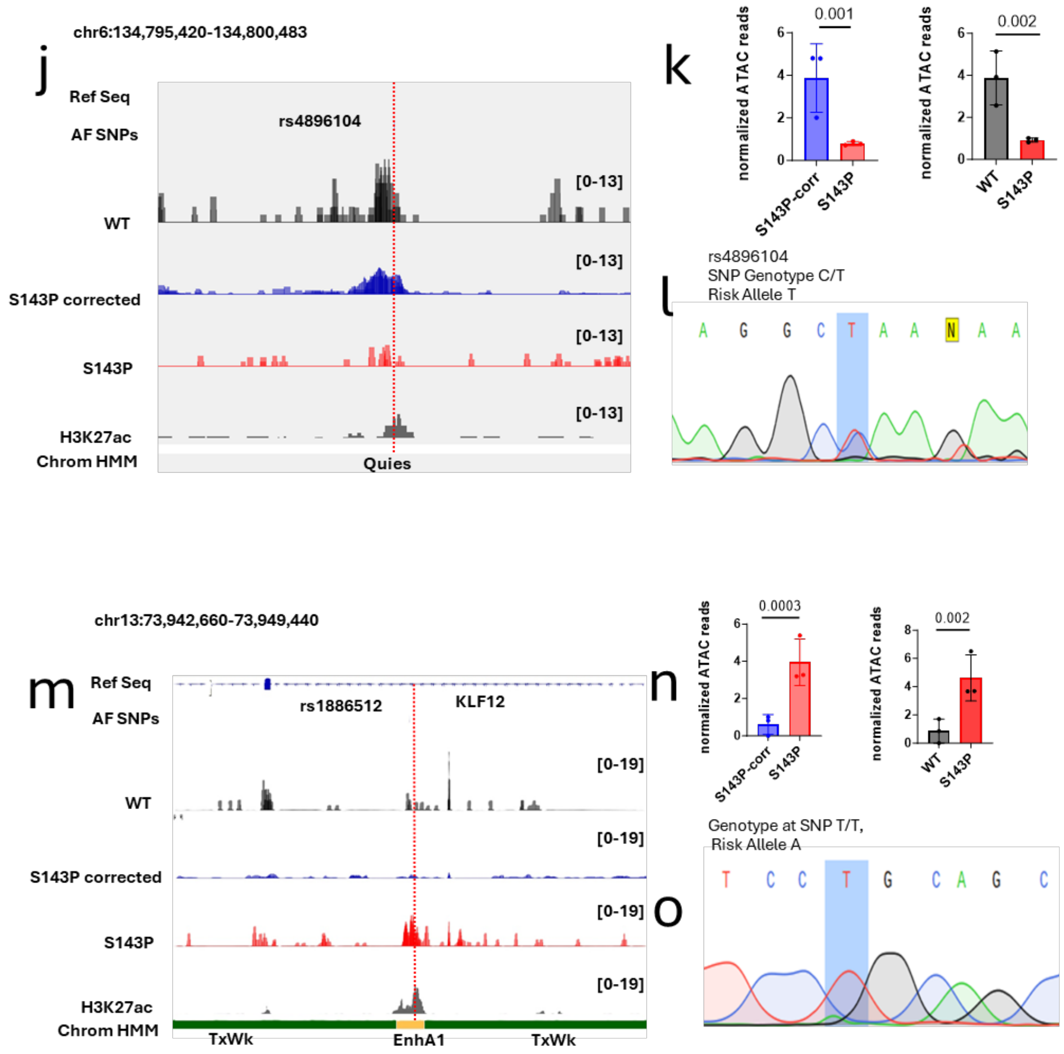

ChromHMM State Descriptions

p

State description	Abbrev.
Active TSS	TssA
Flanking TSS	TssFlnk
Flanking TSS upstream	TssFlnkU
Flanking TSS downstream	TssFlnkD
Strong transcription	Tx
Weak transcription	TxWk
Genic enhancer 1	EnhG1
Genic enhancer 2	EnhG2
Active enhancer 1	EnhA1
Active enhancer 2	EnhA2
Weak enhancer	EnhWk
ZNF genes & repeats	ZNF/Rpts
Heterochromatin	Het
Bivalent/poised TSS	TssBiv
Bivalent enhancer	EnhBiv
Repressed Polycomb	ReprPC
Weak repressed Polycomb	ReprPCWk
Quiescent/low	Quies

Supplementary Figure. 5: Altered chromatin accessibility at regulatory regions harboring AF associated SNPs in *LMNA*-S143P iPSC-aCMs. Integrated genome viewer ATAC-seq track plots, Normalized ATAC reads and genotype of proband at differentially accessible regions with AF associated SNPs located in regulatory regions marked by H3K27ac and ChromHMM annotations.. **a-c.** rs2540949 **d-f**rs1769758**g-i**.rs7172038 **j-l**. rs4896104 **m-o**. rs1769758 **p-r**. rs1886512. H3K27 ChIP-seq track from human atrial tissue (ENCSR074ECR) . The dashed line shows the location of the SNP. Genomic location according to GR Ch38/hg38). **b,e,h,k,n**: Quantification of normalized ATAC-seq reads, n=3 biological replicates, FDR corrected P-values displayed , threshold at 0.05.

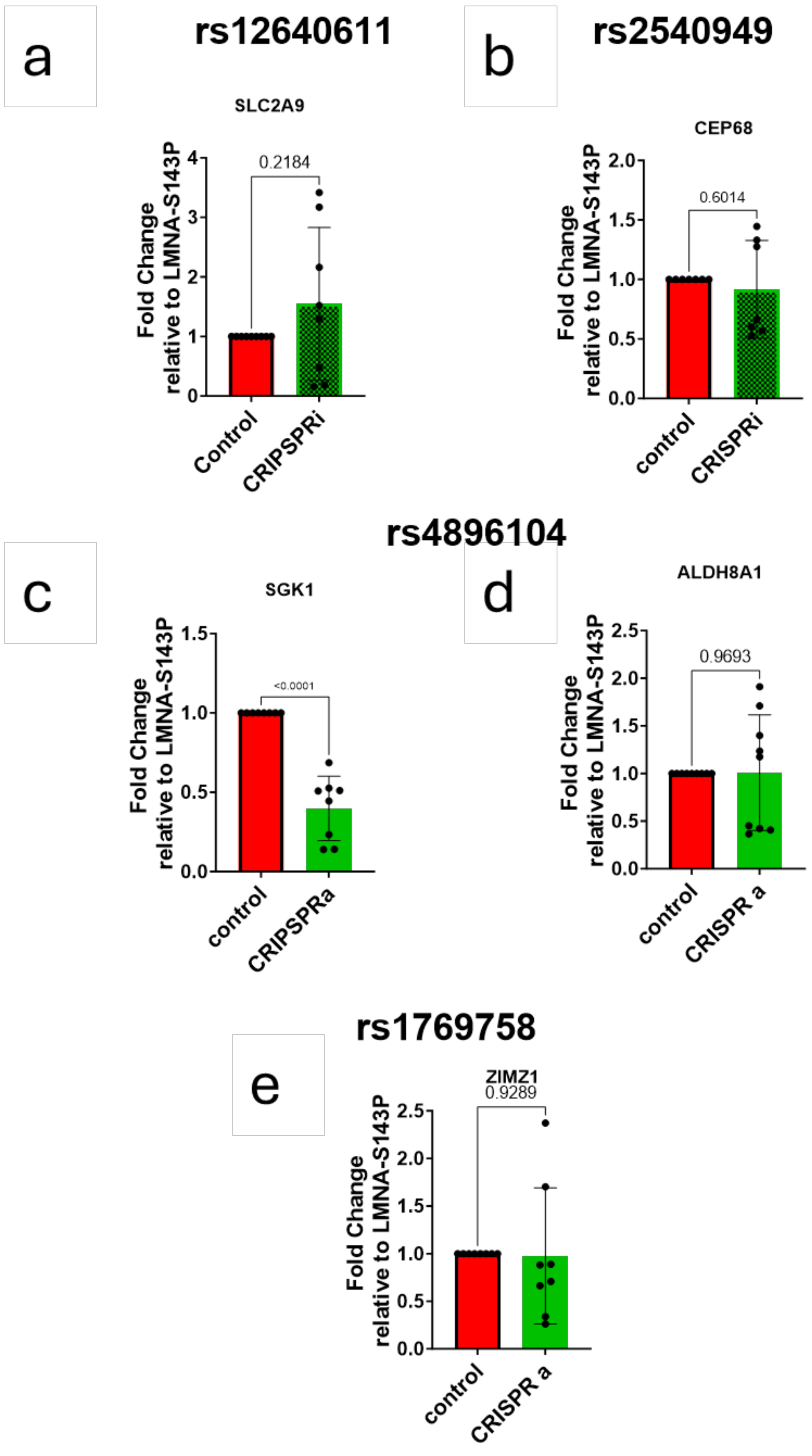
a
rs12640611
SLC2A9
0.2184
Fold Change relative to LMNA-S143P
Control
CRIPSPRi
b
rs2540949
CEP68
0.6014
Fold Change relative to LMNA-S143P
control
CRISPRi
rs4896104
c
SGK1
<0.0001
Fold Change relative to LMNA-S143P
control
CRIPSPRa
d
ALDH8A1
0.9693
Fold Change relative to LMNA-S143P
control
CRISPR a
rs1769758
e
ZIMZ1
0.9289
Fold Change relative to LMNA-S143P
control
CRISPR a

Supplementary Figure 6. Variant regions that did not show a change in expression on CRISPR epigenetic modulation. Transcript expression determined by qPCR after CRISPRa/i of the variant region compared to transfection controls. “n values”: *SLC2A9*=9, *CEP68*=7. *SGK1*=8, *ALDH8A1*=9, *ZIMZ1*=8 generated from at three biological replicates. Data represented as +/- SD. Statistical comparison done using unpaired t-test.Significance at P<005.

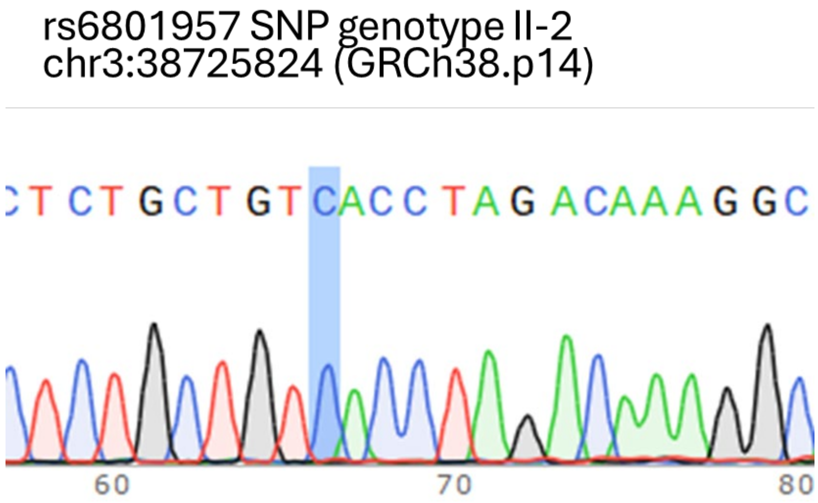

Supplementary Figure 7: Genotyping of AF-associated SNP rs6801957 in family member II-2.

a

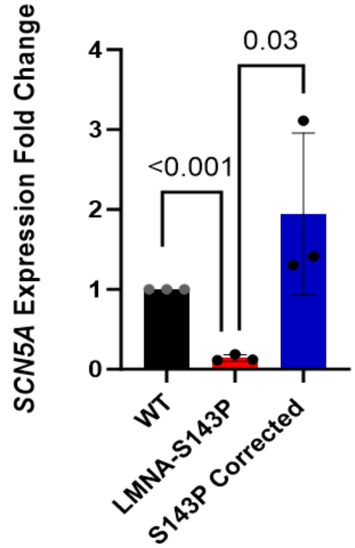
SCN5A Expression Fold Change
0.03
<0.001
4
3
2
1
0
WT
LMNA-S143P
S143P Corrected

b

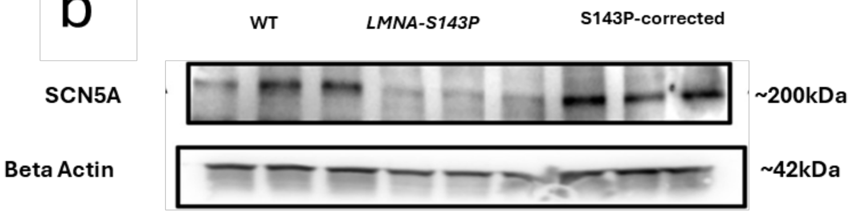
WT
LMNA-S143P
S143P-corrected
SCN5A
~200kDa
Beta Actin
~42kDa

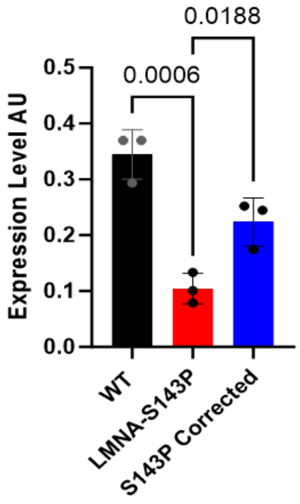
0.0188
0.0006
Expression Level AU
0.5
0.4
0.3
0.2
0.1
0.0
WT
LMNA-S143P
S143P Corrected

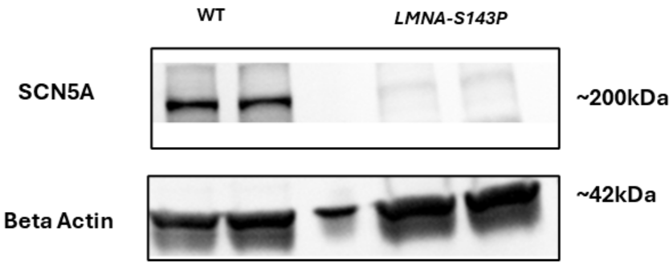
WT
LMNA-S143P
SCN5A
~200kDa
Beta Actin
~42kDa

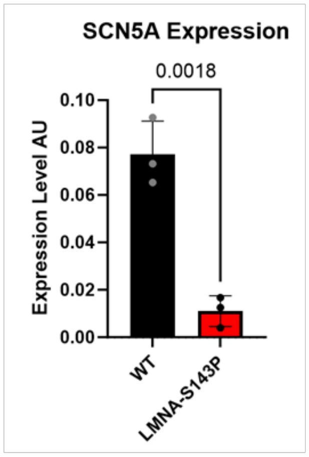
SCN5A Expression
0.0018
Expression Level AU
0.10
0.08
0.06
0.04
0.02
0.00
WT
LMNA-S143P

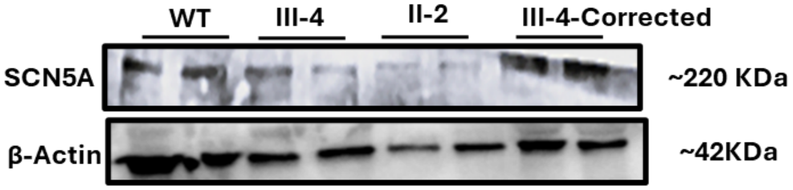
WT
III-4
II-2
III-4-Corrected
SCN5A
~220 KDa
β-Actin
~42KDa

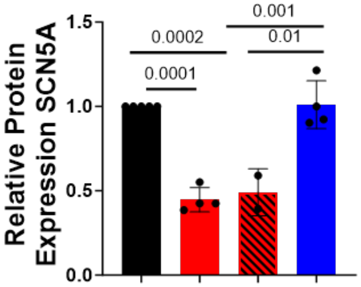
Relative Protein Expression SCN5A
1.5
1.0
0.5
0.0
0.001
0.0002
0.01
0.0001

Supplementary Figure 8: a. Reduced mRNA expression of *SCN5A* in *LMNA* p.S143P iPSC-aCMs by qPCR. **b.** Reduced protein expression of *SCN5A* in *LMNA* p.S143P iPSC-aCMs in samples from multiple differentiations. a-b : N=3 biological replicates across all groups. Statisitical comparisons for two groups done by unpaired t-test, for multiple groups, one way ANOVA with multiple comparisons. Significance at $P<0.05$. Data represent +/- SD.

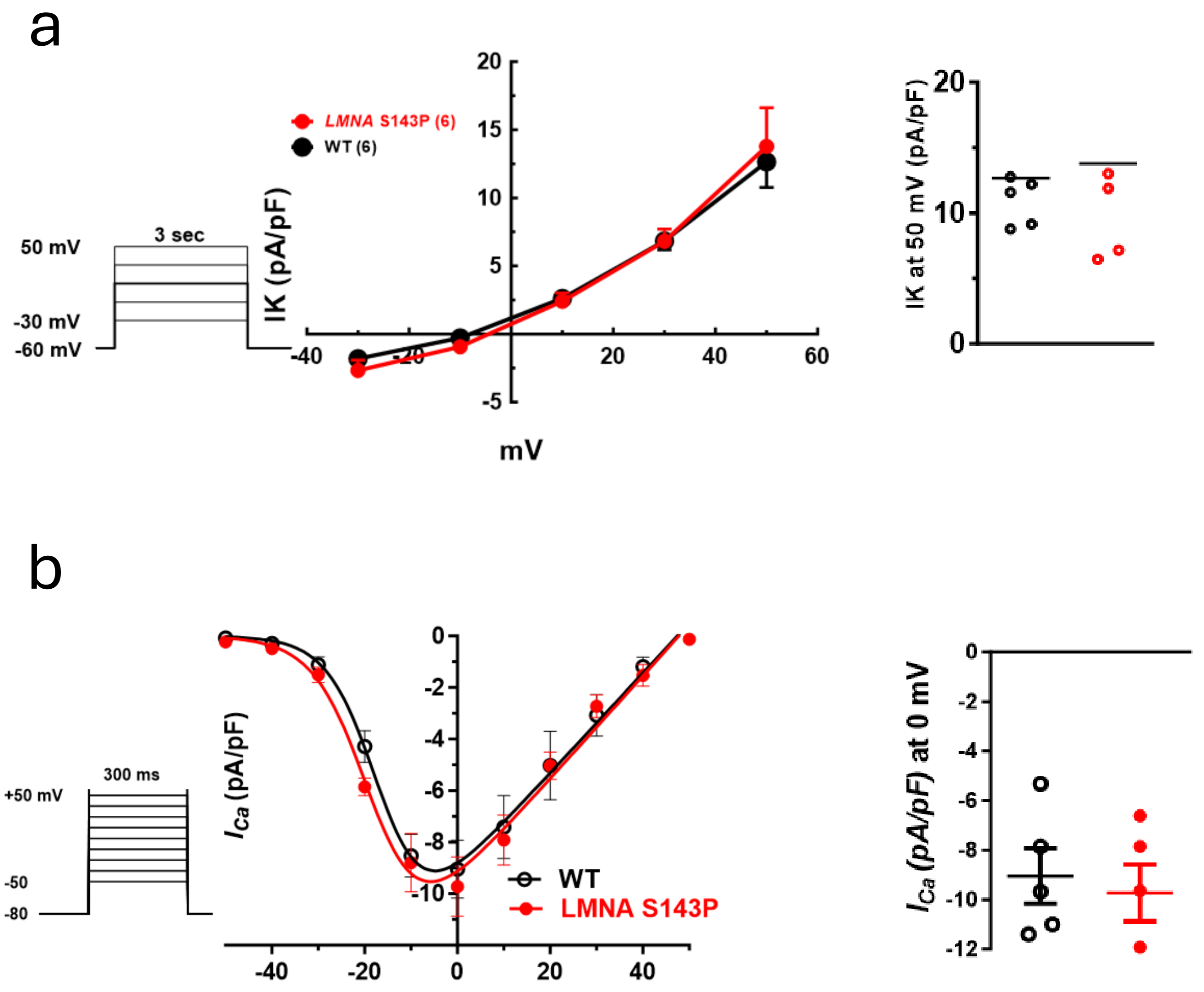

Supplementary Figure 9: ***LMNA* p.S143P does not alter potassium (*I_K*) or calcium (*$I_{Ca,L}$*) currents. a.** IV relationship for *I_K* in WT, *LMNA* p.S143P iPSC-aCMs. **b.** IV relationship for I_{CaL} in the two groups.   a-b : N=4 biological replicates across all groups. Statisitical comparisons for two groups done by unpaired t-test, . Significance at P<0.05. Data represent +/- SD

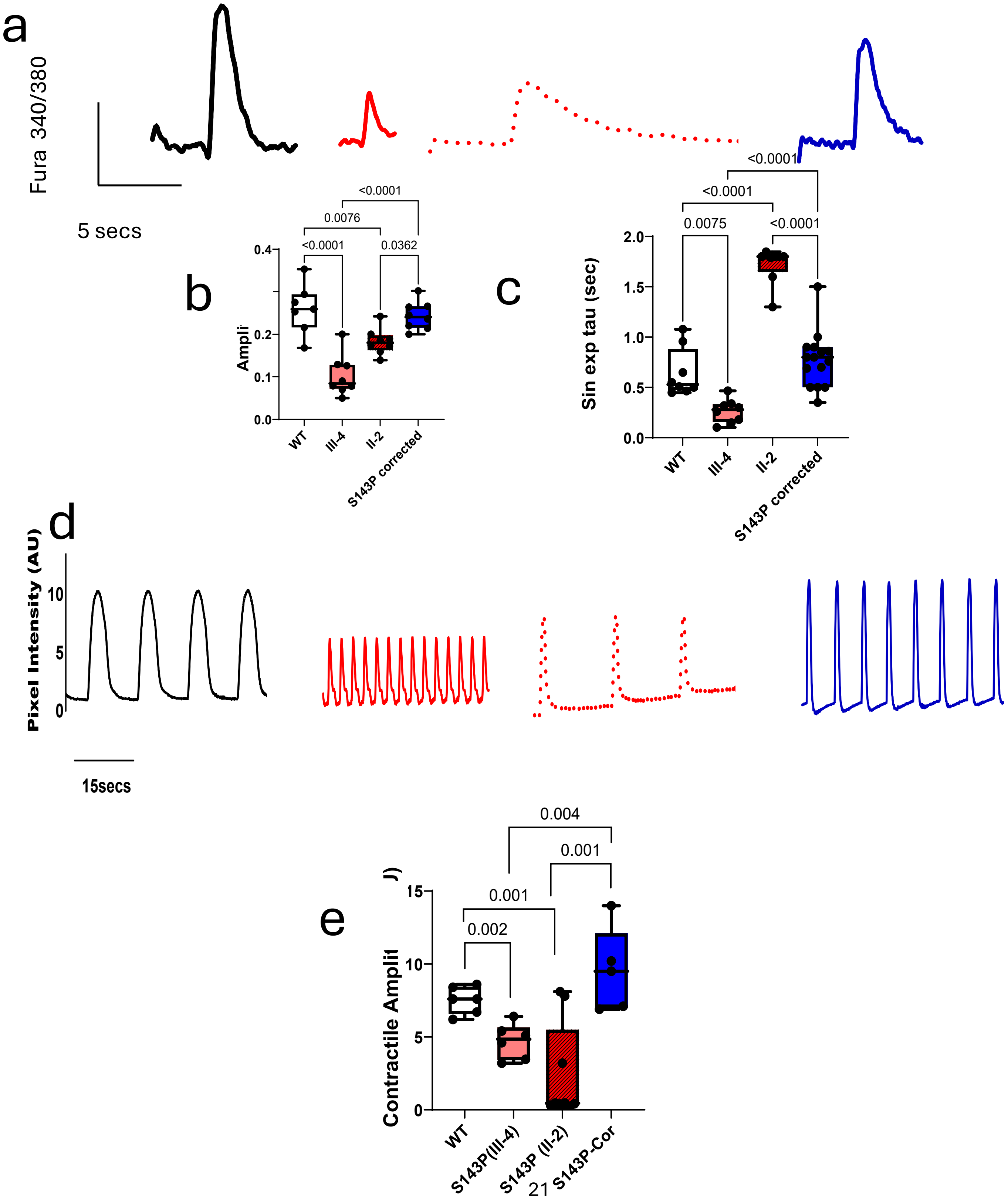

a
Fura 340/380
5 secs
b
Ampli
0.4
0.2
0.1
0.0
<0.0001
0.0076
<0.0001
0.0362
WT
III-4
II-2
S143P corrected
c
Sin exp tau (sec)
2.0
1.5
1.0
0.5
0.0
<0.0001
<0.0001
0.0075
<0.0001
WT
III-4
II-2
S143P corrected
d
Pixel Intensity (AU)
10
5
0
15secs
e
Contractile Ampli
15
10
5
0
0.004
0.001
0.001
0.002
WT
S143P(III-4)
S143P (II-2)
S143P-Cor

Supplementary Figure 10: Calcium transient aberrations and decreased contractility in *LMNA* p.S143P iPSC-aCMs. a. Representative traces of calcium transients. **b-c.** Transient amplitude is reduced in *LMNA* p.S143P iPSC-aCMs; in III-4, the decay time is shortened, whereas it is prolonged in II-2, compared to isogenic and WT controls. **d.** *LMNA* p.S143P iPSC-aCMs have reduced contractile amplitude compared to WT and isogenic control.(**b:** WT, n=8. II-2, n=8, III-4,n=11, III-4 corrected n=8 . **c:** WT, n=8. II-2, n=8, III-4,n=8, III-4 corrected n=15 . **e:** WT, n=6. II-2, n=6, III-4,n=5, III-4 corrected n=10 recordings from at least three biological replicates) **b, c, e:** Statistical comparisons done one-way ANOVA with multiple comparisons. Significance at P<0.05. The box plot extends from the 25th to the 75th percentile. The line in the middle represents the median. The whiskers go down to the min and max points.

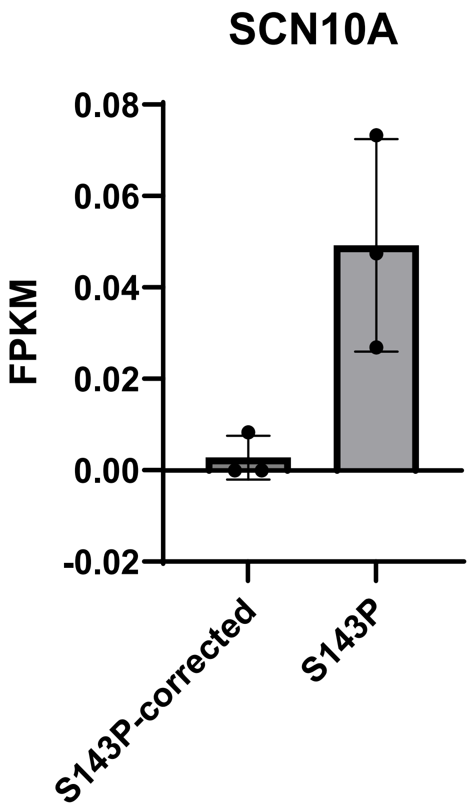

Fig. F 11: Minimal expression of *SCN10A* full-length detected from RNA sequencing.

N=3 biological replicates. FPKM-fold per kilo million.

Supplementary Table 1: Risk of AF in UK Biobank participants who do not harbor a PAV in *LMNA* by PRS category (n=419,087).

	No AF	Has AF	HR (p-value)
Low PRS (bottom 20%)	81246	1881	1.00
Mid PRS (20-80%)	240175	11178	1.94 ($p < 2 \times 10^{-16}$)
High PRS (top 20%)	76749	7860	4.11 ($p < 2 \times 10^{-16}$)

Supplementary Table 2: Risk of AF in carriers of PAVs in *LMNA* in the UK Biobank by PRS category (n=1,109).

	No AF	Has AF	HR (p-value)
Low PRS (bottom 20%)	216	5	1.00
Mid PRS (20-80%)	617	52	3.54 ($p = 7.49 \times 10^{-3}$)
High PRS (Top 20%)	187	32	7.18 ($p = 5.15 \times 10^{-5}$)

Supplementary Table 3: Risk of AF in young (<65 years) in *LMNA* PAV carriers compared to non-*LMNA* carriers.

	No AF	Has AF	HR (p-value)
Non-LMNA carriers	320470	11847	1.56 ($p = 3.01 \times 10^{-3}$)
LMNA carriers	823	45	

Supplementary Table 4: Risk of AF in the young (< 65 yo) UK Biobank non-*LMNA* carriers by PRS category.

	No AF	Has AF	HR (p-value)
Low PRS (bottom 20%)	64842	1005	1.00
Mid PRS (20-80%)	193150	6193	1.96 ($p < 2 \times 10^{-16}$)
High PRS (top 20%)	62478	4649	4.4 ($p < 2 \times 10^{-16}$)

Supplementary Table 5: Risk of AF in young (<65 years) in UK Biobank PAVs in LMNA carriers by PRS category.

	No AF	Has AF	HR (p-value)
Low PRS (bottom 20%)	170	3	1.00
Mid PRS (20-80%)	511	23	3.08 ($p = 7.11 \times 10^{-2}$)
High PRS (top 20%)	142	19	9.31 ($p = 4.73 \times 10^{-4}$)

Supplementary Table 6: Risk of AF in UK Biobank P/LP LMNA carriers by PRS category.

	No AF	Has AF
Low PRS (bottom 20%)	5	0
Mid PRS (20-80%)	17	2
High PRS (top 20%)	9	4

Supplementary Table 7: Overlap of AF risk loci and ATAC peaks in *LMNA* p.S143P iPSC-aCMs vs WT-iPSC-aCMs.

rsID	Nearest_Gene(s)†	Chromosome	ATAC FC	p-value
rs4896104	ALDH8A1,SGK1	chr6	-2.07327	0.004304
rs55734480	DGKB	chr7	-1.9301	0.008852
rs7172038	NEO1,HCN4, NPTN	chr15	-1.73881	0.009063
rs6801957	SCN10A	chr3	-1.40184	0.00035
rs1769758	ZMIZ1	chr10	-1.3854906	3.73E-05
rs2540949	CEP68	chr2	0.634214	0.000908
rs12640611	WDR1, SLC2A9	chr4	2.067337	8.50E-09
rs1886512	KLF12	chr13	2.179281	0.002199

Supplementary Table 8: Overlap of AF risk loci and ATAC peaks in *LMNA* p.S143P iPSC-aCMs vs S143P-Corrected iPSC-aCMs.

rs ID	Nearest Gene	Chromosome	ATAC FC	p-value
rs4896104	ALDH8A1, SGK1	Chr6	-2.30526457	0.001
rs7172038	HCN4, NPTN, NEO1	Chr15	-3.10931262	9.89E-07
rs6801957	SCN10A	chr3	-1.34845362	0.003
rs1769758	ZMIZ1	chr10	-1.68391058	1.80E-06
rs2540949	CEP68	chr2	0.9319087	0.0008
rs12640611	SLC2A9, WDR1	ch4	1.88833119	2.03E-05
rs1886512	KLF12	chr13	2.54274979	0.0003
rs2359171	ZFHX3	ch16	-1.60223123	0.0003

Supplementary Table 9: Proband's genotype at AF SNPs in differentially accessible regions.

SNP	Mapped Gene	Proband's Genotype	Risk Allele
rs6801957	SCN5A/SCN10A	C/C	C
rs2540949	CEP68	A/A	A
rs2359171	ZFHX3	T/A	A
rs1769758	ZMIZ1	T/T	T
rs12640611	WDR1, SLC2A9	C/C	C
rs4896104	ALDH8A1, SGK1	C/T	T
rs1886512	KLF12	T/T	A
rs7172038	REC114, HCN4	T	G

Risk allele annotation based on cross-ancestry analysis by Miyazawa et al.[1]

Supplementary Table 10: Risk alleles of AF variants overlapping with Differentially Accessible Regions from Miyazawa et al[1].

https://creativecommons.org/licenses/by/4.0/

CHR	POS	Locus		REF	ALT	rsID	Nearby gene	Annotated gene*	Functional consequence	$log_{10}BF$	BBJ				EUR				FIN			
		Start	End								AAF	Beta	SE	P	AAF	Beta	SE	P	AAF	Beta	SE	P
1	4E+07	4E+07	4E+07	T	C	rs6801957	*SCN5A*	*SCN10A*	intronic	22.109	0.784	0.066	0.018	3.21E-04	0.602	0.062	0.007	6.87E-20	0.546	0.052	0.019	6.32E-03
2	7E+07	6E+07	7E+07	A	T	rs2540949	*CEP68*	*CEP68*	intronic	31.262	0.325	-0.089	0.016	3.07E-08	0.385	-0.066	0.007	2.95E-22	0.340	-0.100	0.020	4.80E-07
3	8E+07	8E+07	8E+07	G	T	rs1769758	*ZMIZ1*	*ZMIZ1*	intronic	7.505	0.715	0.052	0.017	2.72E-03	0.490	0.034	0.008	5.38E-06	0.501	0.062	0.019	1.11E-03
4	1E+07	1E+07	1E+07	T	C	rs12640611	*WDR1*	*WDR1*	intronic	7.628	0.579	0.048	0.015	1.68E-03	0.684	0.037	0.007	4.56E-07	0.682	0.030	0.020	1.34E-01
5	1E+08	1E+08	1E+08	C	T	rs4896104	*LOC101928304, ALDH8A1*	*ALDH8A1*	intergenic	7.975	0.829	-0.052	0.020	8.17E-03	0.556	-0.037	0.007	7.51E-08	0.645	-0.036	0.020	7.14E-02
6	7E+07	7E+07	7E+07	T	G	rs7172038	*HCN4, REC114*	*NEO1*	intergenic	36.884	0.030	0.020	0.044	6.42E-01	0.160	0.112	0.009	4.78E-36	0.207	0.097	0.023	2.57E-05
7	7E+07	7E+07	8E+07	T	A	rs1886512	*KLF12*	*KLF12*	intronic	6.943	0.195	0.047	0.018	1.08E-02	0.357	0.036	0.007	2.81E-07	0.360	0.024	0.020	2.29E-01

Supplementary Table 11: Approach for prioritization of enhancers and target genes.

Index SNP	Evidence of a functional SNP in LD	Mapped Gene based on proximity	eQTL*		ATAC Fold Change	P-Value	Differential Expression in LMNA-S143P iPSC-aCMS	H3K27ac	PC-Hi C	Chromatin State	Supporting evidence of SNP -gene pair from previous studies	Ref
			HAA	LV								
rs4896104		ALDH8A1, SGK1			-2.30	0.001		+	+	Quiescent		
rs7172038		HCN4, NPTN			-3.109	9.89E-07	+	+	+	Enhancer	Gene PIP 0.7 SNP PIP 0.9	2
							+				STARR-seq and confirmation with deletion of the orthologous region in mouse	3
											Custom prioritization score (TAD, PCHiC, heart tissue expression adult and human, eQTL)	4
rs6801957		SCN10A		+	-1.34	0.003		+		Enhancer	SCN10A PIP 0.5<br>SCN5A PIP 0.4,<br>SNP PIP 0.8	2
											STARR-seq	3
rs1769758		ZMIZ1	+		-1.68	1.80E-06		+		Enhancer	SC-ATAC seq to identify OCR, Statistical Fine Mapping	2
rs2540949	rs1009358	CEP68	+	+	0.93	0.0008	+	+		TSSfl	Gene PIP 0.2, SNP PIP 0.2	2
rs12640611		SLC2A9	+		1.88	2.03E-05		+		Enhancer		
rs1886512		KLF12	+	+	2.54	0.0003		+		Enhancer		
rs2359171	rs2106261	ZFHX3			-1.60	0.0003	+	+		Enhancer	Gene PIP 0.8<br>rs2106261 PIP 1.0	2

1 Miyazawa, K. *et al.* Cross-ancestry genome-wide analysis of atrial fibrillation unveils disease biology and enables cardioembolic risk prediction. *Nature Genetics* **55**, 187-197 (2023). https://doi.org:10.1038/s41588-022-01284-9
2 Selewa, A. *et al.* Single-cell genomics improves the discovery of risk variants and genes of atrial fibrillation. *Nature Communications* **14**, 4999 (2023). https://doi.org:10.1038/s41467-023-40505-5
3 van Ouwerkerk, A. F. *et al.* Identification of Functional Variant Enhancers Associated With Atrial Fibrillation. *Circ Res* **127**, 229-243 (2020). https://doi.org:10.1161/circresaha.119.316006
4 van Ouwerkerk, A. F. *et al.* Identification of atrial fibrillation associated genes and functional non-coding variants. *Nat Commun* **10**, 4755 (2019). https://doi.org:10.1038/s41467-019-12721-5
