# Supplementary material for: Gene-gene interactions between a LMNA variant and common polymorphisms drive early-onset atrial fibrillation
Source: Nat Commun. 2026 May 19;17:6594. doi: 10.1038/s41467-026-73113-0 (PMC13381584; doi:10.1038/s41467-026-73113-0)
Supplement: Supplementary file 2 — Description of Additional Supplementary Files [file 41467_2026_73113_MOESM2_ESM.pdf]

Description of Additional Supplementary Files

Supplementary Data 1: sgRNAs sequences
