# Supplementary material for: Gene-gene interactions between a LMNA variant and common polymorphisms drive early-onset atrial fibrillation
Source: Nat Commun. 2026 May 19;17:6594. doi: 10.1038/s41467-026-73113-0 (PMC13381584; doi:10.1038/s41467-026-73113-0)
Supplement: Supplementary file 5 — Source Data [file 41467_2026_73113_MOESM5_ESM.zip › Source File Main Figures/Unedited Blots and TEM images.pptx]

## Slide 1
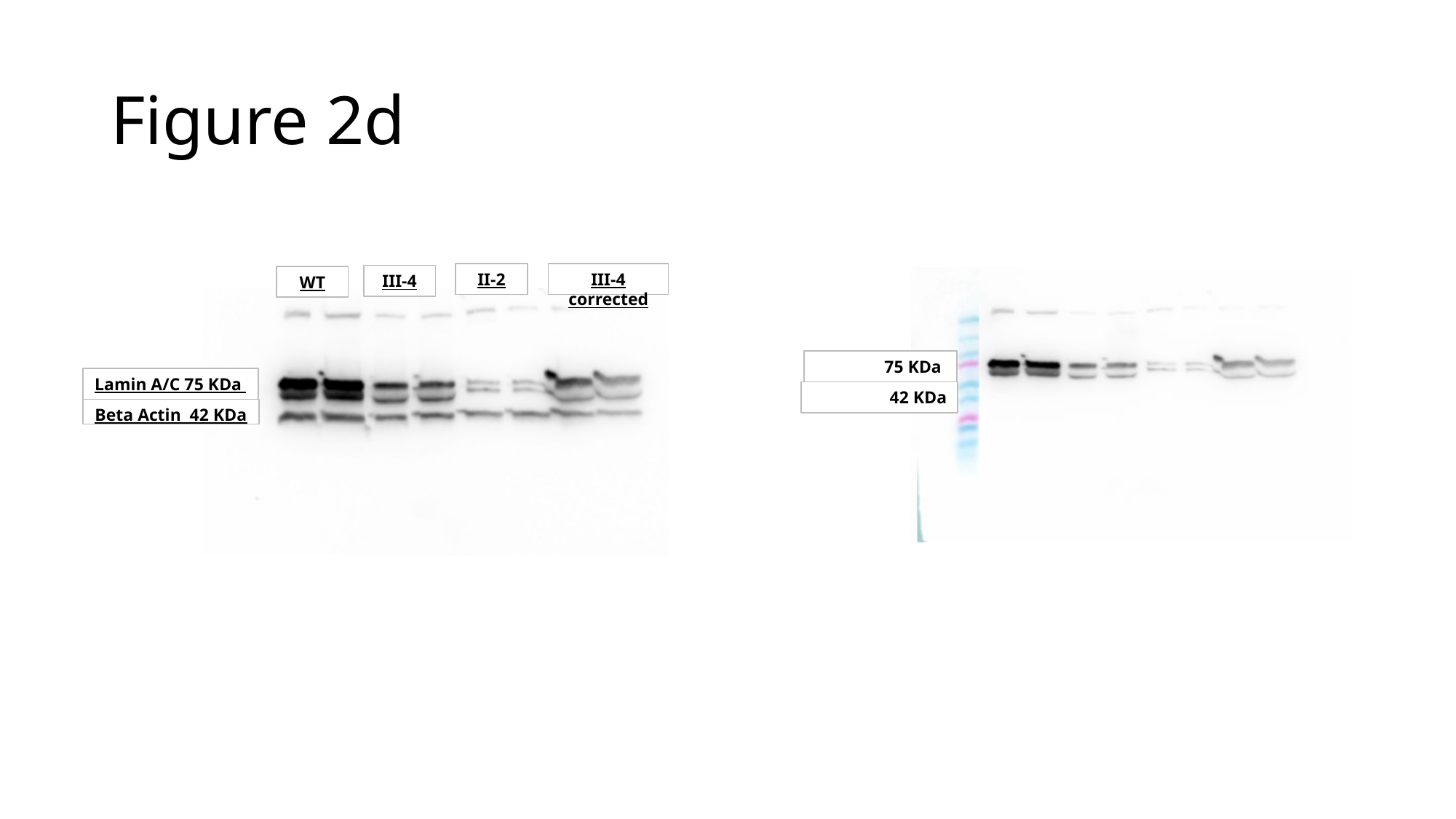

# Figure 2d
II-2
III-4 corrected
III-4
WT
75 KDa
Lamin A/C 75 KDa
42 KDa
Beta Actin 42 KDa

## Slide 2
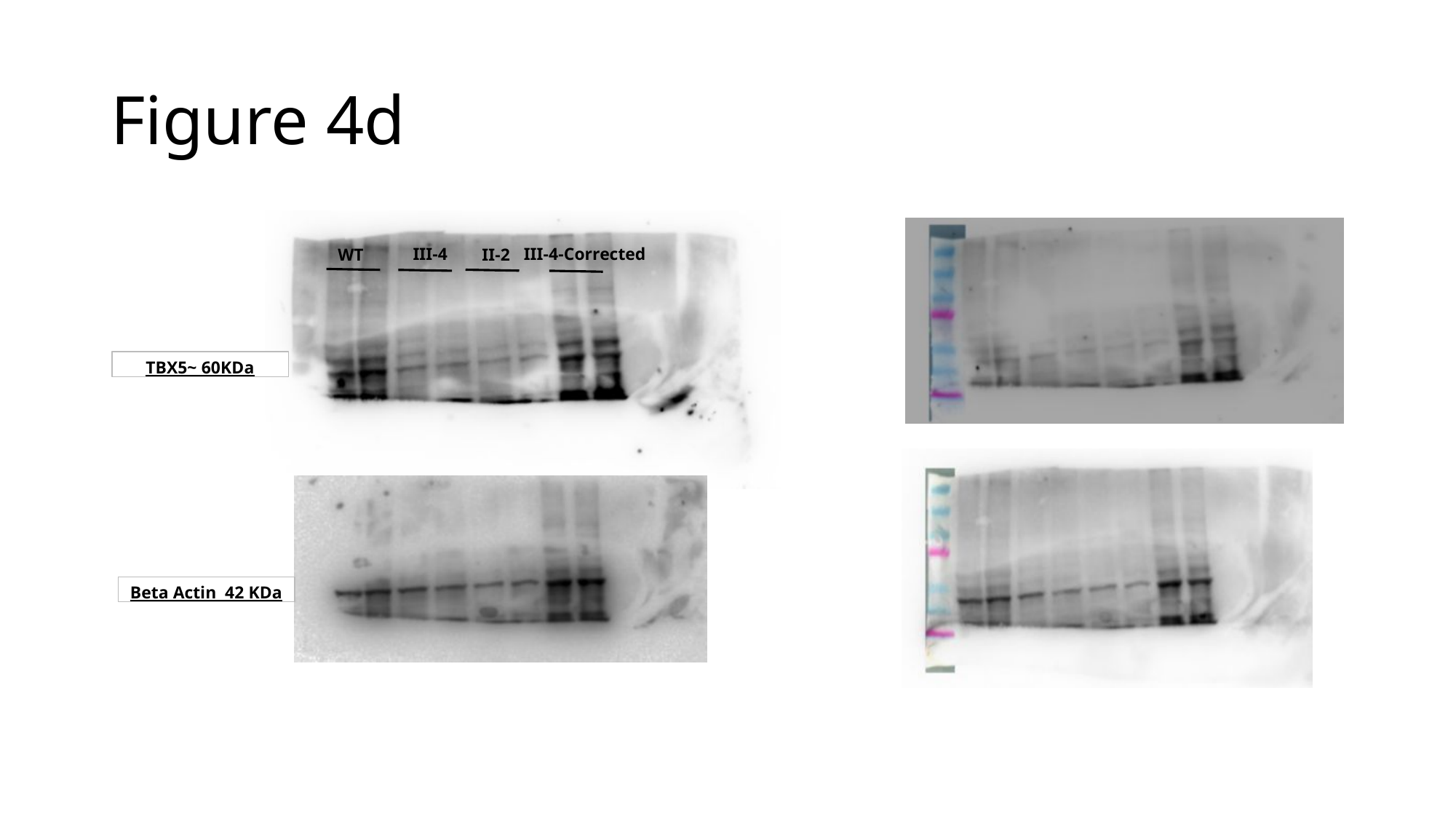

# Figure 4d
III-4
III-4-Corrected
WT
II-2
TBX5~ 60KDa
Beta Actin 42 KDa

## Slide 3
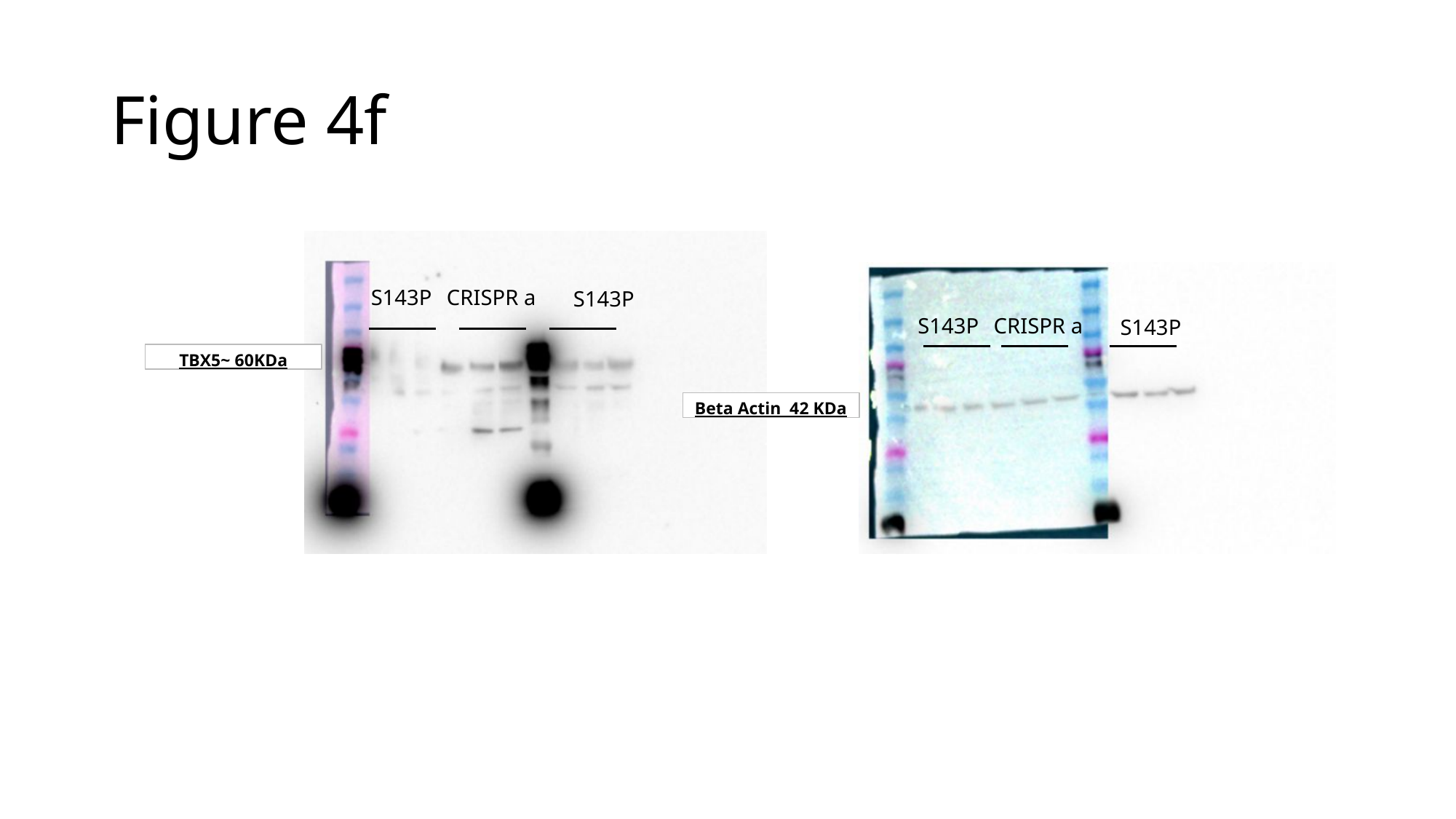

# Figure 4f
S143P
CRISPR a
S143P
S143P
CRISPR a
S143P
TBX5~ 60KDa
Beta Actin 42 KDa

## Slide 4
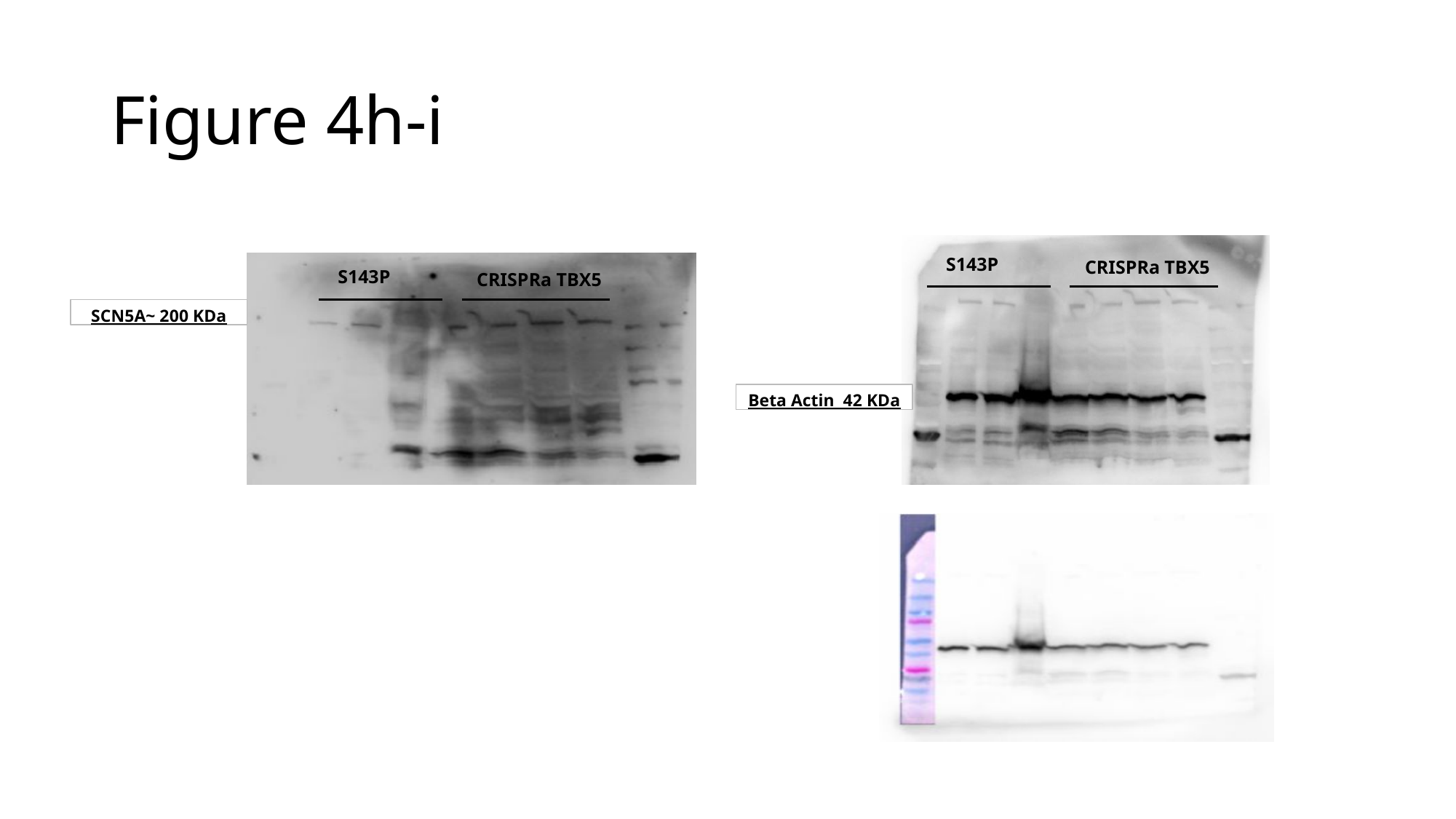

# Figure 4h-i
S143P
CRISPRa TBX5
S143P
CRISPRa TBX5
SCN5A~ 200 KDa
Beta Actin 42 KDa

## Slide 5
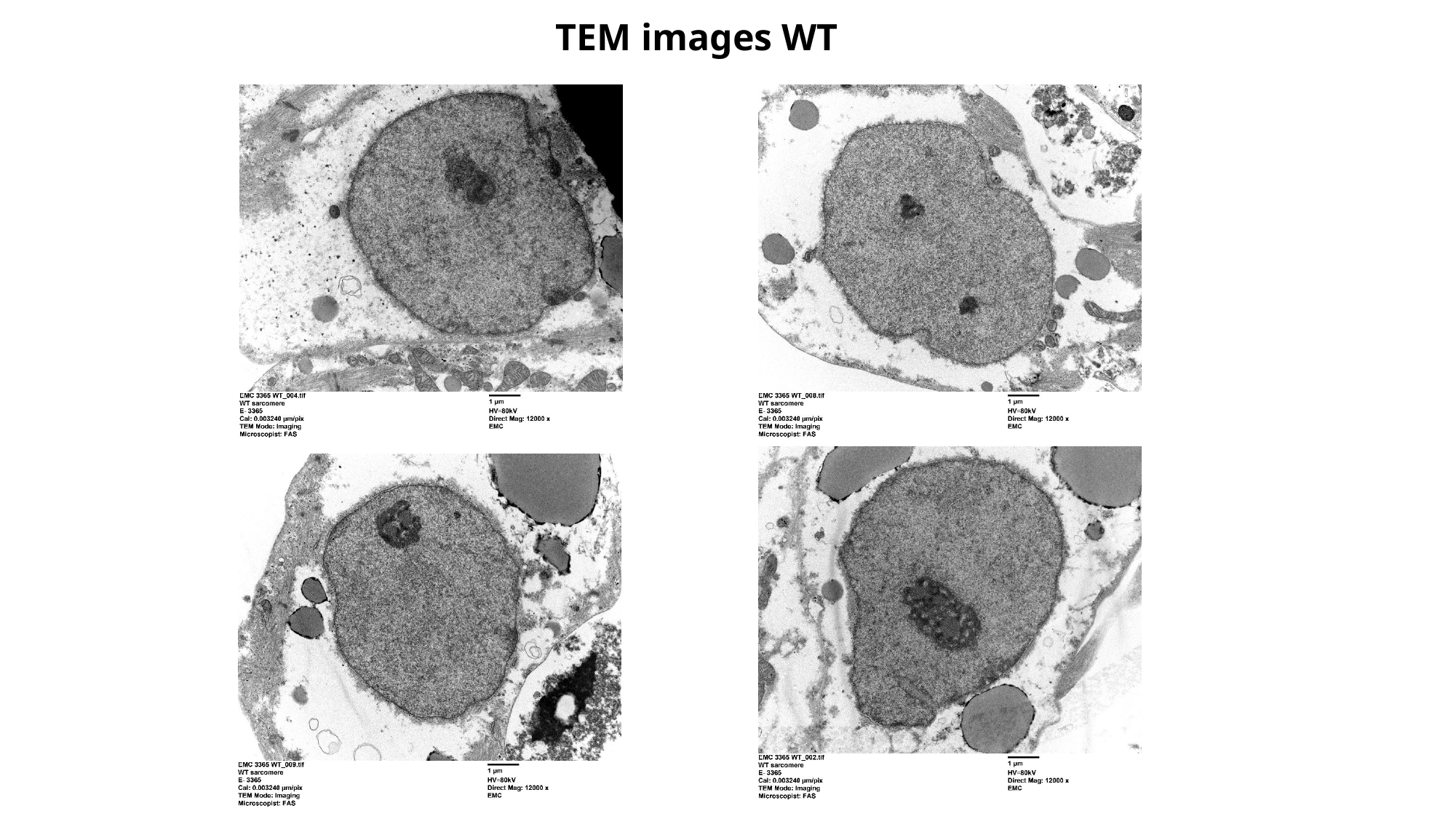

# TEM images WT

## Slide 6
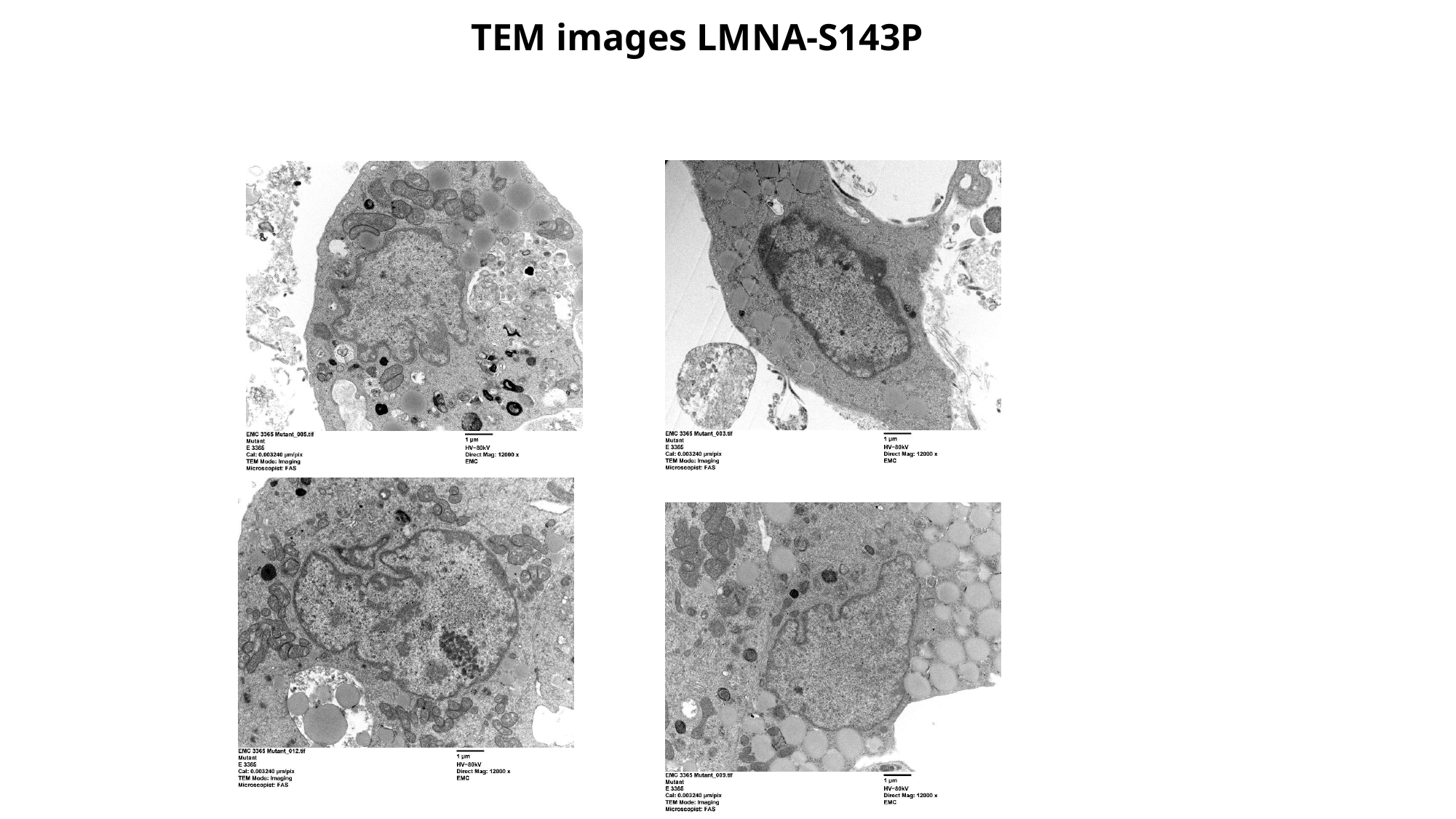

TEM images LMNA-S143P
