# Supplementary material for: Gene-gene interactions between a LMNA variant and common polymorphisms drive early-onset atrial fibrillation
Source: Nat Commun. 2026 May 19;17:6594. doi: 10.1038/s41467-026-73113-0 (PMC13381584; doi:10.1038/s41467-026-73113-0)
Supplement: Supplementary file 4 — Transparent Peer Review file [file 41467_2026_73113_MOESM4_ESM.pdf]

nature portfolio

Peer Review File

Gene-Gene Interactions Between A LMNA Variant and Common Polymorphisms Drive Early-Onset Atrial Fibrillation

Corresponding Author: Dr Dawood Darbar

This file contains all reviewer reports in order by version, followed by all author rebuttals in order by version.

A version of this paper was originally rejected for publication by Nature Communications, however that decision was reconsidered after appeal by the authors.

Version 0:

Reviewer comments:

Reviewer #1

(Remarks to the Author)
The authors hypothesize a previously recognized phenomenon whereby a rare variant may promote the phenotype of AF by interacting with a common polymorphism, as highlighted in the title. The main component of this paper in regards to novelty is the proposed interaction between a rare LMNA variant and a common SNP. The additional data related to PRS and rare variants is not novel in this field.

The data presented is broad and complex. I would like to start by addressing important initial concepts related to study design and conclusions.

1. The rare variant is clear. But what is the common variant involved here? Does the patient actually carry the SCN10A common SNP previously reported to regulate INa?
2. Related to above, did all affected patients harbor this SCN10A SNP believed to interact with the rare LMNA variant? This is relevant, if it is proposed this interaction is the cause of AF.
3. I am very concerned by the choice of WT iPSC CMs by which all of the data is thereafter derived.
It appears the WT iPSC were not isogenic cell lines for the chromatin assembly and gene expression studies, leading to thousands of differences between patient and WT cells. This cannot possibly all be due to the LMNA variant and is most likely due to the fact these these comparison groups from 2 different people harboring a large amount of genetic differences. The appropriate control group for the initial studies would be the same patient...using isogenic cell lines as the control.
4. Have the authors assessed other LMNA variants to ensure their proposed epistatic interaction with SCN5A, indirectly through SCN10A, is consistent? Or do you propose this is specific to S143P only?
5. Assuming the authors perform the initial chromatin assembly and gene expression studies using the appropriate control, do other LMNA S143P patient iPSC's show consistent results?
6. Is any blinding occurring between patient and control cells for the scientist comparing assay results?

Reviewer #2

(Remarks to the Author)
This study links a pathogenic variant in LMNA to regulation of gene expression and epigenetic state in atrial-like cardiomyocytes derived from an iPSC line, then focuses on the reduced expression of the SCN5A gene and sodium current/conduction in iPSC-derived atrial-like cardiomyocyte model, and shows that the reduced sodium current/conduction can be rescued by increasing SCN5A expression in the cell model. The authors claim that this the first molecular evidence of epistatic gene-gene interactions driving arrhythmia risk, and mechanistically links atrial myopathy and AF.

The claim of first molecular evidence is unfounded: Several published studies have shown such epistatic interactions in atrial arrhythmogenesis (e.g. PMID: 38189150 (Tbx5, Zfpm2); PMID: 34289528 (PDE4DIP; DES); PMID: 22402074; PMID: 31609246 (Tbx5, Pln); PMID: 27582060 (Tbx5, Pitx2); PMID: 36715501 (Tbx5, Prrx1)).
Furthermore, the epistatic interaction reported here is limited to one LMNA variant causing pleiotropic effects and one of many deregulated downstream effector genes (SCN5A) which in a dedicated cell model rescues one of the effects. In other

words, from >8000 deregulated genes in the LMNA-S143P variant cell line, the authors select the downregulated gene that is known to be rate-limiting for sodium current and conduction in the myocardium, SCN5A (while largely ignoring all others). They then go on to show that sodium current and conduction are indeed reduced in this atrial cell model, and that when SCN5A is induced in the cell model, either by CRISPRa activation of the promoter or the previously established SCN5A enhancer, sodium current and conduction are increased. Taken together, this appears to be an analysis biased towards the outcome that was predicted.

Specific comments
1) In their differential gene expression analysis and chromatin accessibility profiling, the authors compare atrial CMs derived from only 1 line with the LMNA variant and 1 control line, while it is known that line-to-line and differentiation to differentiation differences account for a large fraction of variation. Moreover, they compared an LMNA-S143P iPSC line with a WT line (the genetic relationship between the two lines is not clear from the manuscript). Different genetic backgrounds (assuming they are of different genetic background) further contribute to transcriptome and accessibility differences which cannot be discriminated from the effects of the LMNA variant. Given the central role of these data sets in this study and its claims, the transcriptome/accessibility data should be much more solid, and obtained from multiple independent cell lines, isogenic controls (the authors actually made such a line but did not use it), and differentiation batches to uncover the LMNA-variant-dependent effects on transcription and accessibility.

2) Data analysis: Figure 2g shows correlation between ATAC seq and RNAseq data, but the authors only show positively correlating data, leaving out the negatively correlating data. Then, they show correlation of the data sets, including a statistical test, which, of course, is positive and significant because the negatively correlating data has been left out. Figure 3a same.

3) What is the difference between expression data shown in figure 3c and 3h?

4) Differences in accessibility in Figure 3d-g and 4a-d are difficult to see and do not seem to support the claimed changes in all cases. More importantly, they should be quantified.
Furthermore, the authors assume the accessible sites they point out are enhancers of the target genes they focus on. The assumption is made that H3K27ac signal and accessibility demarcate enhancers and that interactions between H3K27ac-enriched genomic sequences and promoters captured by published PC-HiC data shows that these enhancers regulate such promoters. However: 1) degree of accessibility does not correlate well with enhancer or promoter activity. 2) Neither H3K27ac nor accessibility specifically demarcate enhancers (only a fraction of the accessible sites functions as enhancer). 3) a large fraction of the interactions seen in (PC) HiC is structural, not regulatory. Therefore, observed interactions should not be interpreted as functional enhancer-promoter interactions. 4) the PC HiC data is not saturating, many interactions are missed.
The authors do not use data or even refer to studies in which efforts have been made to try and identify AF-relevant regulatory sequences including enhancers, their target genes and the identification of functional SNPs among the many SNPs in LD with the lead SNP in the association (PMID: 35064145; PMID: 37591828; PMID: 31628324; PMID: 32248749; PMID: 33155827). Their data and analysis should be compared to those data sources and could be improved by integrating them.

5) beta actin in fig1d and fig4 f seem identical, was this taken from the same blot?

6) the authors show that in comparison to the wt line, TBX5 and other TFs essential for driving the (atrial) CM lineage gene programs are downregulated. Whether this is due to LMNA variant remains uncertain (see also comment 1), and it would be important to establish this. But assuming it is, this would then to a large extent explain downregulation of SCN5A (and other CM genes), providing an explanation for the “epistatic interaction” between LMNA and SCN5A. Because TBX5 is an AF associated gene itself that has been well studied in the context of atrial CM lineage specification and maintenance, dosage dependent mechanisms of action, AF and SCN5A regulation, this then sheds a different light on the conclusions. If LMNA indeed pleiotropically disrupts the transcription factor network regulating atrial CM gene expression, then any effector gene regulating by the key transcription factors of this network will be deregulated. This would also not be specific to AF, but to the overall state of the atrial CMs. This then would be compatible with authors findings, that 8000 genes are deregulated and 45 k sites show differential accessibility.

7) The authors took all AF risk loci from recent GWAS studies (how many?) and find that 8 loci overlap with their altered accessibility regions. Please specify “loci” and “regions”. It is not clear whether this is a significant finding, i.e. whether this is statistically enriched overlap. Of note, most AF risk loci are found in CM lineage relevant gene regions, and the differential accessibility data set the authors used to overlap with AF GWAS variants is derived from CMs. Moreover, it is not clear whether the authors focused on the lead SNP, which may not be functional, or all SNPs in LD with the lead SNP (a few of which may be functional). This also affects the assignment of relevant variants, assignment of enhancers (see above), their colocalization, the assignment of enhancers to target genes (see also above). Overall, many assumptions are made regarding variant enhancers of target genes, which however should be demonstrated using functional assays or supported by published functional data.

8) The rs6801957 the authors focus on was indeed be found to disrupt an enhancer of SCN5A, but later was reported to only mildly affect SCN5A expression when disrupted, but cause the downregulation of a cardiac-specific short isoform of SCN10A driven from an intronic promoter close by (PMID: 33910361). This caused reduced sodium current. These observations impact on the explanations and conclusions of the current manuscript and should be taken into account.

9) the section about “Risk of Incident AF in carriers of LMNA protein-altering...” is not clear to me. Is the effect of PRS and PAVs synergistic, additive, less than additive? How does the information regarding a patients PRS and PAVs contribute to risk assessment?

Reviewer #3

(Remarks to the Author)
In this study, authors hypothesized that a rare LMNA variant may alter chromatin accessibility and gene expression at AF-risk loci. They conducted chromatin accessibility and gene expression profiling in iPSC-aCMs generated from a proband carrying the LMNA-S143P variant. This analysis identified significantly altered chromatin accessibility at eight AF associated loci, including a SNP within a known SCN5A enhancer. SCN5A expression and INa were consistently reduced, impairing atrial conduction and promoting re-entry. They employed CRISPR-based approaches to correct the LMNA-S143P variant, activate the SCN5A promoter, and enhance SCN10A regulatory activity. These interventions successfully restored SCN5A expression and INa. They applied a PRS for AF to carriers of rare LMNA PAVs in the UK Biobank and All of Us (AoU) cohorts. Individuals with a high PRS exhibited a two-fold increased risk of early-onset AF compared to those with a lower PRS emphasizing the need to integrate rare and common genetic variations for improved AF risk stratification. They highlighted the need for comprehensive genetic assessment, incorporating both rare and common variants, not only for risk prediction but also for early intervention to prevent atrial myopathy and its complications. This is an interesting manuscript, however, several concerns need to be addressed to enhance the importance of this study.

1) Please indicate the age at which each of the following episodes was observed.
▪ - underwent implantation of a biventricular pacemaker (Page 5, lines 16-17)
▪ - required pacemakers, (Page 6, line 1)
▪ - underwent heart transplantation due to CHF (Page 6, line 2)

2) Two main arrhythmogenic mechanisms including atrial reentry and ectopic atrial activity are considered to lead to AF. Please provide evidence or a detailed discussion on how the following findings are involved in the development of AF. Furthermore, discuss and describe which finding has the greatest impact on the development of AF.
▪ Structural genes associated with early onset-AF such as MYL4, TTN, MYBPC3, DSP and PKP2 were significantly downregulated.
▪ Gene enrichment identified altered cardiovascular signaling pathways, including upregulation of DCM, β-adrenergic signaling.
▪ Downregulation of the cardiac hypertrophy and differentiation pathways.
▪ The DNA damage repair pathway was upregulated
▪ Reduced accessibility at enhancers of genes encoding transcription factors, TBX5, GATA4, GATA6, and the atrial-specific gene, NPPA
▪ CEP68 was upregulated
▪ NPTN was significantly downregulated
▪ SGK1 was significantly downregulated
▪ Calcium transient measurements revealed that LMNA-S143P iPSC-aCMs exhibited reduced transient amplitude and duration and irregular transient intervals

Version 1:

Reviewer comments:

Reviewer #1

(Remarks to the Author)
The responses and additional experiments from the authors is appreciated.

The specific mechanisms being proposed would be more solidified by having demonstrated similarities, at least in genotype data at AF regulatory loci, for all 4 cases in the original family, particularly at the SCN10A regulatory locus which is a focus of this paper. Also, would have been enhanced by UK biobank data of LMNA-AF cases (rather than PRS in general).
The addition of a second mutation is useful, but this is limited to showing downregulation of SCN5A and TBX5, rather than genotype data at AF regulatory loci.

The entire focus on the SCN10A locus comes from the proband (1/4 cases). The UK Biobank data focuses on PRS, rather than LMNA-AF and the SCN10A locus. This disconnect is curious.

Reviewer #2

(Remarks to the Author)
The authors have addressed my concerns, provided additional data that strengthen their interpretations and conclusions. The revised manuscript is greatly improved.

Reviewer #3

(Remarks to the Author)
In this revised version of the manuscript, authors modified the content according to reviewer's comments. The revision has been done well, and I have no further points.

Open Access This Peer Review File is licensed under a Creative Commons Attribution 4.0 International License, which permits use, sharing, adaptation, distribution and reproduction in any medium or format, as long as you give appropriate credit to the original author(s) and the source, provide a link to the Creative Commons license, and indicate if changes were made.
In cases where reviewers are anonymous, credit should be given to 'Anonymous Referee' and the source.
The images or other third party material in this Peer Review File are included in the article’s Creative Commons license, unless indicated otherwise in a credit line to the material. If material is not included in the article’s Creative Commons license and your intended use is not permitted by statutory regulation or exceeds the permitted use, you will need to obtain permission directly from the copyright holder.
To view a copy of this license, visit https://creativecommons.org/licenses/by/4.0/

Response to Reviewers' Comments: Gene-Gene Interactions Between a *LMNA* Variant and Common Polymorphisms Drive Early-Onset Atrial Fibrillation

Dear Referees,

We are sincerely grateful for the thoughtful and constructive feedback on our manuscript titled ***"Gene-Gene Interactions Between a LMNA Variant and Common Polymorphisms Drive Early-Onset Atrial Fibrillation."*** The critiques have been invaluable in strengthening both the rigor and clarity of our work.

In our revised submission, we have undertaken substantial revisions that directly address the central methodological and interpretative concerns outlined in the decision letter. Specifically, we have:

1. **Addressed the methodological concern regarding non-isogenic controls**: We have performed transcriptomic and chromatin accessibility analyses using CRISPR-corrected isogenic iPSC-derived atrial cardiomyocytes (iPSC-aCMs) from the *LMNA* p.S143P patient. This approach eliminates confounding due to inter-individual genetic variation. Importantly, the transcriptomic and epigenomic alterations observed in the isogenic comparison closely mirror those observed in the original dataset, confirming that these effects are attributable to the *LMNA* variant. Differentially expressed genes remain enriched for pathways related to cardiomyopathy, chromatin remodeling, and AF pathogenesis, thereby supporting the study's mechanistic conclusions.

2. **Extended validation to additional *LMNA* variant carriers.** We characterized iPSC-aCMs derived from an additional affected family member (the proband's mother) who also carries the *LMNA* p.S143P variant and has AF and conduction disease. These cells recapitulate the key molecular and electrophysiological findings, including reduced *TBX5* and *SCN5A* expression and impaired sodium current. Furthermore, we analyzed iPSC-aCMs from an individual carrying a distinct pathogenic *LMNA* variant p.G449V and observed similar downregulation of *TBX5* and *SCN5A* with associated sodium channel dysfunction (**Fig. 8**), supporting the generalizability of the mechanism across *LMNA* genotypes.

3. **Strengthened evidence for gene–gene interactions at AF loci**: While rare-common variant interactions have previously been reported, the revised manuscript now includes functional validation of two additional AF-associated regulatory elements harboring rs2359171 (*ZFHX3)* and rs12640611 (*WDR1)*, identified through chromatin accessibility analysis. Using CRISPR activation/interference (a/i), we show that perturbing these enhancers alters expression of their respective target genes, providing functional evidence that *LMNA*-driven chromatin remodeling can modify the regulatory effects of common AF risk variants beyond the previously characterized *SCN10A–SCN5A* locus.

4. **Expanded integration with external genomic datasets:** We incorporated human cardiac eQTL data and prior functional prioritization studies to refine SNP-gene assignments. These analyses, detailed in a new supplementary table (**Table S11**), support a broader model in which *LMNA* variants modulate the regulatory landscape at multiple AF loci.

5. **Clarified the mechanistic framework and refined statements of novelty:** We have reframed the manuscript to avoid overstating novelty and instead emphasize the central mechanistic advance: pathogenic LMNA-mediated nuclear envelope dysfunction leads to chromatin reorganization that reshapes atrial gene regulatory networks and modifies the

regulatory impact of common AF risk variants. By integrating isogenic epigenomic analyses, patient-derived cellular phenotyping, and locus-specific functional interrogation, , our findings link upstream chromatin dysregulation to downstream atrial myopathy and conduction abnormalities. This framework advances understanding of how rare and common genetic variation converge on shared regulatory networks to drive AF.

Collectively, these revisions substantially strengthen the methodological rigor and mechanistic understanding of *LMNA*-mediated AF and address the concerns that previously precluded publication.

The revised manuscript begins with a concise summary of major changes and is accompanied by a detailed, point-by-point response to each reviewer's comment. Reviewer comments are presented in ***bold italics***, followed by our responses and excerpts from the revised text (highlighted in **red**).

We respectfully request reconsideration of our revised manuscript for publication in *Nature Communications*.

Sincerely,

Dawood Darbar

Reviewer#1:

The authors hypothesize a previously recognized phenomenon in which a rare variant may promote the AF phenotype by interacting with a common polymorphism, as highlighted in the title. The main component of this paper regarding novelty is the proposed interaction between a rare LMNA variant and a common SNP. The additional data related to PRS and rare variants are not novel in this field. The data presented is broad and complex. I want to start by addressing important initial concepts related to study design and conclusions.

Response: We thank the reviewer for their thoughtful evaluation and for highlighting the central focus of our study, the interaction between a rare *LMNA* variant and common SNPs in contributing to AF pathogenesis. While the concept of rare-common variant interaction is recognized, our work provides novel mechanistic insights into how a specific rare variant (*LMNA* p.S143P) modulates chromatin accessibility at multiple AF-associated risk loci. Using a patient-specific iPSC-aCM model, we demonstrate that the *LMNA* variant leads to epigenetic remodeling at regulatory regions harboring common AF risk SNPs, thereby providing a molecular framework for the observed gene-gene interactions. To further strengthen this link, we examined the allelic configuration at these loci and integrated data from prior studies to support the functional relevance of the implicated SNPs and their target genes. Notably, we also employed CRISPR activation (a) and inhibition (a/i) to functionally validate two additional AF risk loci. This analysis identified enhancers in *WDR1*, which plays a critical role in actin dynamics and cytoskeletal organization, and in *ZFHX3*, a well-known transcription factor implicated in AF pathogenesis (**Fig. 6**). To our knowledge, this is the first direct experimental validation of the downstream targets of these AF variant regions using CRISPRa/i in a human atrial model. We hope these additional mechanistic insights clarify the novelty and impact of our study in AF genetics.

Specific Comments

1. ***The rare variant is clear. But what is the standard variant involved here? Does the patient actually carry the SCN10A common SNP previously reported to regulate INa?***
2. ***Related to above, did all affected patients harbor this SCN10A SNP believed to interact with the rare LMNA variant? This is relevant if it is proposed that this interaction is the cause of AF.***

Response: We thank the reviewer for these critical questions regarding the specific common variants involved in the proposed gene-gene interaction model.

1. We confirm that the patient carrying the *LMNA* p.S143P variant is homozygous for the risk allele at the intronic *SCN10A* SNP previously reported to influence *SCN5A* expression and I_{Na} regulation. This supports the plausibility of a functional interaction between the rare *LMNA* variant and a common regulatory variant at the *SCN10A-SCN5A* locus.
2. In addition to the *SCN10A* SNP, we performed genotype analysis at other AF-associated SNPs located within regulatory regions that show differential chromatin accessibility in *LMNA* p.S143P iPSC-aCMs. Notably, the patient carries the risk allele at 6 out of 8 such loci with altered accessibility (**Fig. 5, Table S9**). Risk allele annotation was based on the most recent cross-ancestry meta-analyses of AF GWAS data.

While our study focuses on a single proband and provides mechanistic validation in a patient-specific model, these findings support the hypothesis that the rare *LMNA* variant may act as a chromatin modifier, unmasking the regulatory effects of common AF risk alleles. We agree that extending this analysis to a larger patient cohort in future studies will be critical to further validate the prevalence and functional impact of such interactions[1].

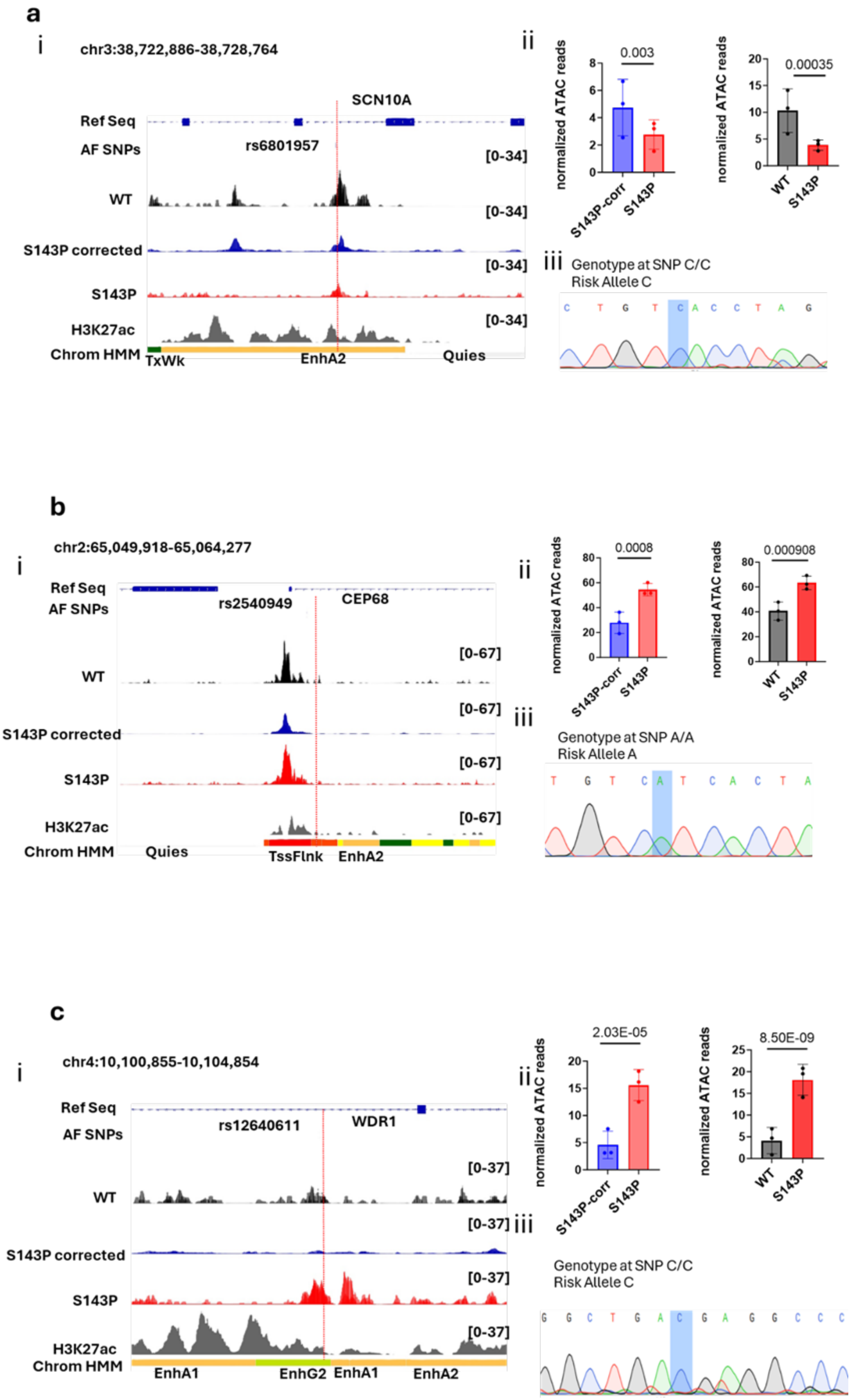

a
i
chr3:38,722,886-38,728,764
SCN10A
Ref Seq
AF SNPs
rs6801957
[0-34]
WT
[0-34]
S143P corrected
[0-34]
S143P
[0-34]
H3K27ac
Chrom HMM
TxWk
EnhA2
Quies
ii
normalized ATAC reads
0.003
S143P-corr
S143P
0.00035
WT
S143P
iii
Genotype at SNP C/C
Risk Allele C
b
i
chr2:65,049,918-65,064,277
Ref Seq
AF SNPs
rs2540949
CEP68
[0-67]
WT
[0-67]
S143P corrected
[0-67]
S143P
[0-67]
H3K27ac
Chrom HMM
Quies
TssFlnk
EnhA2
ii
normalized ATAC reads
0.0008
S143P-corr
S143P
0.000908
WT
S143P
iii
Genotype at SNP A/A
Risk Allele A
c
i
chr4:10,100,855-10,104,854
Ref Seq
AF SNPs
rs12640611
WDR1
[0-37]
WT
[0-37]
S143P corrected
[0-37]
S143P
[0-37]
H3K27ac
Chrom HMM
EnhA1
EnhG2
EnhA1
EnhA2
ii
normalized ATAC reads
2.03E-05
S143P-corr
S143P
8.50E-09
WT
S143P
iii
Genotype at SNP C/C
Risk Allele C

d

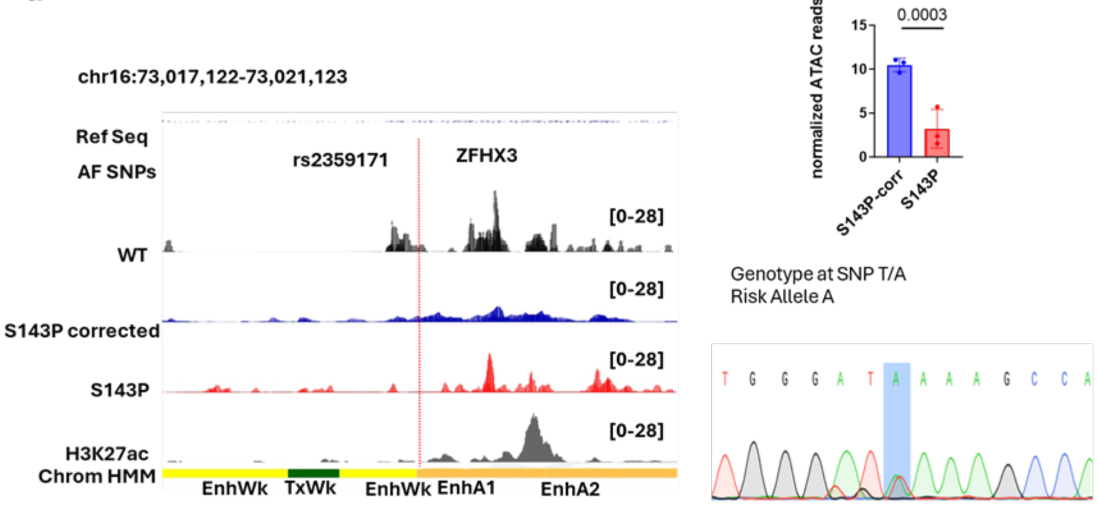
chr16:73,017,122-73,021,123
Ref Seq
AF SNPs
rs2359171
ZFHX3
[0-28]
WT
[0-28]
S143P corrected
[0-28]
S143P
[0-28]
H3K27ac
Chrom HMM
EnhWk
TxWk
EnhWk
EnhA1
EnhA2
normalized ATAC reads
0.0003
15
10
5
0
S143P-corr
S143P
Genotype at SNP T/A
Risk Allele A
T G G G A T A A A A G C C A

e

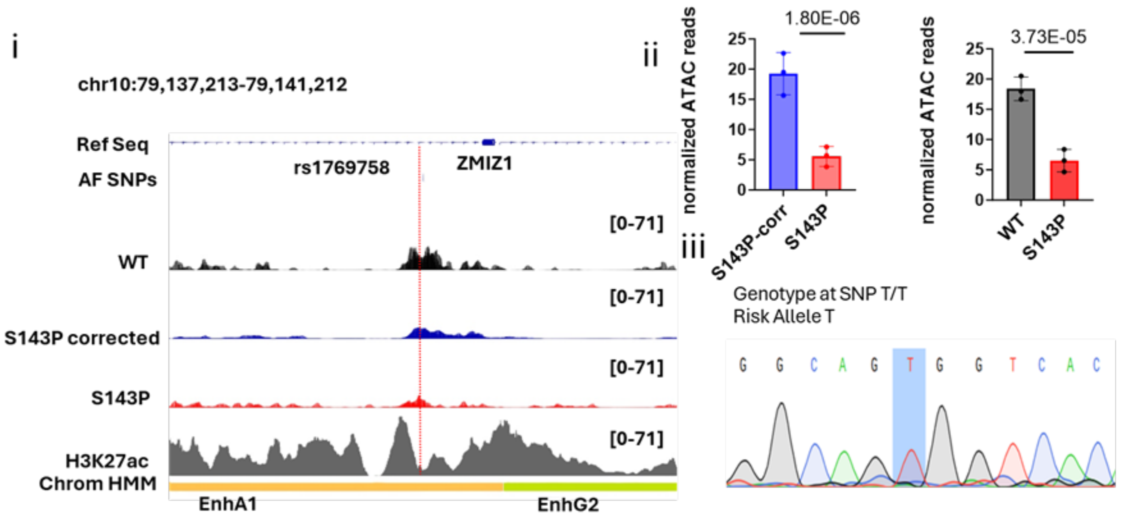
i
chr10:79,137,213-79,141,212
Ref Seq
AF SNPs
rs1769758
ZMIZ1
[0-71]
WT
[0-71]
S143P corrected
[0-71]
S143P
[0-71]
H3K27ac
Chrom HMM
EnhA1
EnhG2
ii
normalized ATAC reads
1.80E-06
25
20
15
10
5
0
S143P-corr
S143P
normalized ATAC reads
3.73E-05
25
20
15
10
5
0
WT
S143P
iii
Genotype at SNP T/T
Risk Allele T
G G C A G T G G T C A C

f

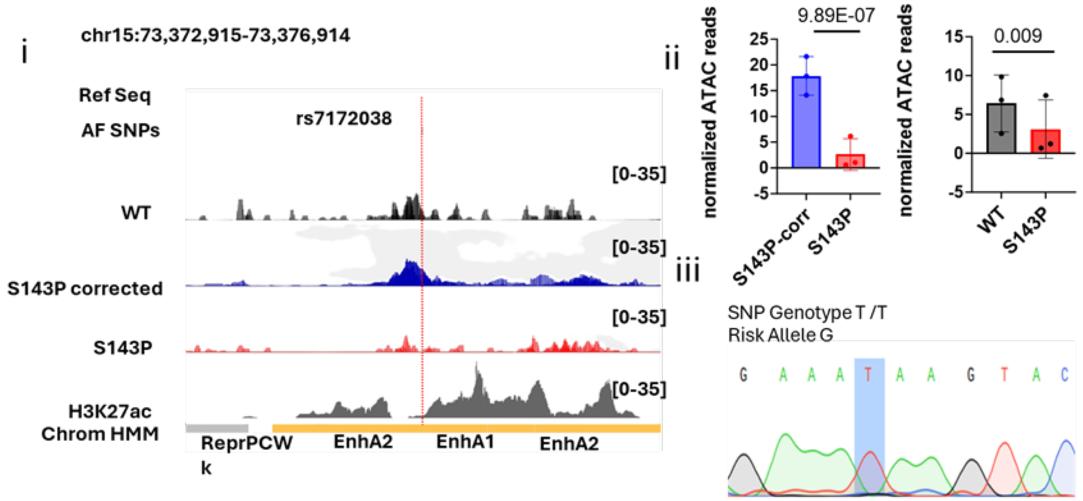
i
chr15:73,372,915-73,376,914
Ref Seq
AF SNPs
rs7172038
[0-35]
WT
[0-35]
S143P corrected
[0-35]
S143P
[0-35]
H3K27ac
Chrom HMM
ReprPCW
k
EnhA2
EnhA1
EnhA2
ii
normalized ATAC reads
9.89E-07
25
20
15
10
5
0
-5
S143P-corr
S143P
normalized ATAC reads
0.009
15
10
5
0
-5
WT
S143P
iii
SNP Genotype T /T
Risk Allele G
G A A A T A A G T A C

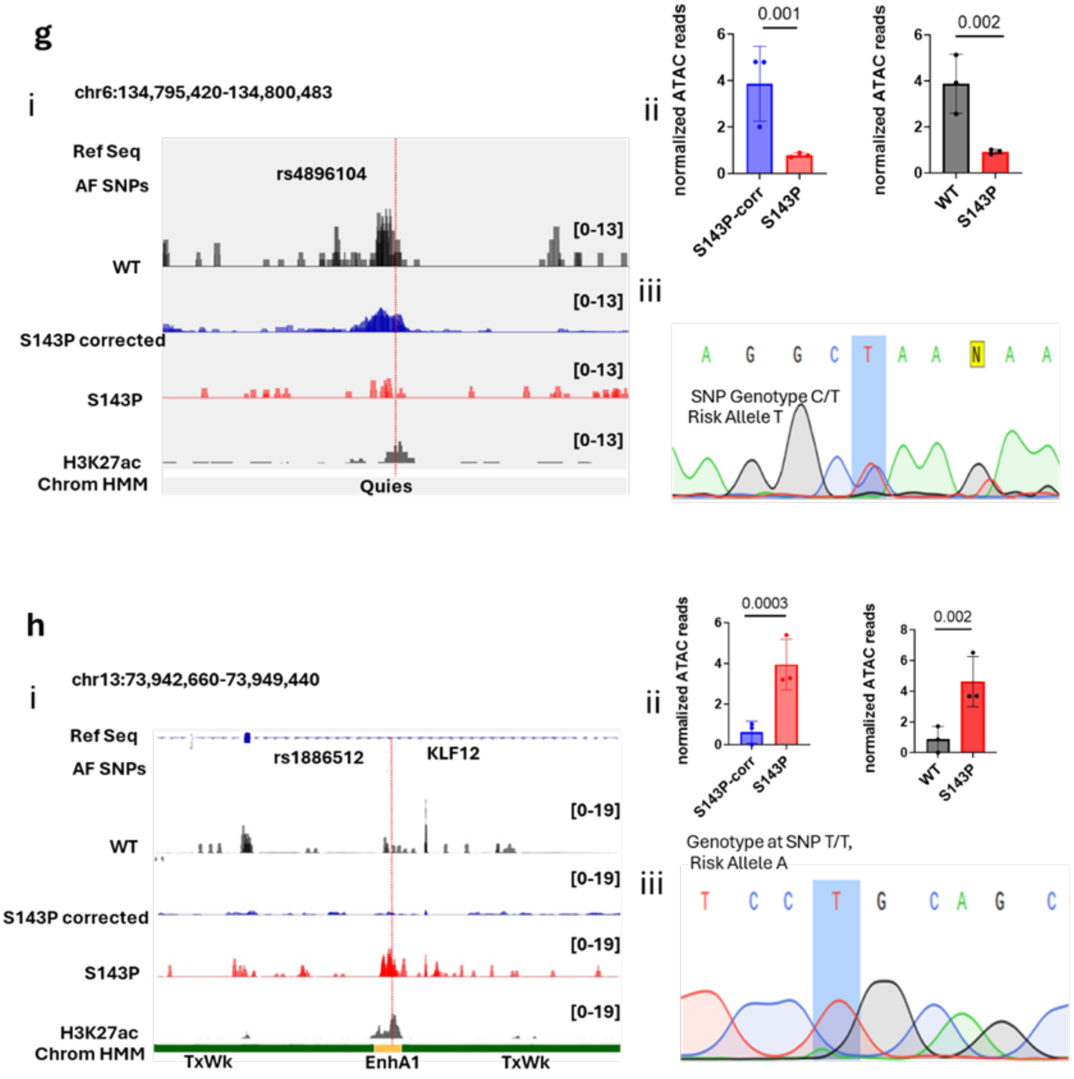

ChromHMM State Descriptions

State description	Abbrev.
Active TSS	TssA
Flanking TSS	TssFlnk
Flanking TSS upstream	TssFlnkU
Flanking TSS downstream	TssFlnkD
Strong transcription	Tx
Weak transcription	TxWk
Genic enhancer 1	EnhG1
Genic enhancer 2	EnhG2
Active enhancer 1	EnhA1
Active enhancer 2	EnhA2
Weak enhancer	EnhWk
ZNF genes & repeats	ZNF/Rpts
Heterochromatin	Het
Bivalent/poised TSS	TssBiv
Bivalent enhancer	EnhBiv
Repressed Polycomb	ReprPC
Weak repressed Polycomb	ReprPCWk
Quiescent/low	Quies

Fig. 5: Altered chromatin accessibility at regulatory regions harboring AF-associated SNPs in *LMNA* p.S143P iPSC-aCMs. Integrated genome viewer ATAC-seq track plots, normalized ATAC reads, and proband genotype at differentially accessible regions with AF-associated SNPs located in regulatory regions marked by H3K27ac and ChromHMM annotations. **a.** rs6801957**. b.** rs4896104. **c.** rs7172038**. d.** rs2540949. **e.** rs1769758. **f.** rs1886512. **g.** rs2359171**. h.** rs12640611**.** H3K27ac ChIP-seq track from human atrial tissue. The dashed line shows the location of the SNP. Genomic location according to GR Ch38/hg38.

Table S9: Proband's genotype at AF SNPs in differentially accessible regions.

SNP	Mapped Gene	Proband's Genotype	Risk Allele
rs6801957	SCN5A/SCN10A	C/C	C
rs2540949	CEP68	A/A	A
rs2359171	ZFHX3	T/A	A
rs1769758	ZMIZ1	T/T	T
rs12640611	WDR1, SLC2A9	C/C	C
rs4896104	ALDH8A1, SGK1	C/T	T
rs1886512	KLF12	T/T	A
rs7172038	REC114, HCN4	T	G

3. I am very concerned by the choice of WT iPSC CMs by which all of the data is thereafter derived. It appears the WT iPSCs were not isogenic cell lines for the chromatin assembly and gene expression studies, leading to thousands of differences between patient and WT cells. This cannot possibly all be due to the LMNA variant and is most likely due to the fact that these comparison groups from 2 different people harbor a large amount of genetic differences. The appropriate control group for the initial studies would be the same patient...using isogenic cell lines as the control.

Response: We fully agree with the reviewer that the use of isogenic controls is essential to isolate the effect of the *LMNA* p.S143P variant from background genetic differences. In response to this concern, and to ensure the validity of our mechanistic conclusions, we have now performed both transcriptomic and chromatin accessibility analyses using CRISPR-corrected isogenic iPSC-aCMs derived from the same patient**.** As shown in **Fig. 3** and detailed in our revised results, we observe a highly consistent pattern of differentially expressed genes and chromatin accessibility changes in the isogenic comparison, confirming that these alterations are driven explicitly by the *LMNA* p.S143P variant and not by inter-individual variability. The pathways enriched in the isogenic analysis include those related to chromatin remodeling, cardiac muscle function, and AF pathogenesis, further strengthening the mechanistic link (**Fig. 3**). These new experiments now serve as the primary basis for our mechanistic conclusions. At the same time, data from the WT iPSC-aCMs are presented only as supporting validation. We believe this addresses the reviewer's concern and significantly improves the study's rigor.

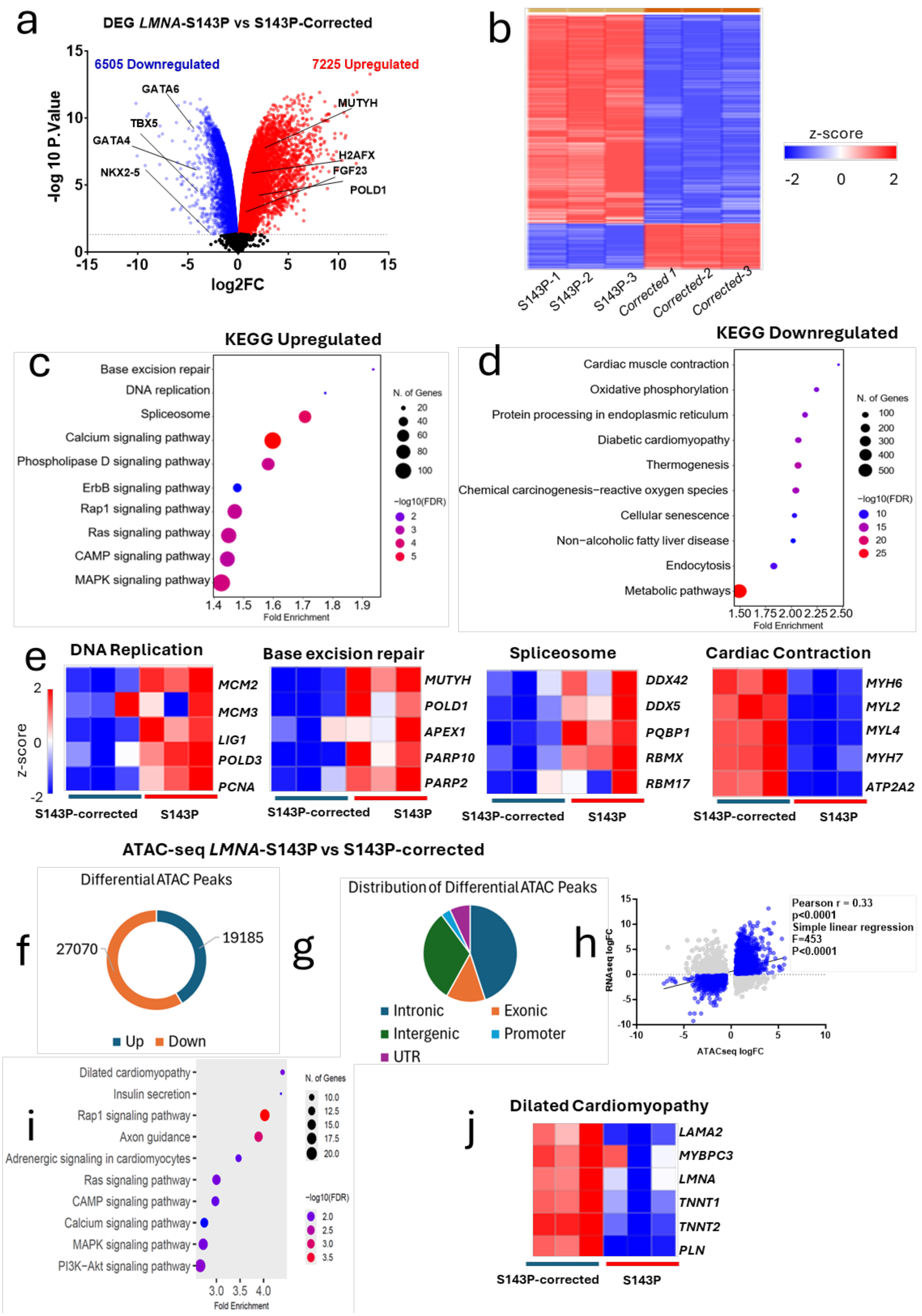

a
DEG LMNA-S143P vs S143P-Corrected
6505 Downregulated
7225 Upregulated
-log 10 P.Value
log2FC
GATA6
TBX5
GATA4
NKX2-5
MUTYH
H2AFX
FGF23
POLD1
b
z-score
S143P-1
S143P-2
S143P-3
Corrected 1
Corrected-2
Corrected-3
KEGG Upregulated
c
Base excision repair
DNA replication
Spliceosome
Calcium signaling pathway
Phospholipase D signaling pathway
ErbB signaling pathway
Rap1 signaling pathway
Ras signaling pathway
CAMP signaling pathway
MAPK signaling pathway
N. of Genes
−log10(FDR)
Fold Enrichment
KEGG Downregulated
d
Cardiac muscle contraction
Oxidative phosphorylation
Protein processing in endoplasmic reticulum
Diabetic cardiomyopathy
Thermogenesis
Chemical carcinogenesis−reactive oxygen species
Cellular senescence
Non−alcoholic fatty liver disease
Endocytosis
Metabolic pathways
e
DNA Replication
MCM2
MCM3
LIG1
POLD3
PCNA
Base excision repair
MUTYH
POLD1
APEX1
PARP10
PARP2
Spliceosome
DDX42
DDX5
PQBP1
RBMX
RBM17
Cardiac Contraction
MYH6
MYL2
MYL4
MYH7
ATP2A2
S143P-corrected
S143P
ATAC-seq LMNA-S143P vs S143P-corrected
f
Differential ATAC Peaks
27070
19185
Up
Down
g
Distribution of Differential ATAC Peaks
Intronic
Exonic
Intergenic
Promoter
UTR
h
Pearson r = 0.33
p<0.0001
Simple linear regression
F=453
P<0.0001
RNAseq logFC
ATACseq logFC
i
Dilated cardiomyopathy
Insulin secretion
Rap1 signaling pathway
Axon guidance
Adrenergic signaling in cardiomyocytes
Ras signaling pathway
CAMP signaling pathway
Calcium signaling pathway
MAPK signaling pathway
PI3K−Akt signaling pathway
j
Dilated Cardiomyopathy
LAMA2
MYBPC3
LMNA
TNNT1
TNNT2
PLN

Fig. 3: Transcriptomic and chromatin accessibility changes in *LMNA* p.S143P iPSC-aCMs. **a.** Volcano plot showing differentially expressed genes between *LMNA* p.S143P and CRISPR-corrected isogenic iPSC-aCMs. Significantly upregulated and downregulated genes are highlighted**. b.** Clustering heatmap of RNA-seq showing differentially expressed genes (DEGs) in *LMNA* p.S143P iPSC-aCMs. **c-d.** KEGG pathway enrichment analysis of differentially expressed genes. Upregulated genes are enriched in pathways related to chromatin organization and remodeling, while downregulated genes are associated with cardiac muscle contraction, metabolic dysregulation, and cellular senescence**. e.** DEGs in the top enriched pathways **f.** Differential ATAC-seq peaks in *LMNA* p.S143P iPSC-aCMs compared to CRISPR-corrected isogenic controls, indicating widespread changes in chromatin accessibility. **g.** *LMNA* p.S143P preferentially alters chromatin accessibility in non-coding regions, including potential regulatory elements such as enhancers and promoters. **h**. correlation of promoter accessibility and DEG in *LMNA*-S13P iPSC-aCMs**. i**. KEGG pathway enrichment analysis of genes with altered promoter accessibility identifies dilated cardiomyopathy as the top associated pathway, linking chromatin remodeling to the pathogenesis of cardiac disease. **j.** Gene expression alterations in the dilated cardiomyopathy pathway.

4. Have the authors assessed other LMNA variants to ensure their proposed epistatic interaction with SCN5A, indirectly through SCN10A, is consistent? Or do you propose this is specific to S143P only?

<u>Response</u>: We thank the reviewer for raising this important question regarding the broader applicability of the proposed epistatic interaction.

To address this directly, we extended our analysis beyond the *LMNA* p.S143P variant. In addition to the proband, we recruited an individual harboring a distinct, de novo heterozygous pathogenic variant, *LMNA* c.1346G>T (p.Gly449Val), associated with cardiac disease and muscular dystrophy. iPSC-aCMs generated from this individual demonstrated reduced expression of *TBX5* and *SCN5A*, consistent with our findings in *LMNA* p.S143P (**Fig. 8**). Collectively, these observations support the possibility that dysregulation of atrial transcriptional networks and sodium channel expression is not confined to the S143P variant and may occur across multiple pathogenic *LMNA* genotypes.

Results (<u>page 17, lines 382-395</u>):

> "*Downregulation of SCN5A and TBX5 in LMNA-G449V iPSC-aCMs:* To determine whether additional LMNA variants share similar molecular mechanisms underlying AF susceptibility, we generated iPSC-aCMs from an individual harboring a de novo, heterozygous, pathogenic variant, LMNA c.1346G>T p.Gly449Val, associated with cardiac disease and muscular dystrophy (Fig. 8a-c). Her cardiac phenotype included recurrent atrial tachycardia, premature atrial contractions, sinus pauses, and intermittent absence of atrial electrical activity on the EKG. Echocardiography demonstrated mild bi-atrial dilation by echo and mild mitral annular dilation with preserved left ventricular ejection fraction (61-67%) in the absence of guideline-directed medical therapy. Consistent with findings in LMNA p.S143P iPSC-aCMs, we observed downregulation of TBX5 and SCN5A (Fig. 8d-f). Genotyping confirmed heterozygosity for the AF-associated SNP rs6801957. CRISPRa of the corresponding SCN10A intronic enhancer increased SCN5A expression, supporting a conserved regulatory interaction at this locus."

Discussion (<u>page 19-20, lines 442-448</u>):

> "In addition to the S143P variant, we extended our analysis to iPSC-aCMs derived from a distinct variant, LMNA G449V, which also showed a downregulation of TBX5 and SCN5A,

demonstrating that transcriptional dysregulation and sodium channel dysfunction underlie atrial pathology induced by LMNA variants. Collectively, these observations support the possibility that dysregulation of atrial transcriptional networks and sodium channel expression is not confined to the S143P variant and may occur across multiple pathogenic LMNA genotypes."

Prior studies have implicated *SCN5A* dysfunction in *LMNA*-related cardiomyopathy through distinct mechanisms[2-4]. Notably, Brugada syndrome has been reported in a patient with a missense *LMNA* variant, in the absence of pathogenic *SCN5A* variants, suggesting that *LMNA* mutations may influence sodium channel function indirectly, potentially through epigenetic or transcriptional mechanisms[2].

In our model, the proband is homozygous for the AF-associated risk allele at rs6801957**,** within an intronic *SCN10A* enhancer that regulates *SCN5A* expression. ATAC-seq demonstrated reduced chromatin accessibility at this regulatory region in *LMNA* p.S143P iPSC-aCMs, supporting a mechanism in which *LMNA*-mediated chromatin remodeling may amplify the functional impact of common regulatory polymorphisms at the SCN10A-SCN5A locus.

While our most detailed mechanistic analyses were performed in the S143P model, the replication of key transcriptional and electrophysiological findings in an independent pathogenic *LMNA* variant supports the broader relevance of this regulatory mechanism. Further investigation of additional *LMNA* genotypes will be important for defining the extent of this effect.

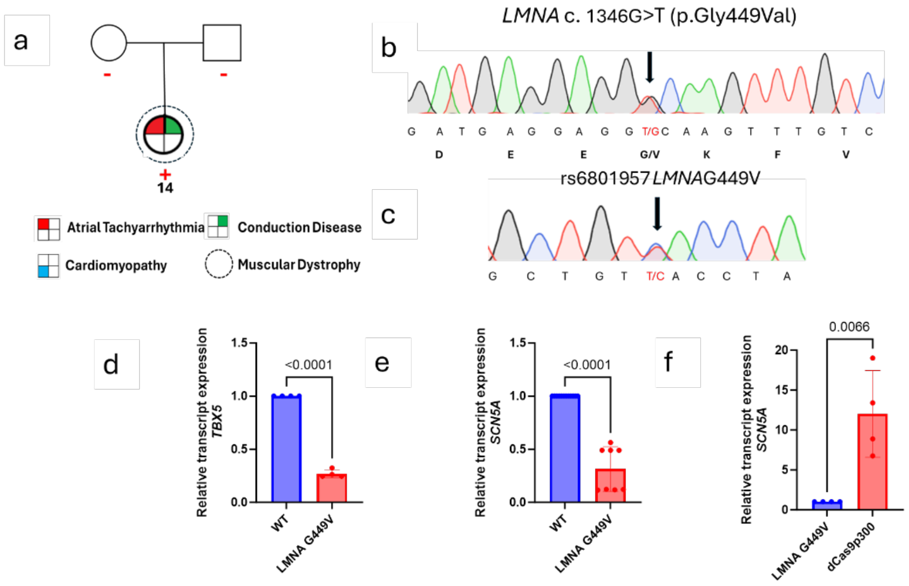

Fig. 8. Sodium channel dysfunction is recapitulated in the *LMNA*-G449V variant. a. Pedigree of proband harboring the *LMNA*-G449V variant. **b.** Sanger sequencing confirms a heterozygous G>T variant. **c.** Presence of one risk allele at the rs6801957 SNP. **d.** Reduction in transcript levels of TBX5 and **e**. *SCN5A*. **f.** CRISPRa of the *SCN10A* enhancer increases the expression of *SCN5A*.

5. Assuming the authors perform the initial chromatin assembly and gene expression studies using the appropriate control, do other LMNA S143P patient iPSCs show consistent results?

Response: As noted above, we have now performed transcriptomic and chromatin accessibility analyses using CRISPR-corrected isogenic iPSCs derived from the same *LMNA* p.S143P patient. This experimental design controls for inter-individual genetic variation and demonstrates that the observed transcriptional and epigenomic changes are directly attributable to the S143P variant. In addition, we recruited and phenotyped an independent affected family member, the proband's mother, who also carries the *LMNA* p.S143P variant and has AF and conduction disease. iPSCs generated from her blood were differentiated into iPSC-aCMs. Consistent with the proband's findings, these cells exhibited reduced expression of *SCN5A* and *TBX5*. Electrophysiological analyses further demonstrated decreased action potential amplitude, reduced upstroke velocity, and diminished sodium current. Together, these data provide independent validation of the molecular and electrophysiological phenotypes observed in the proband-derived iPSC-aCMs (**Fig. 4c-d, Fig. 7**).

Genotyping confirmed that the proband's mother carries the AF-associated SNP rs6801957 within the *SCN10A* intronic enhancer (**Fig. S6**), supporting the relevance of the rare–common variant interaction in this family.

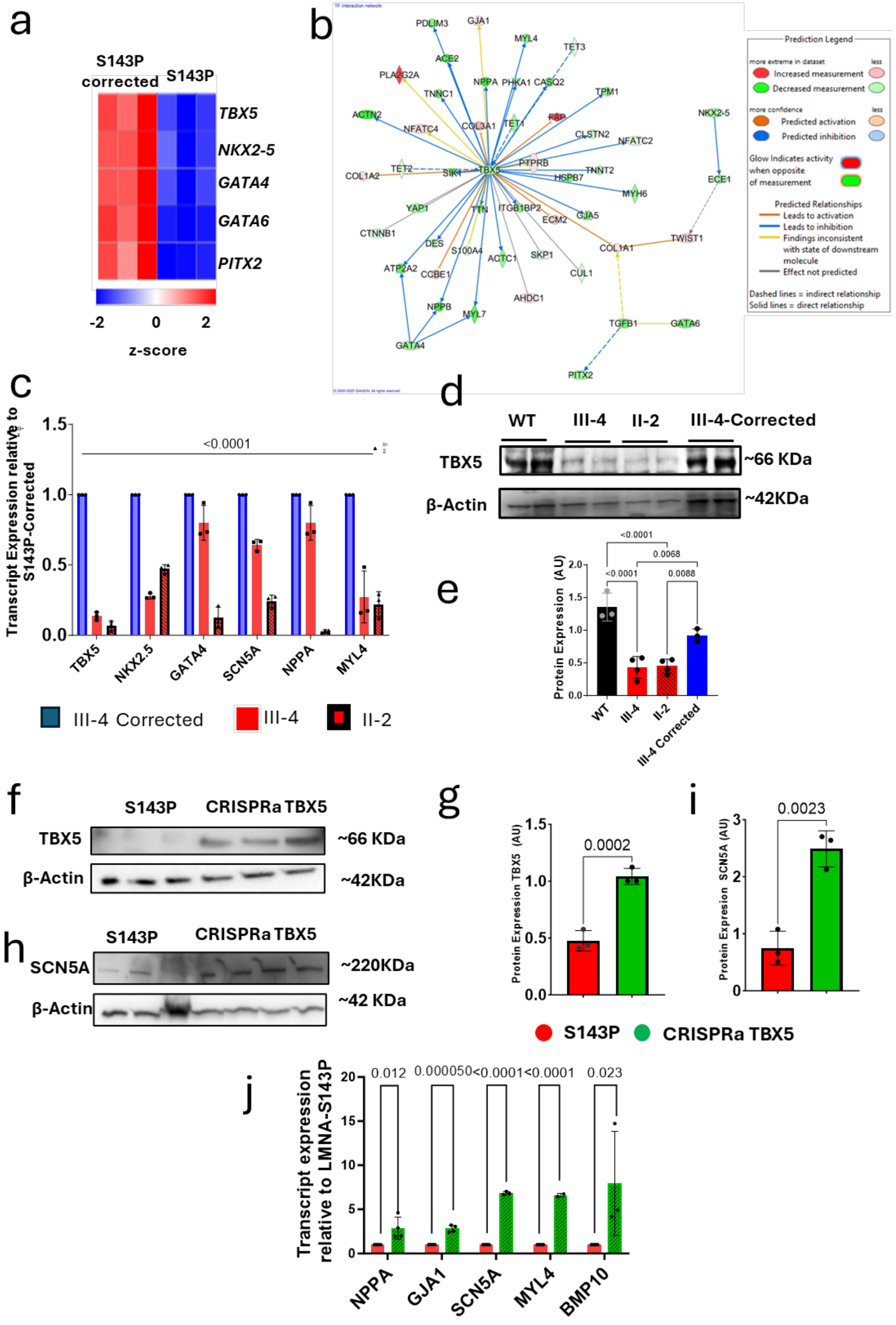

a
S143P corrected
S143P
TBX5
NKX2-5
GATA4
GATA6
PITX2
-2
0
2
z-score
b
PDLIM3
GJA1
MYL4
TET3
ACE2
PLA2G2A
NPPA
PHKA1
CASQ2
TNNC1
TPM1
ACTN2
NFATC4
COL3A1
TET1
FAP
NKX2-5
CLSTN2
NFATC2
COL1A2
TET2
SIK1
TBX5
PTPRB
TNNT2
HSPB7
MYH6
ECE1
YAP1
TTN
ITGB1BP2
ECM2
GJA5
CTNNB1
DES
S100A4
COL1A1
TWIST1
ACTC1
SKP1
ATP2A2
CCBE1
CUL1
AHDC1
NPPB
MYL7
TGFB1
GATA6
GATA4
PITX2
Prediction Legend
Increased measurement
Decreased measurement
Predicted activation
Predicted inhibition
Glow indicates activity when opposite of measurement
Predicted Relationships
Leads to activation
Leads to inhibition
Findings inconsistent with state of downstream molecule
Effect not predicted
Dashed lines = indirect relationship
Solid lines = direct relationship
c
Transcript Expression relative to S143P-Corrected
<0.0001
TBX5
NKX2.5
GATA4
SCN5A
NPPA
MYL4
III-4 Corrected
III-4
II-2
d
WT
III-4
II-2
III-4-Corrected
TBX5
~66 KDa
β-Actin
~42KDa
e
Protein Expression (AU)
<0.0001
0.0068
<0.0001
0.0088
WT
III-4
II-2
III-4 Corrected
f
S143P
CRISPRa TBX5
TBX5
~66 KDa
β-Actin
~42KDa
g
Protein Expression TBX5 (AU)
0.0002
h
S143P
CRISPRa TBX5
SCN5A
~220KDa
β-Actin
~42 KDa
i
Protein Expresion SCN5A (AU)
0.0023
S143P
CRISPRa TBX5
j
Transcript expression relative to LMNA-S143P
0.012
0.000050
<0.0001
<0.0001
0.023
NPPA
GJA1
SCN5A
MYL4
BMP10

Fig. 4: Disruption of atrial transcription factor (TF) network in *LMNA* p.S143P iPSC-aCMs. a. Expression of TFs regulating atrial cardiomyocyte lineage and implicated in AF TBX5 **b**. TF interaction network from IPA showing downregulation of transcription factors important for atrial contractility and rhythm control. **c**. Expression of TBX5 and other TFs in the network by qPCR. **d-e.** Protein expression of TBX5 quantified in d. **f-h.** Protein expression of TBX5 is increased after CRISPRa**. i.** CRISPRa rescues atrial-specific transcriptional targets of TBX5. **IPA** - Ingenuity Pathway Analysis.

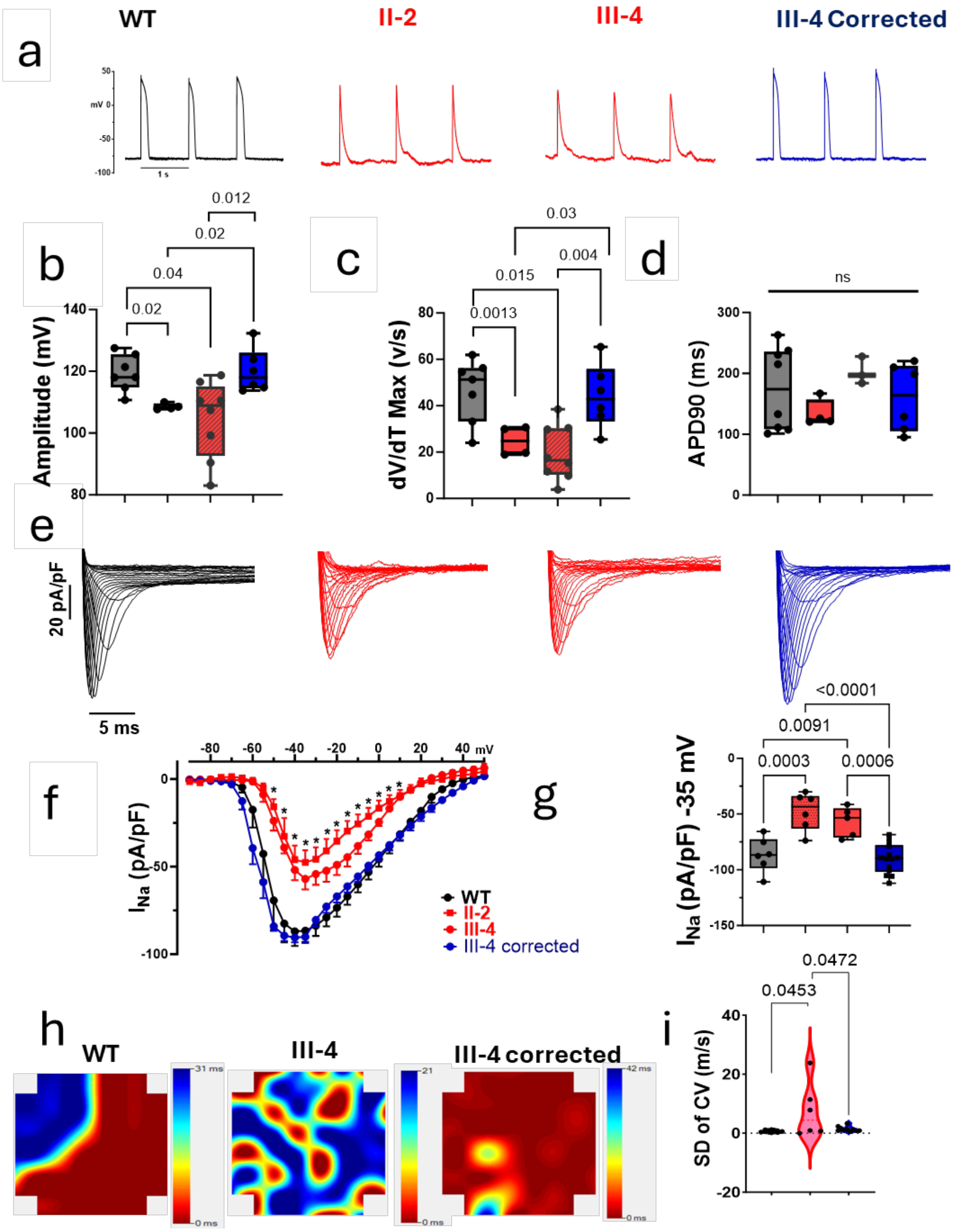

a
WT
II-2
III-4
III-4 Corrected
b
Amplitude (mV)
0.012
0.02
0.04
0.02
c
dV/dT Max (v/s)
0.03
0.004
0.015
0.0013
d
APD90 (ms)
ns
e
20 pA/pF
5 ms
f
I_{Na} (pA/pF)
mV
WT
II-2
III-4
III-4 corrected
g
I_{Na} (pA/pF) -35 mV
<0.0001
0.0091
0.0003
0.0006
h
WT
III-4
III-4 corrected
i
SD of CV (m/s)
0.0472
0.0453

Fig. 7: Reduced sodium current underlies electrophysiological alterations in *LMNA* p.S143P iPSC-aCMs. a. Representative evoked AP traces. **b-d.** Reduced AP amplitude and (c) upstroke velocity (dv/dt max), an indicator of atrial conduction in *LMNA* p.S143P iPSC-aCMs with (d) no change in APD90. **e.** Representative sodium current (I_{Na}) traces. **f-g.** Current-voltage (I/V) curve showing reduced I_{Na} in *LMNA* p.S143P iPSC-aCMs are quantified in a bar graph. *(a-g*, n = 5-7 cells per group, from three independent differentiations). (Ordinary one-way ANOVA with multiple comparisons *p<0.05*). **h-i**. Activation maps of propagation of electrical activity measured by an MEA show marked conduction heterogeneity in *LMNA* p.S143P iPSC-aCMs. (h-i. Increased standard deviation (SD) of conduction velocity measurements in *LMNA* p.S143P iPSC-aCMs. aCMs (n = 6 stimulated conduction velocity [CV] measurements for each group). (Ordinary one-way ANOVA with multiple comparisons, p<0.05).

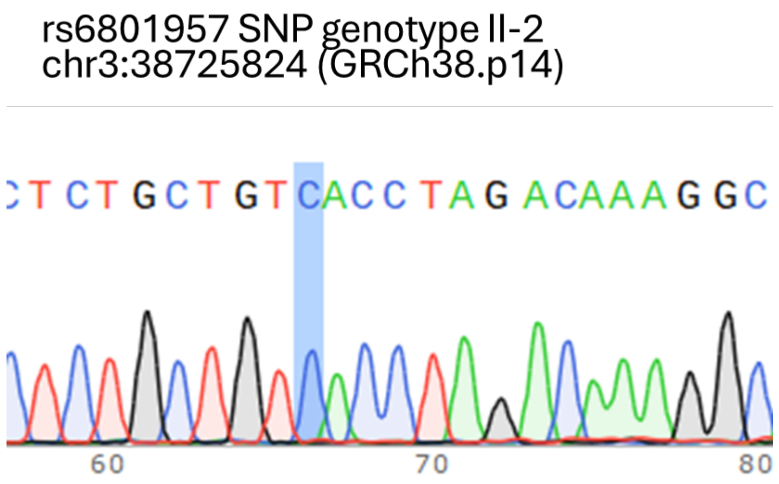

Fig. S6: Genotyping of AF-associated SNP rs6801957 in family member II-2.

6. Is any blinding occurring between patient and control cells for the scientist comparing assay results?

<u>Response</u>: Yes. All experimental assays and subsequent data analyses were conducted with the investigators blinded to the identity of patient versus control samples until after primary data processing was completed.

Reviewer #2:

This study links a pathogenic variant in LMNA to regulation of gene expression and epigenetic state in atrial-like cardiomyocytes derived from an iPSC line, then focuses on the reduced expression of the SCN5A gene and sodium current/conduction in iPSC-derived atrial-like cardiomyocyte model and shows that the reduced sodium current/conduction can be rescued by increasing SCN5A expression in the cell model. The authors claim that this is the first molecular evidence of epistatic gene-gene interactions driving arrhythmia risk and mechanistically links atrial myopathy and AF.

The claim of first molecular evidence is unfounded: Several published studies have shown such epistatic interactions in atrial arrhythmogenesis (e.g., PMID: 38189150 (Tbx5, Zfpm2); PMID: 34289528 (PDE4DIP; DES); PMID: 22402074; PMID: 31609246 (Tbx5, Pln); PMID: 27582060 (Tbx5, Pitx2); PMID: 36715501 (Tbx5, Prrx1)).

Furthermore, the epistatic interaction reported here is limited to one LMNA variant causing pleiotropic effects and one of many deregulated downstream effector genes (SCN5A) which in a dedicated cell model rescues one of the effects. In other words, from >8000 deregulated genes in the LMNA-S143P variant cell line, the authors select the downregulated gene that is known to be rate-limiting for sodium current and conduction in the myocardium, SCN5A (while largely ignoring all others). They then go on to show that sodium current and conduction are indeed reduced in this atrial cell model, and that when SCN5A is induced in the cell model, either by CRISPRa activation of the promoter or the previously established SCN5A enhancer, sodium current and conduction are increased. Taken together, this appears to be an analysis biased towards the outcome that was predicted.

Response: We thank the reviewer for their detailed and constructive feedback. We agree that prior studies have demonstrated epistatic interactions in atrial arrhythmogenesis, as illustrated by the cited examples. Considering this, we have revised the manuscript to remove the claim of “first molecular evidence” and instead emphasize that our work provides novel mechanistic insight into a specific rare–common variant interaction involving a *LMNA* mutation, altered chromatin accessibility at AF-associated loci, and downstream regulation of *SCN5A*.

While the concept of gene–gene interactions in AF is well-established, the novelty of our study lies in:

1. **The upstream variant context:** *LMNA* mutations are classically associated with structural cardiomyopathy. Here, we show that the *LMNA* p.S143P variant acts as a chromatin modifier, altering accessibility at multiple AF-associated regulatory loci, including the *SCN10A–SCN5A* enhancer.
2. **Direct epigenetic evidence:** We identify reduced accessibility at the *SCN10A* intronic enhancer (rs6801957) in *LMNA* p.S143P iPSC-aCMs, with the proband being homozygous for the risk allele. This is consistent with allele-specific regulatory disruption impacting *SCN5A* expression.
3. **Integration of genome-wide and functional data:** Our transcriptomic analysis revealed >8,000 deregulated genes in *LMNA* p.S143P vs. isogenic controls, with pathway analyses highlighting sodium channel biology among several affected processes. *SCN5A* was prioritized for follow-up because (i) it is a well-established effector of sodium current/conduction, (ii) it is a direct target of the enhancer showing altered accessibility, and (iii) its downregulation matches both the EP phenotype of the patient-derived model and established AF biology.

Our focus on *SCN5A* was therefore hypothesis-driven and supported by the data, rather than outcome-biased. To address the reviewer’s concern about novelty and breadth, we have expanded the Results and Supplementary Data to describe other dysregulated pathways and candidate genes, including those related to chromatin remodeling, metabolism, and structural cardiac integrity.

Additional functional validation to expand scope: We acknowledge that our initial functional assay targeted an enhancer already known to regulate *SCN5A*. To extend our mechanistic validation, we have functionally characterized two additional regulatory regions in *WDR1* and *ZFHX3* that harbor AF-associated SNPs and show differentially increased accessibility in *LMNA*-p.S143P iPSC-aCMs (**Fig. 6**). Please see the overall response to Reviewer 1.

***TBX5* activation as an independent proof-of-concept for rescuing TF networks:** We have also performed CRISPR activation of *TBX5*, which led to robust upregulation of *TBX5* itself and several of its downstream target genes (**Fig. 4**). These results provide an example of disruption of TF networks in *LMNA* p.S143P iPSC-aCMs that can be functionally manipulated to rescue the expression of genes within the network.

Collectively, these additions: **(i)** functional characterization of novel AF-associated enhancers using CRISPRa/i, and **(ii)** demonstration of *TBX5* network activation, address the reviewer's concerns by expanding the functional scope of our study, diversifying the regulatory loci examined, and reinforcing the broader mechanistic link between *LMNA*-mediated chromatin remodeling and AF pathogenesis.

Specific Comments

1) In their differential gene expression analysis and chromatin accessibility profiling, the authors compare atrial CMs derived from only 1 line with the LMNA variant and 1 control line, while it is known that line-to-line and differentiation to differentiation differences account for a large fraction of variation. Moreover, they compared an LMNA-S143P iPSC line with a WT line (the genetic relationship between the two lines is not clear from the manuscript). Different genetic backgrounds (assuming they are of different genetic background) further contribute to transcriptome and accessibility differences which cannot be discriminated from the effects of the LMNA variant. Given the central role of these data sets in this study and its claims, the transcriptome/accessibility data should be much more solid, and obtained from multiple independent cell lines, isogenic controls (the authors actually made such a line but did not use it), and differentiation batches to uncover the LMNA-variant-dependent effects on transcription and accessibility.

Response: We thank the reviewer for this important comment regarding the need for isogenic controls and replication across differentiation batches. In the revised manuscript, we have now addressed both points:

1. **Use of isogenic controls:** We have performed both ATAC-seq and RNA-seq using CRISPR-corrected isogenic iPSC-aCMs from the *LMNA* p.S143P proband. This design eliminates inter-individual genetic background differences and ensures that the observed transcriptomic and chromatin accessibility changes are attributable specifically to the *LMNA* p.S143P variant. These isogenic data now serve as the primary basis for our mechanistic analyses (**Fig. 1**).

2. **Replication across differentiation batches:** All isogenic and non-isogenic comparisons have been repeated using multiple independent differentiation batches to account for batch-to-batch variability inherent in iPSC-derived cardiomyocyte studies. The consistent detection of key differential expression and accessibility patterns across batches strengthens the robustness of our findings.

We have updated the Methods to clarify the genetic relationship between the lines, the number of differentiation replicates performed, and the statistical approaches used to account for variability. Together, these additional experiments and clarifications directly address the reviewer's concerns and substantially strengthen the reliability of our transcriptomic and epigenomic conclusions.

2) Data analysis: Figure 2g shows correlation between ATAC seq and RNAseq data, but the authors only show positively correlating data, leaving out the negatively correlating data. Then, they show correlation of the data sets, including a statistical test, which, of course, is positive and significant because the negatively correlating data has been left out. Figure 3a same.

Response: To determine whether the *LMNA* variant disrupts regulatory interactions, we assessed whether alterations in chromatin accessibility at promoters and enhancers correlate with gene expression.

- **Promoters**: The correlation between promoter accessibility and gene expression remains positive and significant even after including the non-correlating data (**Fig. 3**, above), which suggests that a substantial proportion of changes in gene expression can be attributed to chromatin accessibility changes due to the *LMNA* variant**.**
- **Enhancers:** Compared to promoters, the assignment of target genes of enhancers is not as straightforward. To filter likely target genes of putative enhancers, we selected genes for analysis that showed concordant changes in gene expression and chromatin accessibility. However, we acknowledge that assigning enhancers based on H3K27ac and PCHiC interactions is not confirmatory and would yield many false positives. We have not included that data in the revised manuscript.

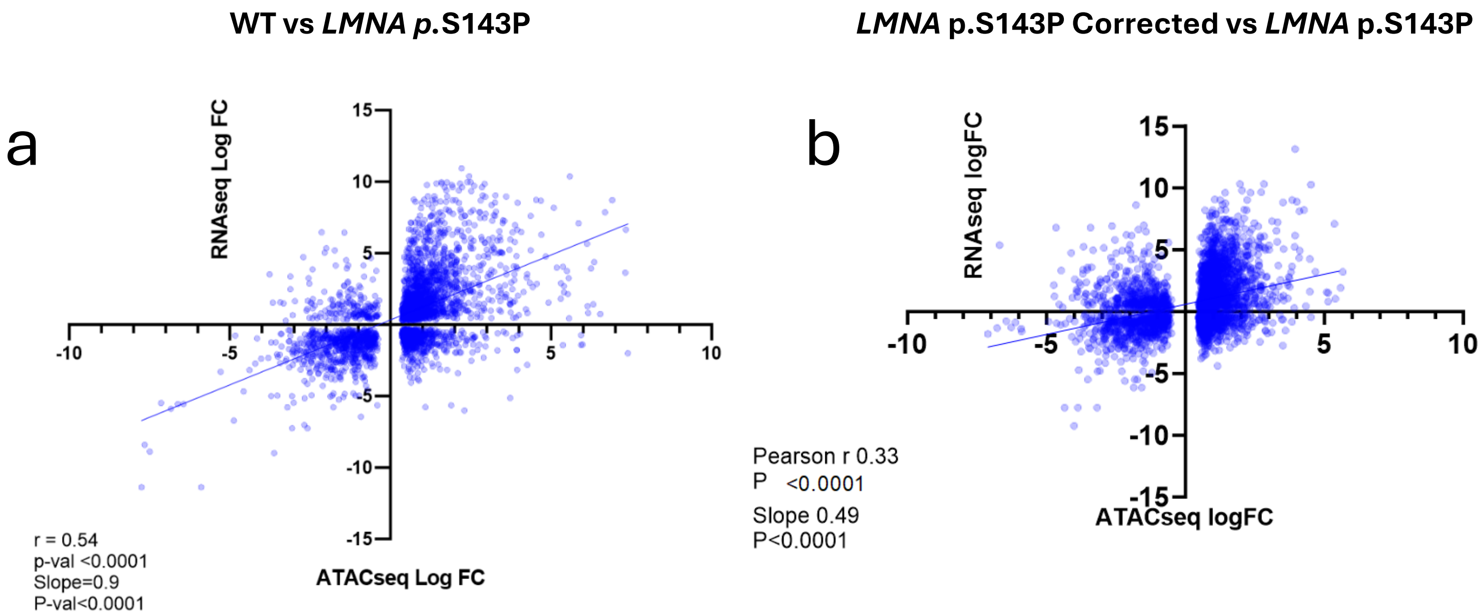

Correlation between promoter, accessibility, and gene expression. a. Genome-wide correlation between promoter chromatin accessibility (ATAC-seq) and corresponding gene expression levels (RNA-seq) in WT versus *LMNA* p.S143P iPSC-aCMs. **b.** Correlation between promoter accessibility and gene expression in CRISPR-corrected isogenic versus *LMNA* p.S143P iPSC-aCMs. In both comparisons, all promoter–gene pairs are included, and positive, statistically significant correlations indicate that changes in promoter accessibility are associated with corresponding changes in transcription.

3) What is the difference between expression data shown in figure 3c and 3h?

Response: In the original manuscript, **Fig. 3c** displayed fold changes in gene expression alongside changes in chromatin accessibility at putative enhancers, inferred from H3K27ac enrichment and PCHiC interactions. As noted above, because enhancer–gene assignment is not definitive and could introduce false positives, this analysis has been removed from the revised manuscript. **Fig. 3h**, in contrast, presents fold changes in expression for *TBX5* transcriptional targets, identified

from published datasets, to illustrate the downstream effects of *TBX5* modulation in our iPSC-aCM model.

4) ***Differences in accessibility in Figure 3d-g and 4a-d are challenging to see and do not seem to support the claimed changes in all cases. More importantly, they should be quantified.***

Response: We thank the reviewer for this observation. The differences in chromatin accessibility were quantified in the original manuscript (Figs. 3c, 4e) and in Table S1. In the revised manuscript, we have enhanced figure clarity by improving the visualization of accessibility tracks and now present the quantitative data directly alongside the corresponding genome browser views. This side-by-side presentation should make the magnitude and direction of accessibility changes more apparent to the reader (**Fig. 5**).

Furthermore, the authors assume the accessible sites they point out are enhancers of the target genes they focus on. The assumption is made that H3K27ac signal and accessibility demarcate enhancers and that interactions between H3K27ac-enriched genomic sequences and promoters captured by published PC-HiC data shows that these enhancers regulate such promoters. However: 1) degree of accessibility does not correlate well with enhancer or promoter activity. 2) Neither H3K27ac nor accessibility specifically demarcate enhancers (only a fraction of the accessible sites functions as enhancer). 3) A large fraction of the interactions seen in (PC) HiC is structural, not regulatory. Therefore, observed interactions should not be interpreted as functional enhancer-promoter interactions. 4) the PC HiC data is not saturating, many interactions are missed.

The authors do not use data or even refer to studies in which efforts have been made to try and identify AF-relevant regulatory sequences including enhancers, their target genes and the identification of functional SNPs among the many SNPs in LD with the lead SNP in the association (PMID: 35064145; PMID: 37591828; PMID: 31628324; PMID: 32248749; PMID: 33155827). Their data and analysis should be compared to those data sources and could be improved by integrating them.

Response: We acknowledge the limitations of relying solely on H3K27ac enrichment and PCHiC interactions to assign enhancer–gene relationships. We thank the reviewer for the helpful suggestions and have revised our analysis to incorporate additional lines of evidence to prioritize regulatory regions and associated SNP–gene pairs more rigorously. Specifically, we now integrate:

1. Cardiac eQTLs from human heart tissue.
2. Published datasets from studies that have prioritized AF-relevant enhancers and SNP-gene pairs using bioinformatic or functional assays (including the studies cited by the reviewer, PMID: 35064145; PMID: 37591828; PMID: 31628324; PMID: 32248749; PMID: 33155827).
3. Additional SNPs in LD within the differentially accessible peaks using the GWAS catalog (**Fig. S4).**
4. Risk allele status in the proband.
5. Statistical fine mapping and functional annotations from previous studies (e.g., STARR-seq, single-cell ATAC-seq, orthologous mouse deletion data).
6. We have improved regulatory annotations by aligning ChromHMM annotations (**Fig. 5**).

These combined data are summarized in **Table S11**, which includes, for each locus, the index SNP, evidence of SNPs in LD, mapped gene(s), eQTL status, ATAC-seq fold change, statistical significance, gene expression change, H3K27ac status, PCHiC interactions, and supporting evidence from prior studies.

Based on this integrated approach, we have functionally tested six AF variant regions using CRISPR epigenetic modifiers, followed by measuring expression of predicted target genes. Our approach was validated on additional variant-gene pairs, in *WDR1* and *ZFHX3*, in addition to the previously validated variant region in *SCN10A*.

We believe these revisions directly address the reviewer's concerns by:

- Reducing reliance on accessibility and H3K27ac alone for enhancer identification
- Incorporating orthogonal datasets, including functional genomics and eQTL evidence
- Expanding functional validation to a novel AF-associated enhancer beyond the known SCN10A locus

Table S11: Approach for prioritization of enhancers and target gene

Index SNP	Evidence of a functional SNP in LD	Mapped Gene based on proximity	eQTL*		ATAC Fold Change	P-Value	Differential Expression in LMNA-S143P iPSC-aCMS	H3K27ac	PC-Hi C	Chromatin State	Supporting evidence of SNP -gene pair from previous studies	Ref
			HAA	LV								
rs4896104		ALDH8A1, SGK1			-2.30	0.001		+	+	Quiescent		
rs7172038		HCN4, NPTN			-3.109	9.89E-07	+	+	+	Enhancer	Gene PIP 0.7 SNP PIP 0.9	5
							+				STARR-seq and confirmation with deletion of the orthologous region in mouse	6
											Custom prioritization score (TAD, PCHiC, heart tissue expression adult and human, eQTL)	7
rs6801957		SCN10A		+	-1.34	0.003		+		Enhancer	SCN10A PIP 0.5<br>SCN5A PIP 0.4,<br>SNP PIP 0.8	5
											STARR-seq	6
rs1769758		ZMIZ1	+		-1.68	1.80E-06		+		Enhancer	SC-ATAC seq to identify OCR, Statistical Fine Mapping	5
rs2540949	rs1009358	CEP68	+	+	0.93	0.0008	+	+		TSSfl	Gene PIP 0.2, SNP PIP 0.2	5
rs12640611		SLC2A9	+		1.88	2.03E-05		+		Enhancer		
rs1886512		KLF12	+	+	2.54	0.0003		+		Enhancer		
rs2359171	rs2106261	ZFHX3			-1.60	0.0003	+	+		Enhancer	Gene PIP 0.8<br>rs2106261 PIP 1.0	5

* eQTLv10, HAA- heart atrial appendage, LV- left ventricle, OCR-open chromatin region

a

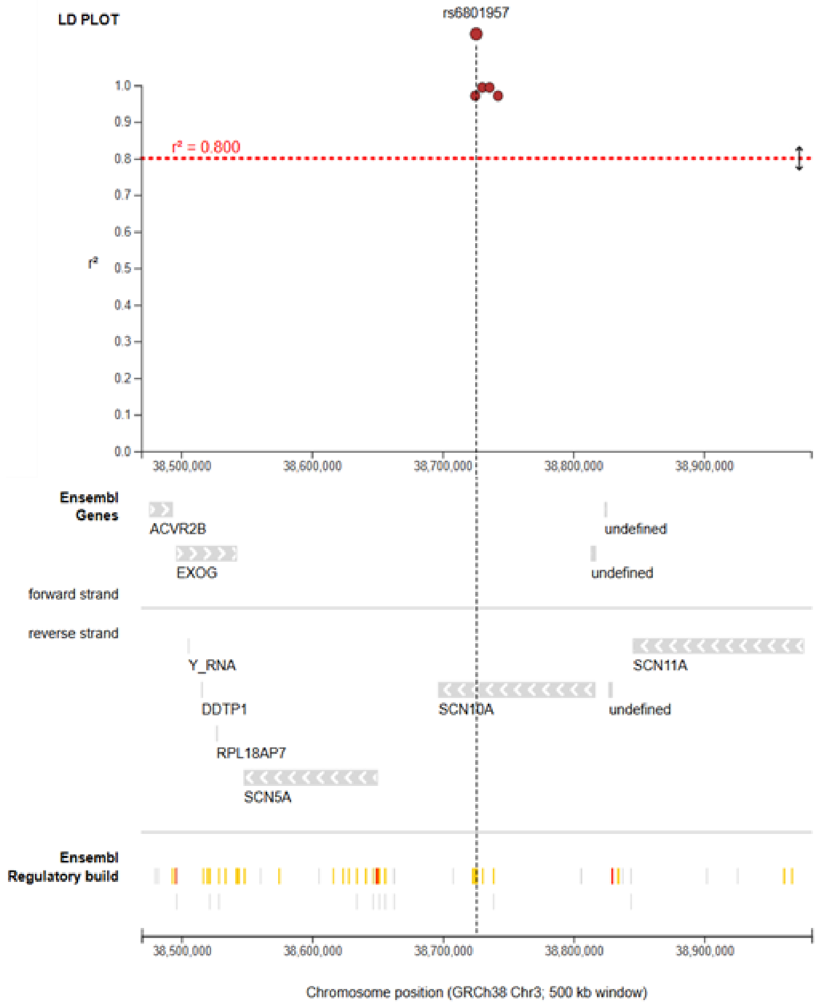

LD PLOT
rs6801957
r² = 0.800
1.0
0.9
0.8
0.7
0.6
r²
0.5
0.4
0.3
0.2
0.1
0.0
38,500,000
38,600,000
38,700,000
38,800,000
38,900,000
Ensembl Genes
ACVR2B
EXOG
undefined
undefined
forward strand
reverse strand
Y_RNA
DDTP1
RPL18AP7
SCN5A
SCN10A
SCN11A
undefined
Ensembl Regulatory build
38,500,000
38,600,000
38,700,000
38,800,000
38,900,000
Chromosome position (GRCh38 Chr3; 500 kb window)

b

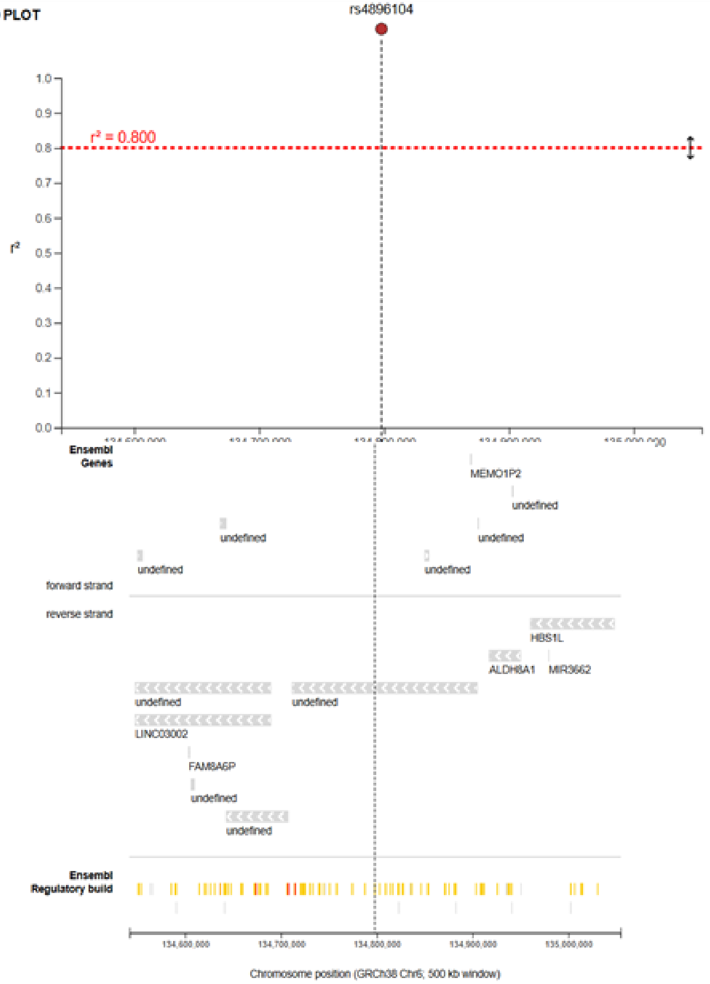

LD PLOT
rs4896104
r² = 0.800
1.0
0.9
0.8
0.7
0.6
r²
0.5
0.4
0.3
0.2
0.1
0.0
Ensembl Genes
MEMO1P2
undefined
undefined
undefined
undefined
undefined
forward strand
reverse strand
HBS1L
ALDH8A1
MIR3662
undefined
undefined
LINC03002
FAM8A6P
undefined
undefined
Ensembl Regulatory build
134,600,000
134,700,000
134,800,000
134,900,000
135,000,000
Chromosome position (GRCh38 Chr6; 500 kb window)

c

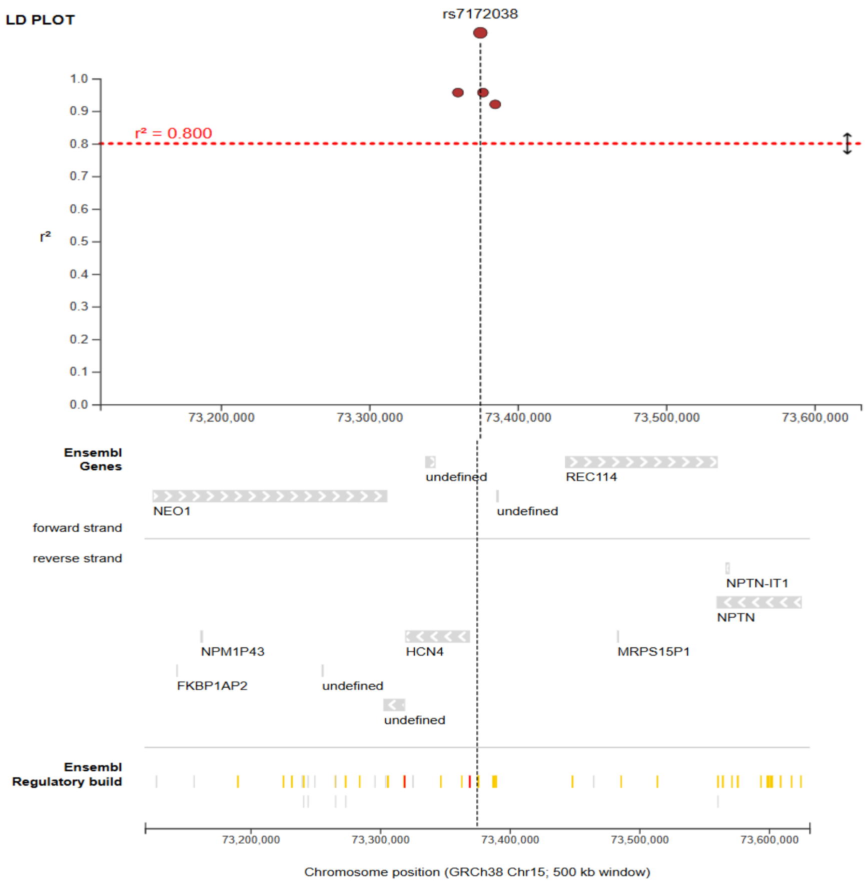
LD PLOT
rs7172038
1.0
0.9
0.8
0.7
0.6
0.5
0.4
0.3
0.2
0.1
0.0
r²
r² = 0.800
73,200,000
73,300,000
73,400,000
73,500,000
73,600,000
Ensembl Genes
undefined
REC114
NEO1
undefined
forward strand
reverse strand
NPTN-IT1
NPTN
NPM1P43
HCN4
MRPS15P1
FKBP1AP2
undefined
undefined
Ensembl Regulatory build
73,200,000
73,300,000
73,400,000
73,500,000
73,600,000
Chromosome position (GRCh38 Chr15; 500 kb window)

d

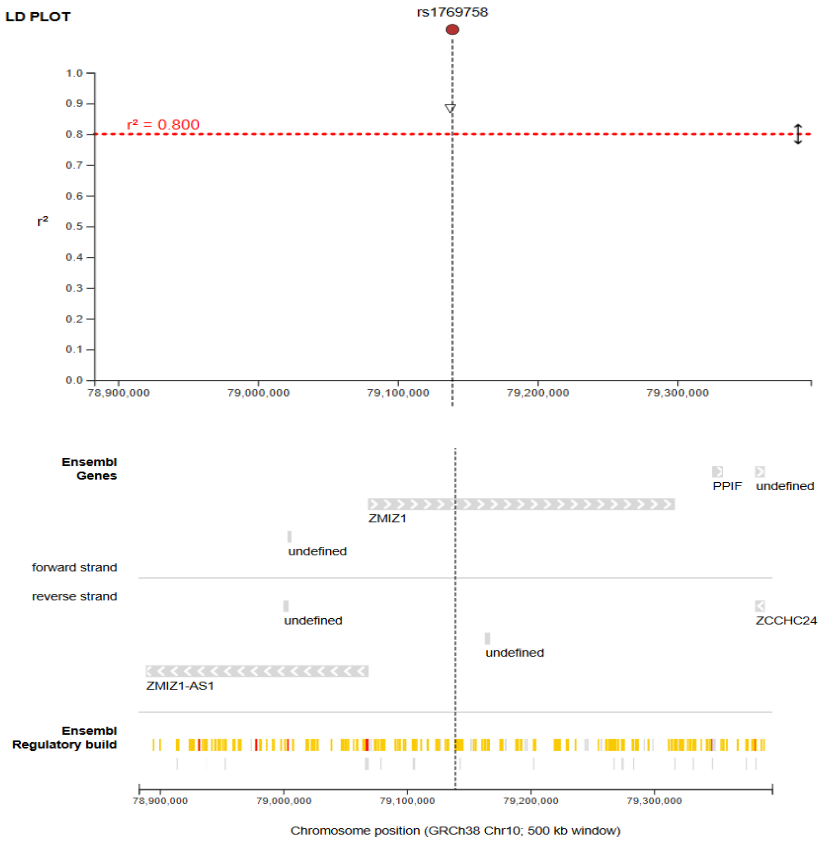
LD PLOT
rs1769758
1.0
0.9
0.8
0.7
0.6
0.5
0.4
0.3
0.2
0.1
0.0
r²
r² = 0.800
78,900,000
79,000,000
79,100,000
79,200,000
79,300,000
Ensembl Genes
PPIF
undefined
ZMIZ1
undefined
forward strand
reverse strand
undefined
ZCCHC24
undefined
ZMIZ1-AS1
Ensembl Regulatory build
78,900,000
79,000,000
79,100,000
79,200,000
79,300,000
Chromosome position (GRCh38 Chr10; 500 kb window)

e

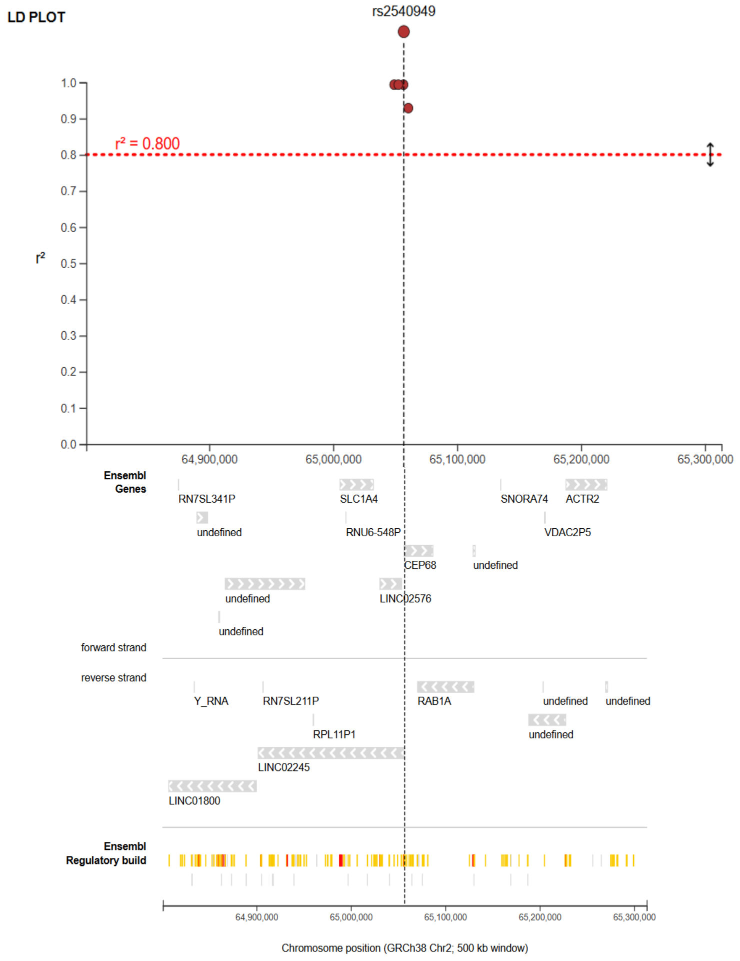

LD PLOT
rs2540949
r² = 0.800
r²
1.0
0.9
0.8
0.7
0.6
0.5
0.4
0.3
0.2
0.1
0.0
64,900,000
65,000,000
65,100,000
65,200,000
65,300,000
Ensembl Genes
RN7SL341P
SLC1A4
SNORA74
ACTR2
undefined
RNU6-548P
VDAC2P5
CEP68
undefined
undefined
LINC02576
undefined
forward strand
reverse strand
Y_RNA
RN7SL211P
RAB1A
undefined
undefined
RPL11P1
undefined
LINC02245
LINC01800
Ensembl Regulatory build
64,900,000
65,000,000
65,100,000
65,200,000
65,300,000
Chromosome position (GRCh38 Chr2; 500 kb window)

f

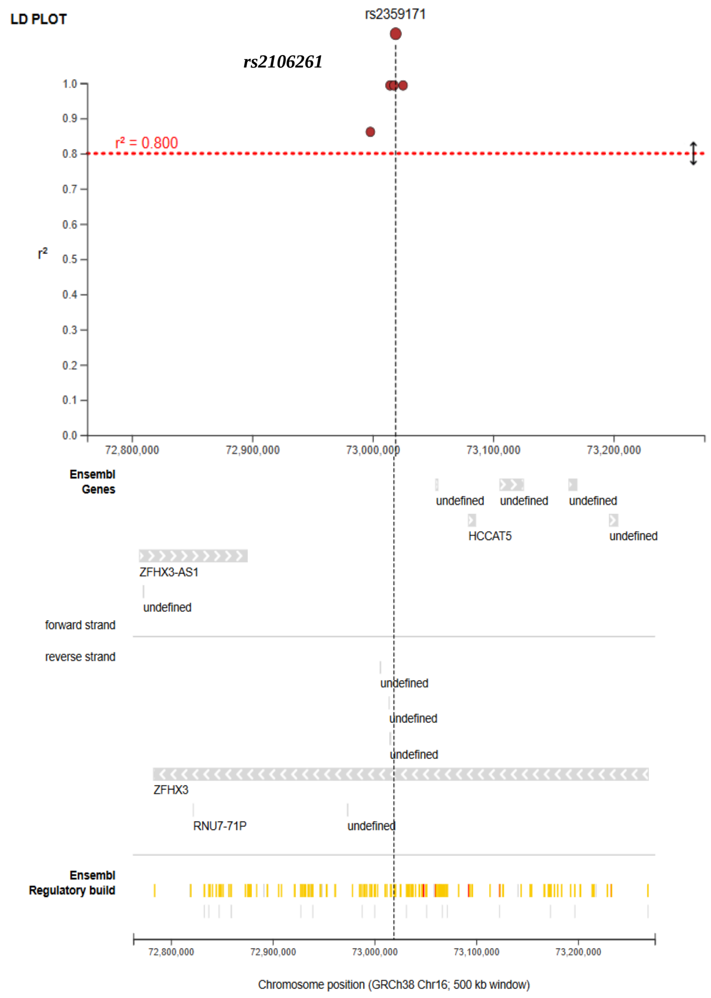

LD PLOT
rs2359171
rs2106261
r² = 0.800
r²
1.0
0.9
0.8
0.7
0.6
0.5
0.4
0.3
0.2
0.1
0.0
72,800,000
72,900,000
73,000,000
73,100,000
73,200,000
Ensembl Genes
undefined
undefined
undefined
HCCAT5
undefined
ZFHX3-AS1
undefined
forward strand
reverse strand
undefined
undefined
undefined
ZFHX3
RNU7-71P
undefined
Ensembl Regulatory build
72,800,000
72,900,000
73,000,000
73,100,000
73,200,000
Chromosome position (GRCh38 Chr16; 500 kb window)

g

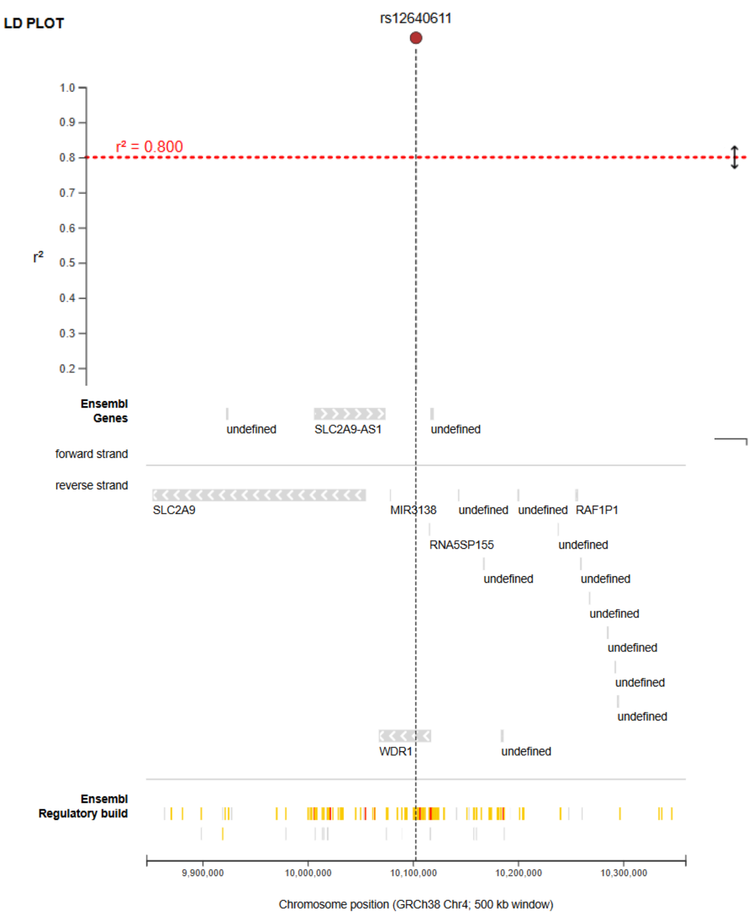
LD PLOT
rs12640611
r² = 0.800
r²
1.0
0.9
0.8
0.7
0.6
0.5
0.4
0.3
0.2
Ensembl Genes
forward strand
reverse strand
undefined
SLC2A9-AS1
SLC2A9
MIR3138
RAF1P1
RNA5SP155
WDR1
Ensembl Regulatory build
9,900,000
10,000,000
10,100,000
10,200,000
10,300,000
Chromosome position (GRCh38 Chr4; 500 kb window)

h

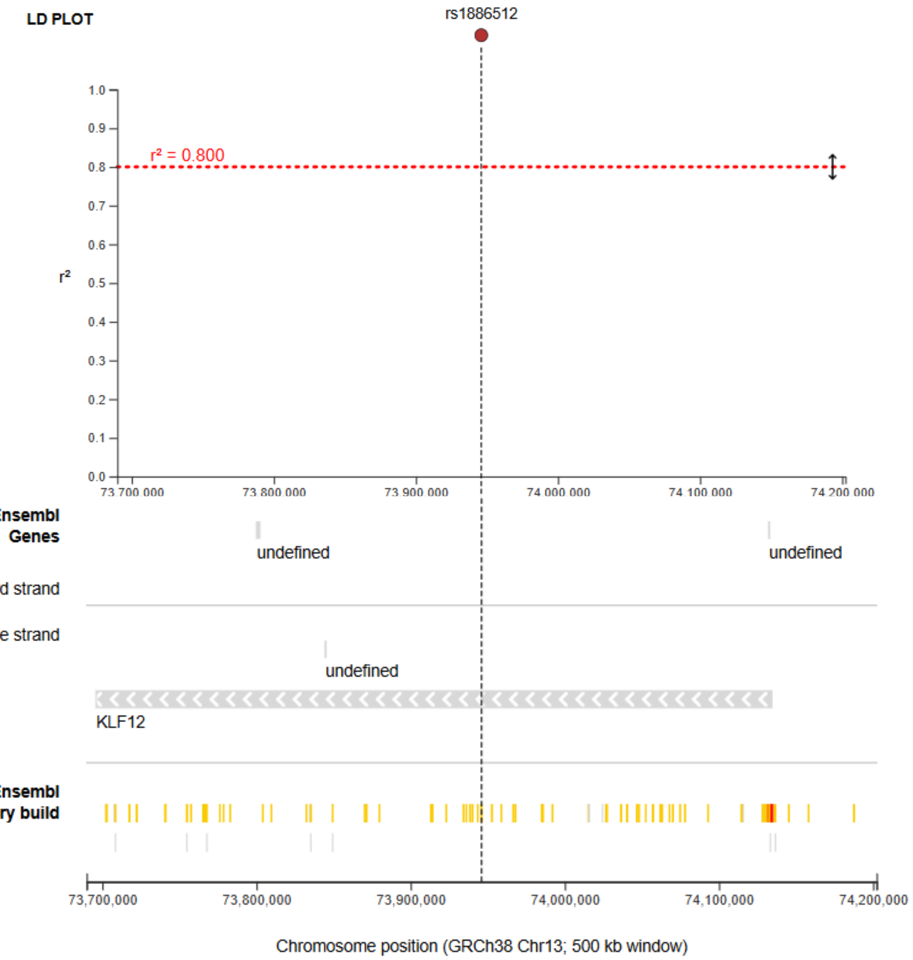
LD PLOT
rs1886512
r² = 0.800
r²
1.0
0.9
0.8
0.7
0.6
0.5
0.4
0.3
0.2
0.1
0.0
73 700 000
73 800 000
73 900 000
74 000 000
74 100 000
74 200 000
Ensembl Genes
forward strand
reverse strand
undefined
KLF12
Ensembl Regulatory build
73,700,000
73,800,000
73,900,000
74,000,000
74,100,000
74,200,000
Chromosome position (GRCh38 Chr13; 500 kb window)

Fig. S4: LD Plots for AF SNPs in regions of differential accessibility. [Editorial note: from the GWAS catalog. GWAS: Catalog of Published Genome-Wide Association Studies (RRID:SCR_012745) under the license https://creativecommons.org/licenses/by/4.0/]

Filtering criteria for SNPs in LD

- r^2=0.8
- Associated with a cardiovascular trait
- Population: British in England and Scotland
- Within 500Kb

5) beta actin in Fig. 1d and Fig. 4f seem identical, was this taken from the same blot?

<u>Response</u>: We have included separate blots for *SCN5A* and Lamin A/C (**Fig. 2d, Fig. S7**).

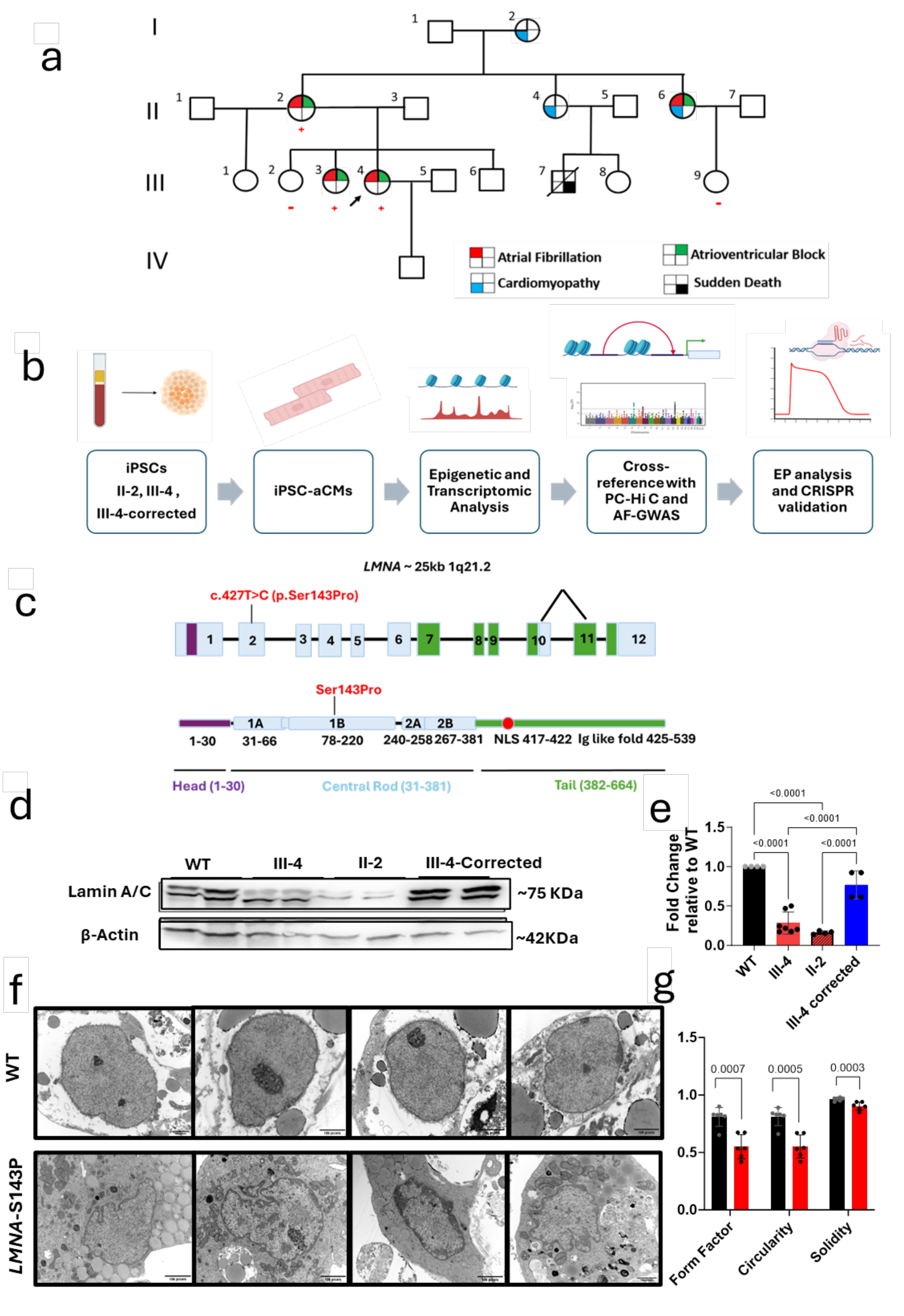

[Editorial note: figure 2b Created in BioRender. Owais, A. (2026) https://BioRender.com/rqgp5qh]

Fig. 2: The *LMNA* p.S143P variant reduces expression of Lamin A/C and alters nuclear morphology. a. Schematic representation of the family pedigree, highlighting affected members with a history of atrial fibrillation (AF), atrioventricular (AV) block, cardiomyopathy, and sudden death. The arrow indicates the proband. Circles denote females; squares denote males. The plus (+) and minus (-) signs indicate the presence and absence of the *LMNA* p.S143P variant, respectively. **b.** Schematic illustrating the nucleotide and amino acid change (NM_170707.4(LMNA):c.427T>C [p.Ser143Pro]) in exon 2 and the central rod domain. The arrowhead marks an alternative splice site location for generating Lamin A and C transcripts. **c.** Overview of the study design. **d-e.** Protein expression analysis of Lamin A/C in wild-type (WT), LMNA p.S143P, and S143P-corrected iPSC-aCMs. **f-g**. Nuclear deformities observed in *LMNA* p.S143P iPSC-aCMs by electron microscopy.

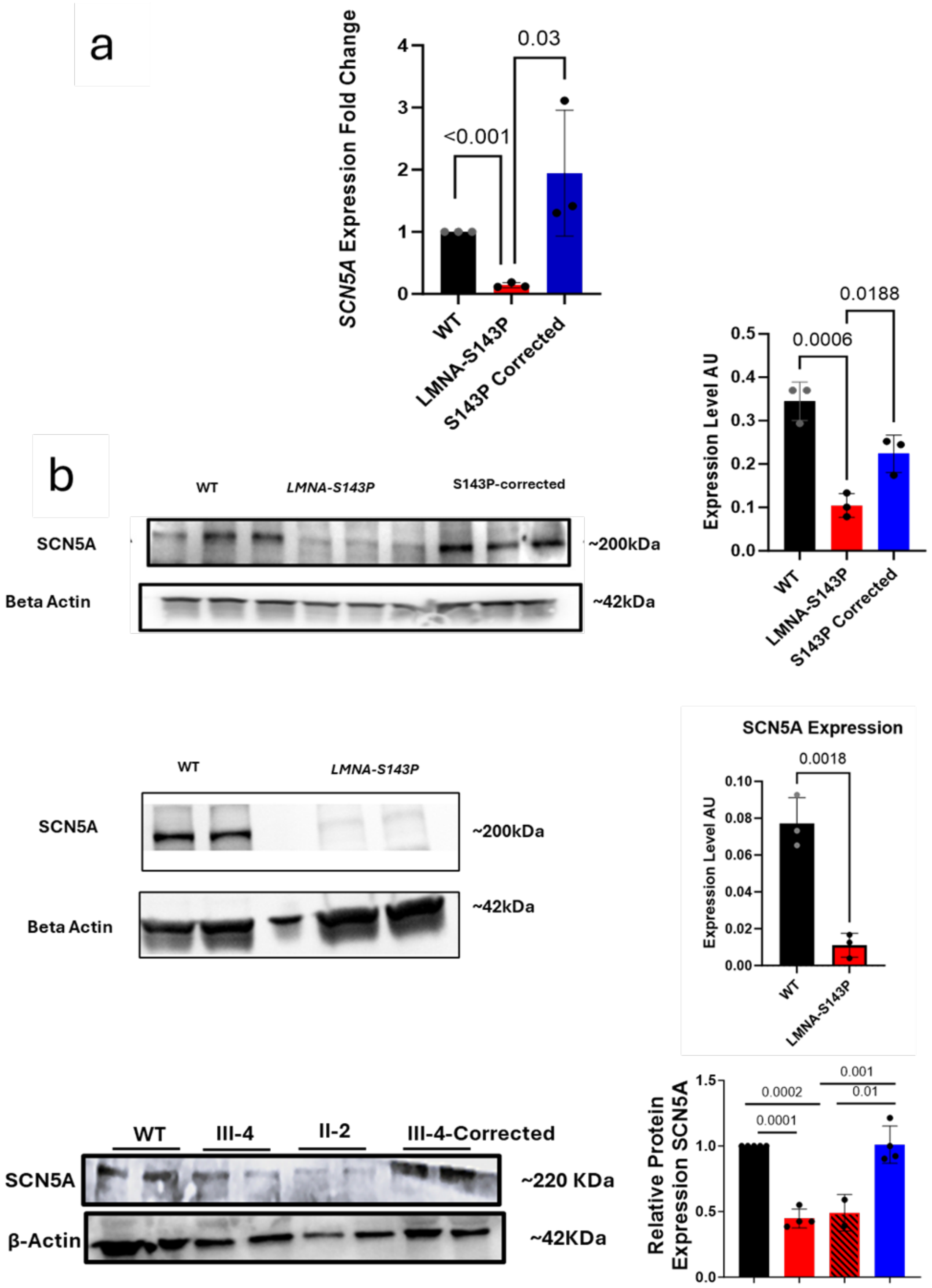

Fig. S7: a. Reduced mRNA expression of *SCN5A* in *LMNA* p.S143P iPSC-aCMs by qPCR. **b.** Reduced protein expression of *SCN5A* in *LMNA* p.S143P iPSC-aCMs in samples from multiple differentiations.

6) the authors show that in comparison to the wt line, TBX5 and other TFs essential for driving the (atrial) CM lineage gene programs are downregulated. Whether this is due to the LMNA variant remains uncertain (see also comment 1), and it would be important to establish this. But assuming it is, this would then to a large extent explain downregulation of SCN5A (and other CM genes), providing an explanation for the "epistatic interaction" between LMNA and SCN5A. Because TBX5 is an AF associated gene itself that has been well studied in the context of atrial CM lineage specification and maintenance, dosage dependent mechanisms of action, AF and SCN5A regulation, this then sheds a different light on the conclusions. If LMNA indeed pleiotropically disrupts the transcription factor network regulating atrial CM gene expression, then any effector gene regulating by the key transcription factors of this network will be deregulated. This would also not be specific to AF, but to the overall state of the atrial CMs. This then would be compatible with authors findings, that 8000 genes are deregulated and 45 k sites show differential accessibility.

<u>Response</u>: We thank the reviewer for this thoughtful perspective. Our revised RNA-seq analysis using CRISPR-corrected isogenic iPSC-aCMs confirms that the *LMNA* p.S143P variant is associated with downregulation of multiple TFs critical for atrial cardiomyocyte lineage specification and maintenance, including *TBX5*, *GATA4*, and *GATA6* (**Fig. 4**). These results indicate that the TF changes are indeed attributable to the *LMNA* variant, rather than to differences in genetic background.

We further confirmed that several atrial-specific genes co-regulated by *TBX5* and other atrial TFs are also downregulated in *LMNA* p.S143P iPSC-aCMs. Given *TBX5*'s central role as a regulator of an atrial enhancer network and its known involvement in *SCN5A* regulation, we hypothesized that restoring *TBX5* levels could rescue downstream transcriptional programs. To test this, we performed CRISPR-mediated activation of *TBX5*, which resulted in robust *TBX5* upregulation and rescue of multiple known *TBX5* target genes, including *SCN5A* (**Fig. 4**).

These results suggest that the *LMNA* p.S143P variant may pleiotropically disrupt the atrial TF network, leading to broad transcriptional reprogramming that includes but is not limited to genes directly relevant to AF pathogenesis. While this mechanism is consistent with the large number of deregulated genes and accessibility changes observed, our functional experiments also demonstrate that chromatin remodeling at specific AF-associated loci (e.g., *SCN10A–SCN5A* enhancer, *ZFHX3* enhancer) can modulate the expression of individual disease-relevant effectors, highlighting a potential convergence between global transcriptional network disruption and targeted regulatory alterations.

7) The authors took all AF risk loci from recent GWAS studies (how many?) and find that 8 loci overlap with their altered accessibility regions. Please specify "loci" and "regions". It is not clear whether this is a significant finding, i.e. whether this is statistically enriched overlap. Of note, most AF risk loci are found in CM lineage relevant gene regions, and the differential accessibility data set the authors used to overlap with AF GWAS variants is derived from CMs. Moreover, it is not clear whether the authors focused on the lead SNP, which may not be functional, or all SNPs in LD with the lead SNP (a few of which may be functional). This also affects the assignment of relevant variants, assignment of enhancers (see above), their colocalization, the assignment of enhancers to target genes (see also above). Overall, many

assumptions are made regarding variant enhancers of target genes, which however should be demonstrated using functional assays or supported by published functional data.

<u>Response</u>: We appreciate the reviewer's comments and have clarified our analysis in the revised manuscript as follows:

<u>First</u>, the overlap analysis refers specifically to statistically significant differential ATAC-seq peaks (adjusted $P < 0.05$) that harbor AF-associated variant regions. In addition to peak-specific differences, we observed a generalized reduction in accessibility across the *SCN5A–SCN10A* locus, suggesting a broader effect on chromatin architecture in this region. The quantification of differential accessibility is presented in **Fig. 5** and was previously shown in Figs. 3c, 4e, and Table S1.

<u>Second</u>, in the isogenic control vs. *LMNA* p.S143P comparison, our overlap analysis replicated 7 of the 8 previously identified AF-associated regions from the non-isogenic comparison and identified an additional variant region near *ZFHX3* (**Table S7-8**). This reproducibility supports the likelihood that these regions play an important role in modulating AF-relevant gene expression.

<u>Third</u>, our primary analysis was based on lead SNPs from recent AF GWAS studies. Still, we also evaluated all SNPs in strong LD that fell within differentially accessible peaks (**Fig. S6**). For each locus, we integrated:

- Cardiac eQTL data
- Evidence from prior publications that prioritized SNP–gene pairs or provided functional validation, and
- The proband's genotype at the risk allele

This integrated approach, summarized in **Table S9**, reduces reliance on accessibility alone for enhancer–gene assignment.

<u>Finally</u>, beyond the intronic *SCN10A* SNP that we have already functionally validated, we have now validated additional AF-associated regulatory regions in *WDR1* and *ZFHX3*, further supporting the functional impact of these variant regions on target gene expression.

8) The rs6801957 the authors focus on was indeed be found to disrupt an enhancer of SCN5A but later was reported to only mildly affect SCN5A expression when disrupted, but cause the downregulation of a cardiac-specific short isoform of SCN10A driven from an intronic promoter close by (PMID: 33910361). This caused reduced sodium current. These observations have an impact on the explanations and conclusions of the current manuscript and should be taken into account.

<u>Response</u>: We thank the reviewer for highlighting this important study. To address this point, we quantified *SCN10A* expression in WT, *LMNA* p.S143P iPSC-aCMs, and CRISPR-corrected isogenic iPSC-aCMs. In all three groups, *SCN10A* expression was almost undetectable (**Fig. S10)**, suggesting that the full-length *SCN10A* does not play a major role in the phenotype observed in our model.

We acknowledge, however, that the short cardiac-specific isoform of *SCN10A* described in PMID: 33910361 may not be detected in standard RNA-seq analyses. Therefore, we measured the expression of the shorter *SCN10A* isoform using primers targeting the last seven exons. We found that while the expression of *SCN10A* short was reduced in the *LMNA* p.S143P iPSC-aCMs, CRISPRa of the *SCN10A* enhancer was able to rescue the expression of both *SCN5A* and the

SCN10A short (**Fig. S10**), suggesting that the intronic enhancer regulates the expression of both *SCN5A* as well as *SCN10A* short (**Fig. 6**).

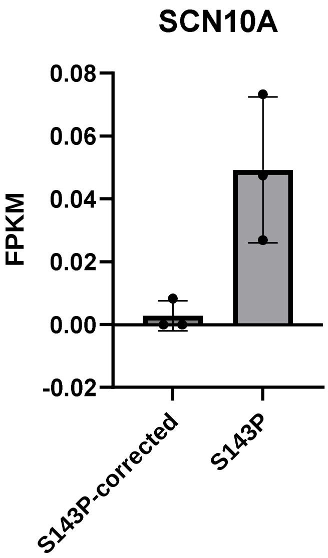

Fig. S10: Minimal expression of *SCN10A* full-length from RNA sequencing.

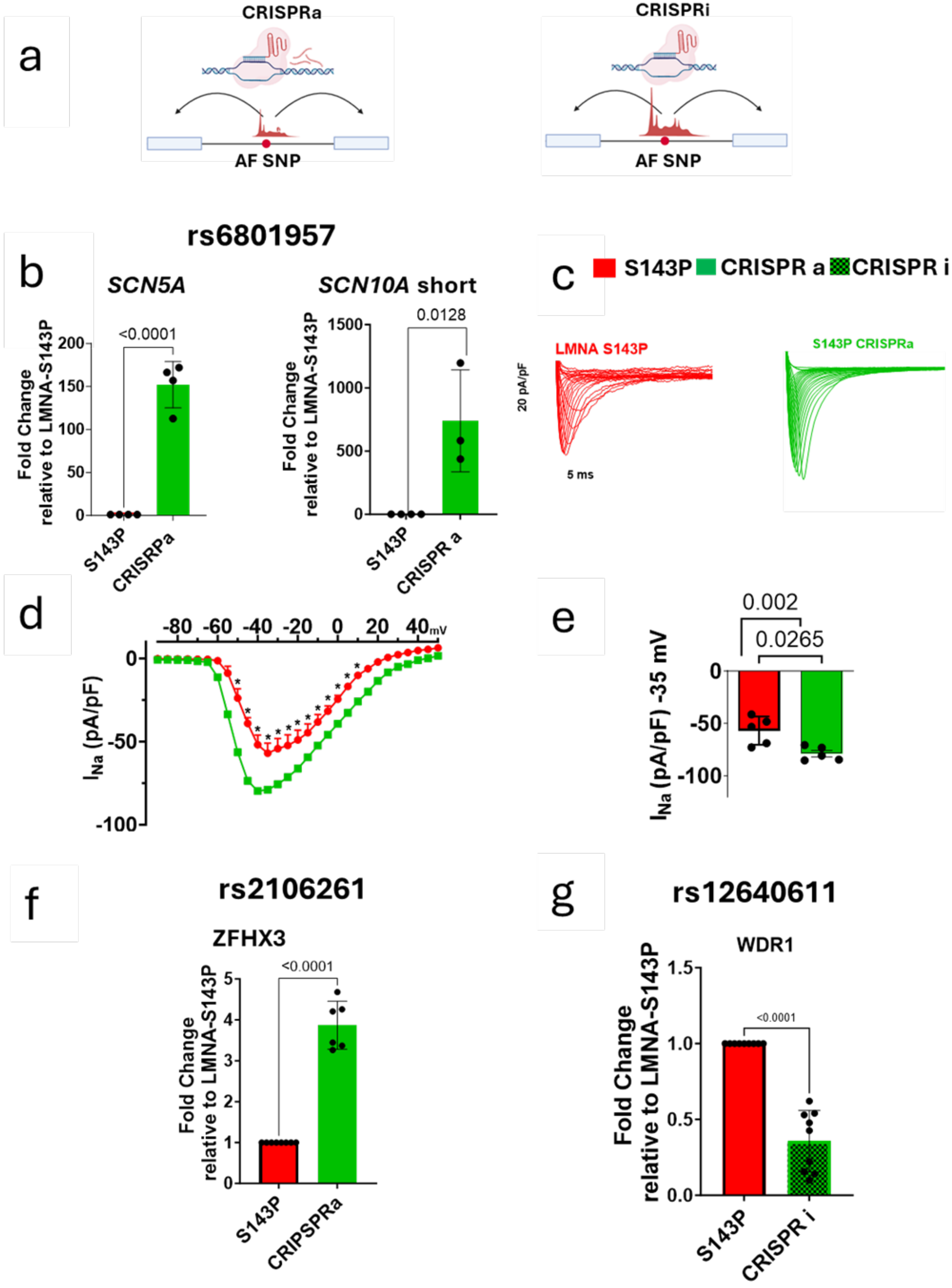
a
CRISPRa
AF SNP
CRISPRi
AF SNP
b
rs6801957
SCN5A
Fold Change relative to LMNA-S143P
<0.0001
S143P
CRISRPa
SCN10A short
0.0128
S143P
CRISPR a
c
S143P
CRISPR a
CRISPR i
LMNA S143P
20 pA/pF
5 ms
S143P CRISPRa
d
mV
I_Na (pA/pF)
e
0.002
0.0265
I_Na (pA/pF) -35 mV
f
rs2106261
ZFHX3
<0.0001
Fold Change relative to LMNA-S143P
S143P
CRIPSPRa
g
rs12640611
WDR1
<0.0001
Fold Change relative to LMNA-S143P
S143P
CRISPR i

Fig. 6: Functional interrogation of variant regulatory regions in differentially accessible regions by CRISPR epigenetic assays. a. Schematic showing the CRISPR activation(i)(a)/interference design. **b**. Reduced accessibility at the *SCN10A* intronic enhancer inhibits *SCN5A* transcription. CRISPRa restores expression of *SCN5A* and *SCN10A* short; bar graph quantifying changes in expression by qPCR. n=4 replicates representative of three independent differentiations, ordinary one-way ANOVA with multiple comparisons, p<0.05)**. c.** Representative I_{Na} traces show restoration of current after CRISPRa**. d.** Current-voltage (I/V) curve showing reduced I_{Na} in *LMNA p.S143P* iPSC-aCMs, rescued by transfection of CRISPRa complexes. **e.** I/V curve quantified in a bar graph. (n = 4-6 cells per group; unpaired t-test with Welch's correction p<0.05). **f.** CRISPRa of the *ZFHX3* variant region increases ZFHX3 expression **g.** CRISPRi of enhancer in *WDR1* reduces its expression
[Editorial note: panel a Created in BioRender. Owais, A. (2026) https://BioRender.com/xntq64i]

9) The section about "Risk of Incident AF in carriers of LMNA protein-altering..." is not clear to me. Is the effect of PRS and PAVs synergistic, additive, less than additive? How does the information regarding a patients PRS and PAVs contribute to risk assessment?

<u>Response</u>: We thank the reviewer for this comment and have clarified this section in the revised manuscript. Our analysis examined the interaction between *LMNA* PAVs and PRS for AF in the UK Biobank and the All of Us cohorts. We found that both *LMNA* PAVs and higher PRS were independently associated with increased risk of incident AF. When modeled together, their combined effect on AF risk was consistent with an additive model rather than synergistic or less-than-additive, supported by parallel risk trajectories across PRS strata and no evidence of statistical interaction between the two factors (interaction term p = 0.86).

In practical terms, this means that a patient with an *LMNA* PAV and a high PRS has a higher absolute risk than someone with only one of these risk factors, but the magnitude of combined risk is consistent with the sum of their individual effects.

From a clinical perspective, incorporating both PRS and rare-variant status could improve AF risk stratification by identifying individuals at particularly high risk, such as those with a pathogenic *LMNA* variant and a high PRS, who might benefit from earlier monitoring or preventive strategies. We have revised the text to explicitly state the additive nature of the effect and its potential implications for risk assessment.

Results (<u>page 6, lines 132-135</u>):

> "The results show that *LMNA* PAVs and PRS contributed independently to AF risk. The combined effect was consistent with an additive model, supported by parallel risk projectories across PRS strata and no evidence of statistical interaction between the two factors (interaction term p = 0.86)."

Discussion (<u>page 18, lines 404-416</u>):

> "We applied an AF PRS in two large population cohorts, the UK Biobank and AoU. Carriers of rare *LMNA* variants with a high PRS had a two-fold increased risk of early-onset AF compared to those with a lower PRS, particularly in individuals under 65. While prior studies suggest that common variants influence the penetrance of rare monogenic variants, our findings provide direct molecular evidence supporting this interaction[8,9]. Our findings suggest that *LMNA* PAVs and polygenic risk contribute independently to AF risk in an additive manner. Even in the absence of synergistic interaction, their combined effect can increase absolute risk, supporting a more comprehensive approach to precision medicine and targeted screening strategies. Clinically, incorporating both PRS and rare variant status could improve AF risk stratification by identifying individuals at high risk, such as those with both a pathogenic *LMNA* variant and a high PRS, who might benefit from earlier monitoring or preventive strategies."

Reviewer #3

In this study, the authors hypothesized that a rare LMNA variant may alter chromatin accessibility and gene expression at AF-risk loci. They conducted chromatin accessibility and gene expression profiling in iPSC-aCMs generated from a proband carrying the LMNA-S143P variant. This analysis identified significantly altered chromatin accessibility at eight AF associated loci, including a SNP within a known SCN5A enhancer. SCN5A expression and INa were consistently reduced, impairing atrial conduction and promoting re-entry. They employed CRISPR-based approaches to correct the LMNA-S143P variant, activate the SCN5A promoter, and enhance SCN10A regulatory activity. These interventions successfully restored SCN5A expression and INa. They applied a PRS for AF to carriers of rare LMNA PAVs in the UK Biobank and All of Us (AoU) cohorts. Individuals with a high PRS exhibited a two-fold increased risk of early-onset AF compared to those with a lower PRS emphasizing the need to integrate rare and common genetic variations for improved AF risk stratification. They highlighted the need for comprehensive genetic assessment, incorporating both rare and common variants, not only for risk prediction but also for early intervention to prevent atrial myopathy and its complications. This is an interesting manuscript, however, several concerns need to be addressed to enhance the importance of this study.

1) Please indicate the age at which each of the following episodes was observed.

- ***- underwent implantation of a biventricular pacemaker (Page 5, lines 16-17)***
- ***- required pacemakers (Page 6, line 1)***
- ***- underwent heart transplantation due to CHF (Page 6, line 2)***

Response: We thank the reviewer for this request and have now included the ages at which each clinical event occurred in the revised manuscript. Specifically, the proband underwent biventricular pacemaker implantation at age 41, and several family members underwent pacemaker implantation in their late 30s and early 40s. One family member (II-4) underwent heart transplantation due to congestive heart failure at age 48. These details have been added to the Clinical Characteristics section (**Page 7-8**, lines 172-180) for clarity and completeness.

2) Two main arrhythmogenic mechanisms including atrial reentry and ectopic atrial activity are considered to lead to AF. Please provide evidence or a detailed discussion on how the following findings are involved in the development of AF. Furthermore, discuss and describe which finding has the greatest impact on the development of AF.

- ***▪ Structural genes associated with early onset-AF such as MYL4, TTN, MYBPC3, DSP and PKP2 were significantly downregulated.***
- ***▪ Gene enrichment identified altered cardiovascular signaling pathways, including upregulation of DCM, β-adrenergic signaling.***
- ***▪ Downregulation of the cardiac hypertrophy and differentiation pathways.***
- ***▪ The DNA damage repair pathway was upregulated***
- ***▪ Reduced accessibility at enhancers of genes encoding transcription factors, TBX5, GATA4, GATA6, and the atrial-specific gene, NPPA***
- ***▪ CEP68 was upregulated***
- ***▪ NPTN was significantly downregulated***
- ***▪ SGK1 was significantly downregulated***
- ***▪ Calcium transient measurements revealed that LMNA-S143P iPSC-aCMs exhibited reduced transient amplitude and duration and irregular transient intervals***

Response: We thank the reviewer for this critical comment and have expanded our Results and Discussion to address the relationship of each finding to AF pathogenesis.

In *LMNA* p.S143P iPSC-aCMs, we observed coordinated molecular and functional changes that together create a substrate for AF through both reentry and ectopic activity. Downregulation of key sarcomeric and desmosomal genes (*MYL4, TTN, MYBPC3, DSP, PKP2*)[10-12] is predicted to weaken atrial structural integrity, disrupt mechanoelectrical coupling, and increase conduction heterogeneity. At the same time, upregulation of DCM and β-adrenergic signaling pathways promotes maladaptive remodeling and calcium-dependent triggered activity. Suppression of cardiac hypertrophy and differentiation pathways suggests impaired maturation and reduced conduction reserve, and activation of the DNA damage repair pathway reflects chronic cellular stress that may drive fibrosis and conduction slowing. Reduced chromatin accessibility at enhancers of *TBX5, GATA4, GATA6,* and *NPPA* likely decreases expression of downstream targets, including *SCN5A*, thereby impairing sodium current and slowing conduction. Additional transcriptional changes, including upregulation of *CEP68*[13], downregulation of *NPTN*[14] and *SGK1*[15], may further disrupt cytoskeletal organization, calcium handling, and ion channel regulation. Functionally, these alterations are accompanied by reduced calcium transient amplitude and duration, along with irregular intervals, consistent with impaired calcium cycling that can trigger ectopic firing.

While multiple findings contribute to arrhythmogenic potential, we believe that reduced enhancer accessibility at *TBX5* and other atrial TFs, leading to impaired sodium current via *SCN5A* downregulation, represents the dominant driver of AF risk in this model. This change directly affects conduction velocity and reentry susceptibility, integrating upstream chromatin remodeling with downstream transcriptional, EP, and calcium-handling alterations. Collectively, these results indicate that *LMNA* p.S143P disrupts both structural and EP homeostasis in atrial cardiomyocytes, creating a substrate that facilitates both initiation and maintenance of AF.

The revised Discussion now includes this expanded mechanistic framework, linking each molecular and cellular finding to established AF mechanisms (pages 20-21, lines 458-478).

> "In *LMNA* p.S143P iPSC-aCMs, we observed coordinated molecular and functional changes that together create an arrhythmogenic substrate supporting both atrial reentry and ectopic activity. Downregulation of sarcomeric and desmosomal genes associated with early-onset AF (*MYL4, TTN, MYBPC3, DSP,* and *PKP2*) is predicted not only to weaken atrial structural integrity but also to increase conduction heterogeneity, favoring reentry[10-12]. At the same time, enrichment of DCM and β-adrenergic signaling pathways is consistent with maladaptive remodeling and calcium-dependent triggered activity. Suppression of cardiac hypertrophy and differentiation programs suggests impaired atrial maturation and reduced conduction reserve, and activation of DNA damage repair pathways reflects chronic cellular stress that may further promote fibrosis and conduction slowing. Reduced chromatin accessibility at enhancers modulating key atrial TFs (*TBX5, GATA4, GATA6*) and *NPPA* likely propagates widespread transcriptional dysregulation of downstream effector genes, including *SCN5A*, thereby impairing I_{Na} and slowing atrial conduction. Additional changes, such as upregulation of *CEP68*[13] and downregulation of *NPTN*[14] and *SGK1*[15], may further disrupt cytoskeletal organization, ion channel regulation, and calcium handling, consistent with the observed reductions in calcium transient amplitude and duration and increased irregularity. While multiple pathways contribute to arrhythmogenesis, impaired atrial conduction driven in part by chromatin-mediated disruption of TF networks, particularly TBX5 and its downstream sodium channel targets, emerges as a dominant mechanism, linking *LMNA*-mediated chromatin remodeling to atrial myopathy and AF."

References Cited in the Response to the Reviewer Comments

1 Miyazawa, K. *et al.* Cross-ancestry genome-wide analysis of atrial fibrillation unveils disease biology and enables cardioembolic risk prediction. *Nature Genetics* **55**, 187-197, doi:10.1038/s41588-022-01284-9 (2023).

2 Armaroli, A. *et al.* Lamin A/C Missense Mutation R216C Pinpoints Overlapping Features Between Brugada Syndrome and Laminopathies. *Circulation: Genomic and Precision Medicine* **13**, e002751, doi:10.1161/CIRCGEN.119.002751 (2020).

3 Olaopa, M. A. *et al.* Phosphorylation of Lamin A/C at serine 22 modulates Na(v) 1.5 function. *Physiol Rep* **9**, e15121, doi:10.14814/phy2.15121 (2021).

4 Salvarani, N. *et al.* The K219T-Lamin mutation induces conduction defects through epigenetic inhibition of SCN5A in human cardiac laminopathy. *Nat Commun* **10**, 2267, doi:10.1038/s41467-019-09929-w (2019).

5 Selewa, A. *et al.* Single-cell genomics improves the discovery of risk variants and genes of atrial fibrillation. *Nature Communications* **14**, 4999, doi:10.1038/s41467-023-40505-5 (2023).

6 van Ouwerkerk, A. F. *et al.* Identification of Functional Variant Enhancers Associated With Atrial Fibrillation. *Circ Res* **127**, 229-243, doi:10.1161/circresaha.119.316006 (2020).

7 van Ouwerkerk, A. F. *et al.* Identification of atrial fibrillation associated genes and functional non-coding variants. *Nat Commun* **10**, 4755, doi:10.1038/s41467-019-12721-5 (2019).

8 Ritchie Marylyn, D. *et al.* Chromosome 4q25 Variants Are Genetic Modifiers of Rare Ion Channel Mutations Associated With Familial Atrial Fibrillation. *Journal of the American College of Cardiology* **60**, 1173-1181, doi:10.1016/j.jacc.2012.04.030 (2012).

9 Vad, O. B. *et al.* Rare and Common Genetic Variation Underlying Atrial Fibrillation Risk. *JAMA Cardiol* **9**, 732-740, doi:10.1001/jamacardio.2024.1528 (2024).

10 Orr, N. *et al.* A mutation in the atrial-specific myosin light chain gene (MYL4) causes familial atrial fibrillation. *Nat Commun* **7**, 11303, doi:10.1038/ncomms11303 (2016).

11 Ghazizadeh, Z. *et al.* Metastable Atrial State Underlies the Primary Genetic Substrate for MYL4 Mutation-Associated Atrial Fibrillation. *Circulation* **141**, 301-312, doi:10.1161/CIRCULATIONAHA.119.044268 (2020).

12 Phadke, K. *et al.* Atrial cardiomyopathy resulting from loss of plakophilin-2 expression: Response to adrenergic stimulation and implications for the exercise response. *J Physiol*, doi:10.1113/JP286985 (2024).

13 Pagan, J. K. *et al.* Degradation of Cep68 and PCNT cleavage mediate Cep215 removal from the PCM to allow centriole separation, disengagement and licensing. *Nat Cell Biol* **17**, 31-43, doi:10.1038/ncb3076 (2015).

14 Owczarek, S. & Berezin, V. Neuroplastin: cell adhesion molecule and signaling receptor. *Int J Biochem Cell Biol* **44**, 1-5, doi:10.1016/j.biocel.2011.10.006 (2012).

15 Bapat, A. *et al.* Genetic inhibition of serum glucocorticoid kinase 1 prevents obesity-related atrial fibrillation. *JCI Insight* **7**, doi:10.1172/jci.insight.160885 (2022).

Responses to Remaining Reviewer Comments

Reviewer #1

The responses and additional experiments from the authors is appreciated.
The specific mechanisms being proposed would be more solidified by having demonstrated similarities, at least in genotype data at AF regulatory loci, for all 4 cases in the original family, particularly at the SCN10A regulatory locus which is a focus of this paper. Also, would have been enhanced by UK biobank data of LMNA-AF cases (rather than PRS in general).
The addition of a second mutation is useful, but this is limited to showing downregulation of SCN5A and TBX5, rather than genotype data at AF regulatory loci.

The entire focus on the SCN10A locus comes from the proband (1/4 cases). The UK Biobank data focuses on PRS, rather than LMNA-AF and the SCN10A locus. This disconnect is curious.

Response: We thank the reviewer for these insightful comments and have revised the manuscript accordingly.

1. Genotype concordance at AF regulatory loci (including SCN10A)
We agree that concordance across affected individuals strengthens the mechanistic interpretation. We provide genotype data at the SCN10A locus (rs6801957) in three affected individuals (proband, mother, and an additional LMNA carrier) (**Fig. 5a, Fig. S7, Fig. 8c,e**). The mother is homozygous for the risk allele, indicating that all offspring, including the affected sibling, carry at least one risk allele at this locus.

Importantly, SCN10A is presented as a representative locus. Our ATAC-seq data demonstrate altered chromatin accessibility across multiple AF-associated regulatory regions, indicating that LMNA variants broadly modulate regulatory landscapes rather than acting at a single locus. We have highlighted these changes in the discussion, page 20 lines 450-456, 461-464.

2. PRS vs. LMNA-AF–specific analyses
We acknowledge the reviewer's point regarding LMNA-AF–specific analyses. However, due to the rarity of LMNA pathogenic variants, such analyses are currently underpowered. We therefore used PRS in LMNA variant carriers within the UK Biobank to capture the aggregate effect of common AF-associated variants.

Our experimental data provide a mechanistic basis for these observations: LMNA variants alter chromatin accessibility at AF-associated loci, thereby modulating the transcriptional impact of common variants. Thus, PRS reflects cumulative risk, while our functional studies explain the underlying mechanism.

3. Focus on SCN10A and generalizability
We focused on SCN10A because it showed a robust and reproducible effect on chromatin accessibility, gene expression (SCN5A), and electrophysiologic phenotype. To address generalizability, we extended our findings to an independent LMNA variant (G449V), demonstrating similar regulatory and transcriptional effects (**Fig. 8c–e**).

In addition, genome-wide analyses and CRISPR perturbation experiments show that multiple AF-associated regulatory loci influence gene expression, supporting a broader model in which LMNA variants sensitize the genome to common risk variants.

4. Second LMNA variant and regulatory genotype data
While genotype data across all AF loci were not available for the second variant, the consistent transcriptional and functional effects (SCN5A and TBX5 downregulation) across distinct LMNA variants support a shared mechanism. Larger cohorts will be required to systematically assess genotype–phenotype relationships across loci.

Summary
We have clarified that SCN10A serves as a mechanistically validated example within a broader set of affected loci, and that PRS and functional data are complementary, linking cumulative genetic risk to LMNA-mediated chromatin remodeling.
